# Green synthesis of new chiral amino acid urea derivatives and evaluation of their anti-inflammatory activity

**DOI:** 10.1039/d5ra04473a

**Published:** 2025-09-30

**Authors:** Sirine Mehrez, Sarah Absi, Assia Hamdi, Sylvain Marque, Abderrahman Bouraoui, Yakdhane Kacem, Jamil Kraiem

**Affiliations:** a Laboratoire de Développement Chimique, Galénique et Pharmacologique des Médicaments, Faculté de Pharmacie de Monastir, Université de Monastir Rue Avicenne 5000 Monastir Tunisia; b Université Paris-Saclay, UVSQ, CNRS, UMR 8180, Institut Lavoisier de Versailles (ILV) 45 Avenue des Etats-Unis 78 035 Versailles Cedex France sylvain.marque@uvsq.fr

## Abstract

A practically simple, mild and efficient method is developed for the synthesis of a series of new chiral urea derivatives by nucleophilic addition of amines from natural (l)-amino acids to various aryl isocyanates in alkaline aqueous medium, without organic co-solvent and under room temperature conditions. The synthesized compounds were obtained in good to excellent yields with high chemical purity by applying simple filtration and their structures were confirmed by spectral analysis (^1^H, ^13^C NMR and HRMS). Molecular docking analysis revealed strong hydrogen bonding interactions between the synthesized compounds and COX-1 (PDB ID: 6Y3C) and COX-2 (PDB ID: 5KIR), with binding energies ranging from −6.2 to −9.6 kcal mol^−1^, compared to the reference drug diclofenac (−7.4 kcal mol^−1^ for COX-1 and −8.4 kcal mol^−1^ for COX-2). This study showed interesting anti-inflammatory activities. The selected compounds were subjected to *in vivo* study for their anti-inflammatory activity. The *in vivo* results were correlated with molecular docking studies, supporting the prediction that the *in silico* binding affinities are in good agreement with the observed anti-inflammatory activity. Among the tested compounds, 1e evaluated at 25 mg kg^−1^ exhibited much better anti-inflammatory activity (edema inhibition = 97.05%) as compared to the standard drug diclofenac (edema inhibition = 63.82%). *In silico* ADMET, toxicity, and physicochemical properties revealed that the candidate compounds have acceptable values of drug-likeness.

## Abstract 2

Green synthesis of new chiral amino acid urea derivatives in high yields. The *in silico* study showed interesting anti-inflammatory activities and acceptable values of drug-likeness. The *in vivo* results are in good agreement with the molecular docking analysis.

## Graphical abstract

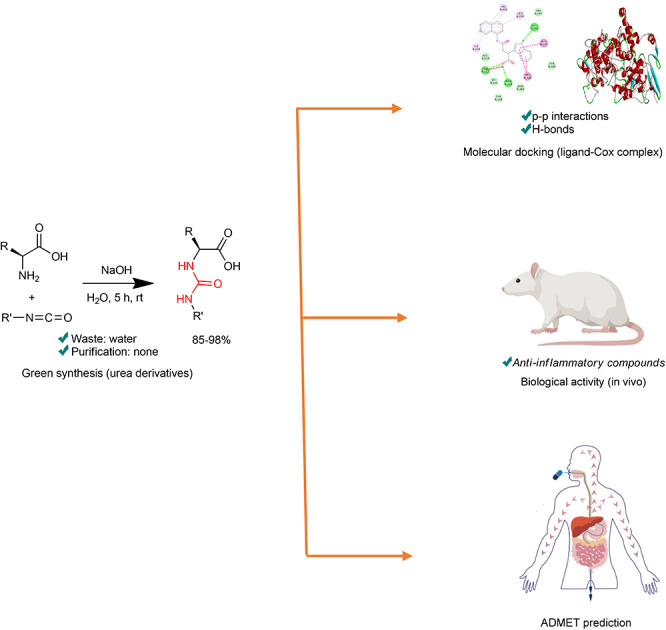

## Introduction

**Fig. 1:**
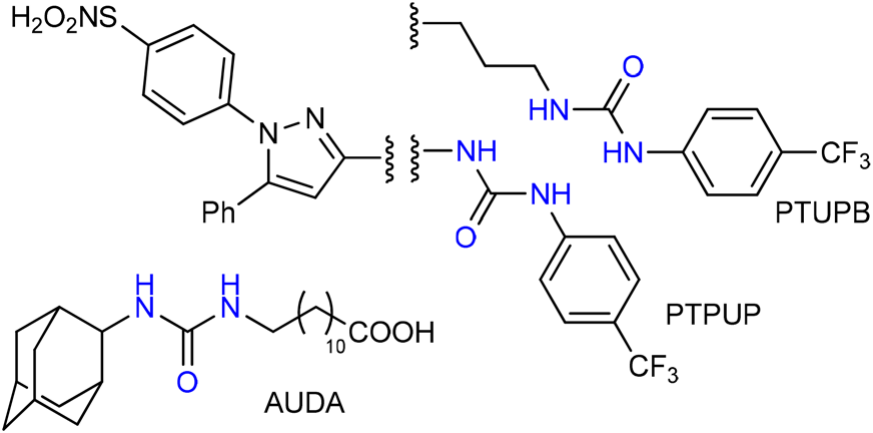
Examples of some urea molecules with anti-inflammatory activity.

Since the synthesis of urea was realized by Friedrich Wöhler in 1828 and due to its importance in organic chemistry,^1,2^ urea and its derivatives represent well-established privileged structures in a variety of areas, such as biological studies,^3^ analytical chemistry,^4^ polymer sciences,^5^ pharmaceuticals^6^ and agrochemicals.^7^ The urea functionality is intrinsic to many bioactive compounds, including several clinically approved therapies. Urea derivatives play a central role in drug design^8^ and medicinal chemistry^9^ thanks to their unique ability to establish stable hydrogen bonds with recognition elements of biological targets, such as proteins and receptors. These interactions drive specific biological activities, influence drug actions, and contribute to essential drug properties. In recent years, urea compounds have received more attention due to their potential as promising candidates for diverse therapeutic applications, including anti-inflammatory,^10^ anticancer,^11^ antidiabetic,^12^ antiviral,^13^ anticonvulsant,^14^ antifungal,^15^ and antibacterial activities.^16^ Inflammation is part of the defense mechanism of the immune system considered as the leading sign in several diseases, including atherosclerosis,^17^ as well age related diseases^18^ and several other infectious cases, which can prevent damage caused by harmful stimuli. Inflammatory diseases are highly prevalent all over the world and account for a significant proportion of global mortalities.^19^ Non-steroidal anti-inflammatory drugs (NSAIDs) are the most commonly for treating the action of inflammation and pain through non-selective inhibition of both the cyclooxygenase enzyme (COX).^20^ Indeed, urea derivatives serve as the central structural block of numerous biologically active compounds including AUDA that regulate the inflammation in the atherosclerotic lesions by CYP 450 pathway,^21^ the 4-(5-phenyl-3-{3-[3-(4-trifluoromethyl-phenyl)-ureido]-propyl}-pyrazol-1-yl)benzene sulfonamide PTUPB which is effective against inflammation^22^ and the 4-(1-phenyl-3-(3-(4-(trifluoromethyl)phenyl)ureido)-1*H*-pyrazol-5-yl)benzenesulfonamide PTPUP as a potent dual COX-2/sEH inhibitor with good anti-inflammatory/analgesic activity^23^ (Fig. 1). Multiple studies have confirmed the significant anti-inflammatory activity of urea derivatives.^24–29^ For example, Abdelazeem *et al.* highlighted the importance of the urea moiety linked to a secondary pharmacophore (diarylpyrazole), demonstrating significantly enhanced anti-inflammatory activity against edema.^30^ These observations, coupled with our continued interest in designing bioactive molecules from amino acids,^31–34^ prompted us to synthesize a novel series of urea derivatives and investigate their anti-inflammatory potential.

## Results and discussion

### Synthesis

From an ecological point of view, new indicators of the efficiency of green processes have been introduced such as the atom economy and environmental factor. The atom economy (AE)^35^ is the quantity defined as the weighted ratio of the molar mass of the desired product to the sum of the molar masses of the reactants:
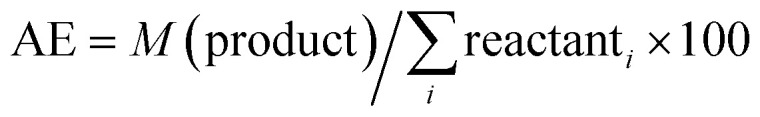


We define, also, the environmental factor (E-factor) introduced by Roger Sheldon.^36^ It is the ratio of the total mass of waste to the mass of product:
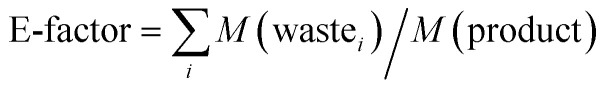


The environmental factor highlights the importance of minimizing waste generated during chemical synthesis. The ideal value must be as low as possible, tending towards zero. In our case, the green method employing water as the solvent is highly efficient, achieving a maximum atom economy (AE = 100%) and an excellent E-factor ranging from 0.15 to 0.25, with NaCl as the only by-product. It is worth noting that these values correspond to the full reality of experiments since each reactants are introduced in 1 : 1 stoichiometric quantity. We can therefore conclude that our green and eco-friendly synthetic approach is of great interest, since we were able to obtain good yields by using sodium hydroxide as a base and water as a solvent, leading to urea derivatives in AE and E-factor close to the perfection.

**Scheme 1:**
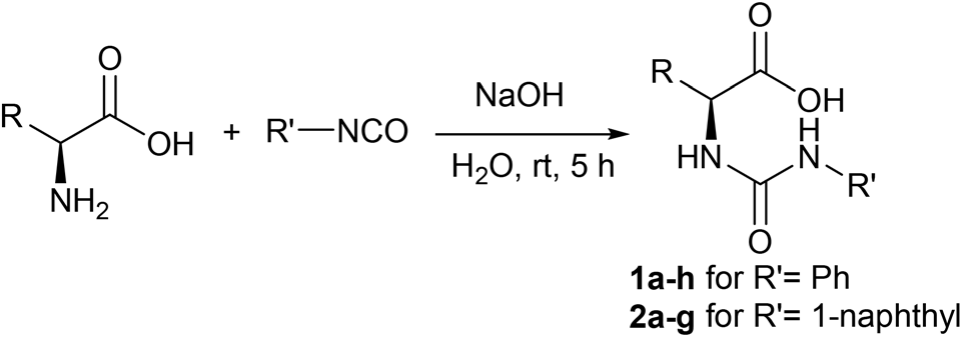
Synthesis of compounds 1a–h and 2a–g from natural amino acids.

The literature highlights a variety of synthetic methods for urea compounds. For example, a series of α-methyl-l-DOPA urea derivatives was synthesized with various aryl isocyanates using triethylamine as a base in THF under reflux conditions.^37^ A series of urea dipeptides was synthesized with isocyanates in the presence of bases such as *N*-ethyldiisopropylamine (DIEA) or *N*-methylmorpholine (NMM) in DCM,^38^ and a series of *N*′-substituted ureas was synthesized with isocyanates in the presence of sodium hydride dissolved in THF.^24^ Our approach is radically different, as it integrates environmental concerns from the design stage. Thus, urea derivatives 1a–h and 2a–g have been synthesized in a single step from the reaction of (l)-amino acids with aryl isocyanates in the presence of NaOH and water solvent at room temperature (Scheme 1). The reaction was completed in about 5 h. All the compounds 1a–h and 2a–g have been separated out as solids.

The use of isocyanates for synthesizing urea derivatives is not only rapid, but efficient as the products are easily isolated. The main advantage of this reaction is its easy work-up, which allows the products to be obtained in good to excellent yields (Table 1). The compounds 1a–h and 2a–g were characterized by 1D NMR, 2D NMR and mass spectral analysis. The NMR spectra are clear with a good resolution. Although three labile protons are present in the general structure, all proton and carbon signals could be assigned using 1D and 2D NMR experiments. Taking the compound 1a (R = Me, R′ = Ph) for example, both aliphatic proton and carbon signals were easily attributed and corroborated with COSY and HSQC spectra. Thus, the (H, C) pairs are: (1.30, 18.2) ppm for the methyl group and (4.10–4.24, 48.0) ppm for the H–C of the amino acid residue. Direct reading of HSQC allows to determine the aromatic H–C pairs: (6.89[t, 1H], 121.3[1C]), (7.22[t, 2H], 128.7[2C]), (7.37[d, 2H], 117.6[2C]) ppm corresponding to CCHCH**CH**, CCH**CH**CH and C**CH**CHCH respectively. The HSQC indicates that the proton signals at 6.45 and 8.60 ppm are carbon-free. The COSY spectra shows a correlation between 4.10–4.24 and 6.45 ppm which involves the attribution of 6.45 ppm for the CH–**NH** moiety. The Dept135 spectra allows to identify by subtraction from the ^13^C spectra the quaternary carbons: 140.2, 154.7 and 174.9 ppm. The HMBC shows that there is only one ^2^*J* interaction from the signal at 4.10–4.24 ppm with 174.9 ppm. This fixes the assignment of the carbon of the carboxylic acid at 174.9 ppm, which is confirmed in HMBC by ^3^*J* interaction with the signal at 1.30 ppm. The more distant correlation in HMBC between 4.10–4.24 and 154.7 ppm, which corresponds to a ^3^*J*, gives the assignment of the carbon signal of the urea function at 154.7 ppm. The last quaternary carbon is therefore aromatic at 140.2 ppm, which is consistent when looking at the HMBC correlation in ^2^*J* with the doublet signal at 7.37 ppm. It is worth noting that these final two correlations allow for the assignment of each aromatic proton in the phenyl group at *δ* 7.22 and 7.37 ppm, regardless of the previously determined ^1^H NMR spectral data. Once again, the HMBC spectrum demonstrates its utility by revealing a weak long-range (^4^*J*) correlation between the signals at *δ* 117.6 and 8.60 ppm, supporting the assignment of the carbon-deficient signal at 8.60 ppm to the NH proton of the NHCONHPh group.

**Table 1:** Synthesis of products 1a–h and 2a–g

Compounds	R	Yield (%)
1a	–CH_3_	98
1b	–CH_2_CONH_2_	86
1c	–CH_2_CH(CH_3_)_2_	90
1d	–CH_2_–Ph	94
1e	–Ph	95
1f	–CH_2_-indol	91
1g	–CH(CH_3_)_2_	88
1h	–(CH_2_)_2_SCH_3_	90
2a	–CH_3_	97
2b	–CH_2_CONH_2_	85
2c	–CH_2_CH(CH_3_)_2_	89
2d	–CH_2_–Ph	92
2e	–Ph	86
2f	–CH_2_-indol	93
2g	–CH(CH_3_)_2_	87

NMR analysis of compounds 1a–h and 2a–g reveals that the carboxylic acid proton signal is rarely well-defined or even detectable in the ^1^H NMR spectra. In the few cases where it is observed, such as in compounds 1f and 2f, it typically appears around 10.90 ppm. However, in most cases, this signal is either absent or overlaps with the residual water or HDO signals commonly found in commercial DMSO-*d*_6_, which resonate between 2.80 and 3.33 ppm (*e.g.*, 2d at 3.40 ppm). In some cases, the carboxylic acid proton exhibits an unusual downfield shift, overlapping with other signals at 3.50, 3.70, 3.80, or even at 4.50 ppm, as observed in 2b, 1c, 1d, and 1a, respectively. Consequently, integration of this signal in the ^1^H NMR spectrum is generally unreliable and not suitable for quantitative analysis. It is important to note that, among the three labile protons present in these compounds, the NH signals remain remarkably consistent, exhibiting minimal chemical shift variation despite differences in amino acid side chains, aromatic groups, solvent water content, or concentration. In DMSO-*d*_6_, the NH signal of the NHCONHAr moiety consistently appears around 6.50 ppm, while the NHCO–NHAr signal is found between 8.50 and 8.90 ppm. The presence of these two sharp, well-resolved signals provides strong and unambiguous evidence for the formation of the urea functionality.

### Molecular docking studies

#### Insight into the protein used

Cyclooxygenase (COX), also referred to as prostaglandin (PG)-endoperoxide synthase, were chosen for their central role in the production of prostanoids, including thromboxane and prostaglandins (PGs) like prostacyclin, from arachidonic acid (AA).^39^ The two isoenzymes involved in prostaglandin biosynthesis are COX-1 and COX-2 under the following codes: 6Y3C and 5KIR. The arachidonic acid (AA) pathway is a major component of the inflammatory response.^40^ COX-1 is continuously expressed in most tissues and is involved in maintaining normal physiological processes, whereas COX-2 is upregulated in response to inflammatory stimuli and is primarily responsible for mediating inflammation and pain responses.^41^ COX-2 expression is elevated during inflammatory conditions in response to pro inflammatory molecules such as IL-1, TNF-α, and LPS.^42^

#### Discussion of the different interactions formed

The synthesized compounds 1a–h and 2a–g subjected to molecular docking fitted well to ‘COX1 (6Y3C) and COX2 (5KIR)’ active sites inside the pocket and showed good binding energy scores ranging from −6.2 to −9.6 kcal mol^−1^ compared to the reference drug diclofenac (−7.4 kcal mol^−1^ (COX1), −8.4 kcal mol^−1^ (COX2)).

The results are presented in Table 2. The study focuses on the interaction between enzyme amino acids and synthesized products to form a stable complex. The aim is to predict the mode of interaction by determining the positioning of the ligand relative to its receptor and assessing ligand-receptor affinity. The best result corresponds to the lowest interaction energy in Δ*G*. The analysis of binding energies should be compared to the interaction visualization results. A higher number of interactions suggest deeper penetration of the ligand into the enzyme's active site.

**Table 2:** Docking binding energies (kcal mol^−1^)

Ligand	Binding energy of COX1 (Pdb: 6Y3C) (kcal mol^−1^)	Binding energy of COX2 (Pdb: 5KIR) (kcal mol^−1^)
1a	−8.3	−8.8
1b	−8.1	−7.8
1c	−7.3	−8.0
1d	−7.4	−7.6
1e	−8.7	−9.1
1f	−8.9	−9.2
1g	−6.9	−6.8
1h	−6.2	−7.3
2a	−8.2	−7.7
2b	−8.0	−7.6
2c	−7.8	−8.6
2d	−8.2	−8.3
2e	−8.1	−7.8
2f	−9.6	−9.5
2g	−7.8	−8.4
Diclofenac	−7.4	−8.4

**Fig. 2:**
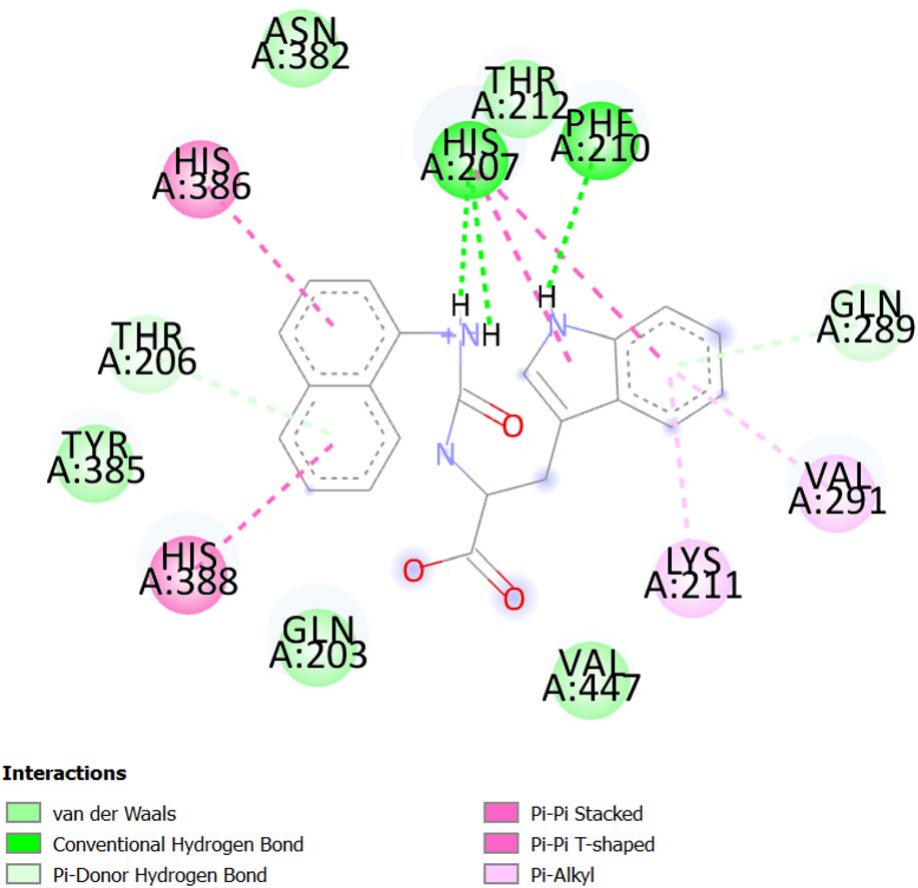
The corresponding 2D diagram of interactions of the compound 2f at the active site of COX-1.

**Fig. 3:**
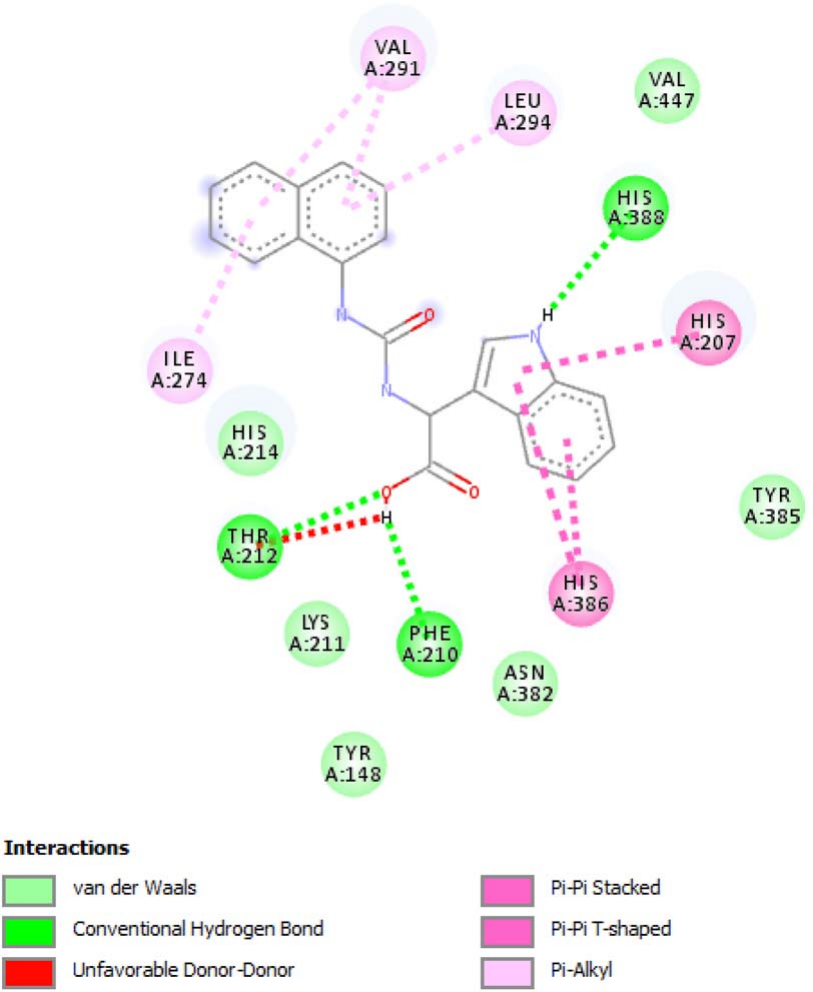
The corresponding 2D diagram of interactions of the compound 2f at the active site of COX-2.

The Fig. 2 and 3 display the 2D diagrams of the compounds with the lowest docking energies and the highest number of interactions, particularly hydrogen bonds, observed in the visualization. A comprehensive analysis of all the docked compounds showed that compound 2f exhibited the most favorable binding energy (−9.6 kcal mol^−1^) which displayed 3 H-bonds in addition to some hydrophobic interactions (Fig. 1).

The compound 2f was found to dock into the active site of COX1 formed a three conventional hydrogen bonds with HIS207 and PHF210. The compound 2f was also found to dock into the active site of COX-2 with an interaction energy of −9.5 kcal mol^−1^. It formed three hydrogen bonds with THR212, PHE210 and HIS388 in addition to other hydrophobic interactions such as Pi–alkyl with VAL291, LEU294, ILE274 and Pi–Pi T-shaped/Pi–Pi stacked interactions with HIS207, HIS386. While the analogue 1e was found to dock into the active site of COX-1, engaged mainly by the three H-bonds displayed with residues: HIS388 and ASN382, the compound 1e was also found to dock into the active site of COX-2 with an interaction energy of −9.1 kcal mol^−1^. Three hydrogen bonding interactions of 1e were observed with the bonding GLY225 and ASN375.

#### Anti-inflammatory activity

**Fig. 4:**
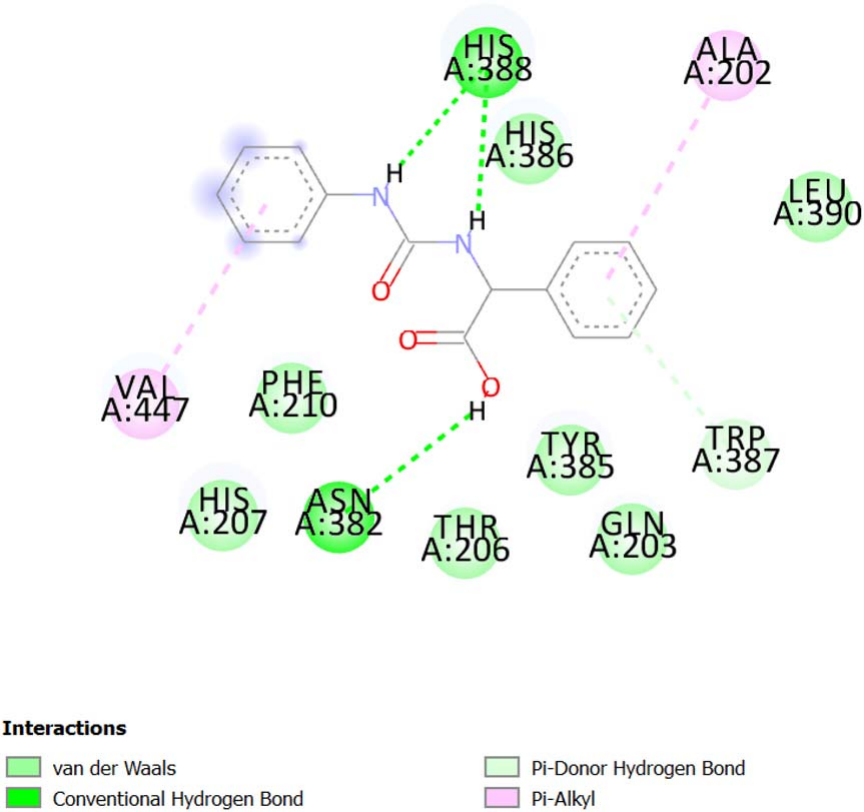
The corresponding 2D diagram of interactions of the compound 1e at the active site of COX-1.

**Fig. 5:**
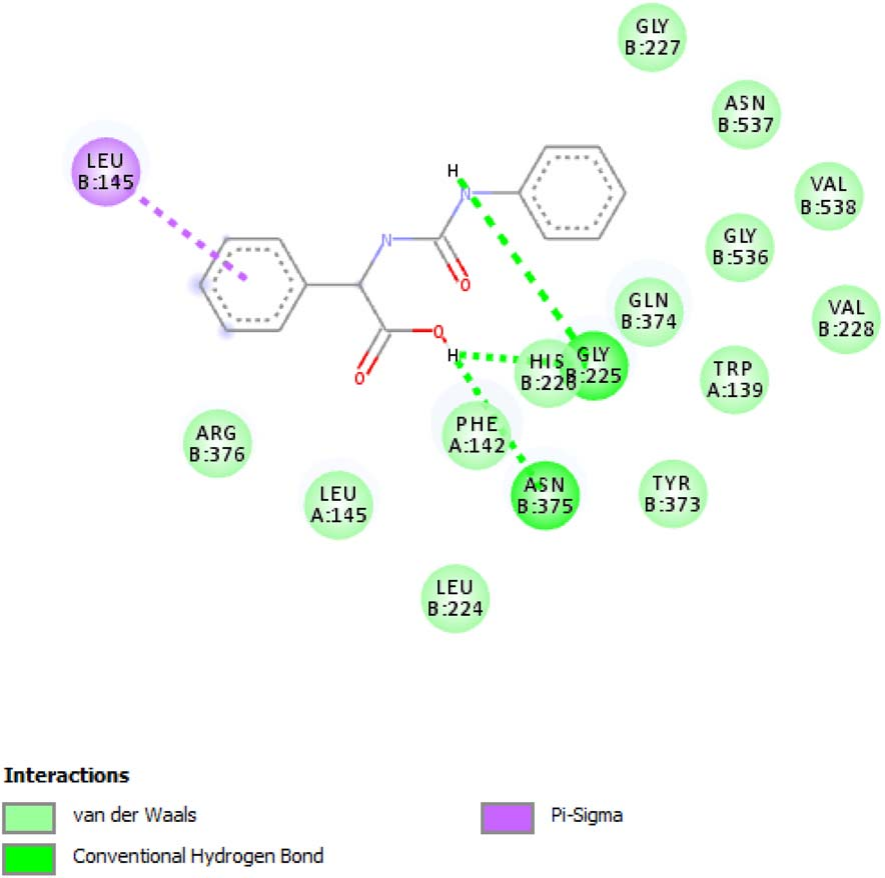
The corresponding 2D diagram of interactions of the compound 1e at the active site of COX-2.

Inflammation is a physiological response of the immune system to infections or injuries, involving various enzymatic and cellular processes aimed at protecting the body from damage. The anti-inflammatory potential of compounds chosen during the*in silico* study (1a, 1e, 1f, 2f) on carrageenan-induced rat paw edema determined by the method of Winter *et al.*^43^ is shown in Table 3. These results indicate that the compound 1e tested at 12.5 and 25 mg kg^−1^ i.p. exhibited much better anti-inflammatory activity as compared to the standard drug diclofenac since a significant reduction of the edema was noted even at the lower dose. This percentage increase in paw oedema by compound 1e at a dose of 25 mg kg^−1^ was observed to be 51.41% at 1 h, which progressively increased to 97.05% by 5 h. Its anti-inflammatory activity at the fifth hour exceeds that of the reference drug at both 12.5 mg kg^−1^ and 25 mg kg^−1^ doses, with inhibition percentages of 80.53% and 97.05%, respectively (Fig. 4 and 5).

**Table 3:** Anti-inflammatory activity evaluation of compounds 1a, 1e, 1f, 2f on carrageenan-induced rat paw edema, in comparison to the reference drug diclofenac

Sample	Dose (mg kg^−1^)	Edema volume (10^2^ mL) (*m* ± SEM)					% Of edema inhibition				
		1 h	2 h	3 h	4 h	5 h	1 h	2 h	3 h	4 h	5 h
Control^a^	25	1.71	2.57	4.01	3.83	3.40					
Control^a^	12.5 & 50	0.06	0.13	0.41	0.27	0.25					
1a	25	1.46 ± 0.13	1.60 ± 0.26	1.58 ± 0.04	1.44 ± 0.22	1.22 ± 0.14	13.58 ± 1.92	14.67 ± 0.85	45.48 ± 0.55	50.04 ± 1.19	51.43 ± 1.82
1a	50	1.42 ± 0.07	0.89 ± 0.06	0.79 ± 0.13	0.74 ± 0.07	0.66 ± 0.06	15.40 ± 0.03	50.75 ± 1.95	73.75 ± 0.55	74.35 ± 0.09	74.70 ± 0.82
1e	25	0.78 ± 0.14	0.26 ± 0.05	0.29 ± 0.09	0.18 ± 0.03	0.10 ± 0.06	51.41 ± 0.37	84.06 ± 0.72	91.23 ± 1.10	93.83 ± 0.67	97.05 ± 1.74
1e	12.5	1.55 ± 0.05	1.32 ± 0.06	1.15 ± 0.08	0.64 ± 0.08	0.51 ± 0.07	9.02 ± 0.63	27.06 ± 0.19	60.41 ± 6.68	77.83 ± 0.96	80.53 ± 0.62
1f	25	1.50 ± 0.17	1.60 ± 0.32	1.79 ± 0.16	1.70 ± 0.09	1.48 ± 0.13	10.18 ± 1.04	10.18 ± 1.04	39.99 ± 1.04	40.13 ± 2.02	41.18 ± 0.74
1f	50	1.47 ± 0.07	1.56 ± 0.13	1.85 ± 0.29	1.50 ± 0.12	1.32 ± 0.13	13.10 ± 1.56	15.43 ± 0.67	39.15 ± 1.13	48.14 ± 1.48	49.24 ± 1.86
2f	25	1.43 ± 0.24	1.28 ± 0.49	1.30 ± 0.46	1.43 ± 0.17	1.13 ± 0.09	11.58 ± 0.12	17.92 ± 0.47	47.83 ± 1.25	49.52 ± 1.20	55.26 ± 1.69
2f	50	1.38 ± 0.07	1.46 ± 0.10	0.96 ± 0.16	0.91 ± 0.08	0.71 ± 0.13	18.33 ± 1.69	19.88 ± 0.90	67.79 ± 1.36	68.36 ± 0.23	72.88 ± 1.81
Diclofenac^b^	25	1.53 ± 0.14	1.09 ± 0.20	1.31 ± 0.27	1.19 ± 0.09	0.91 ± 0.14	8.90 ± 0.74	42.63 ± 0.60	50.32 ± 0.27	58.00 ± 1.21	63.82 ± 1.54

a Control experiment.

b The diclofenac is the reference drug.

**Fig. 6:**
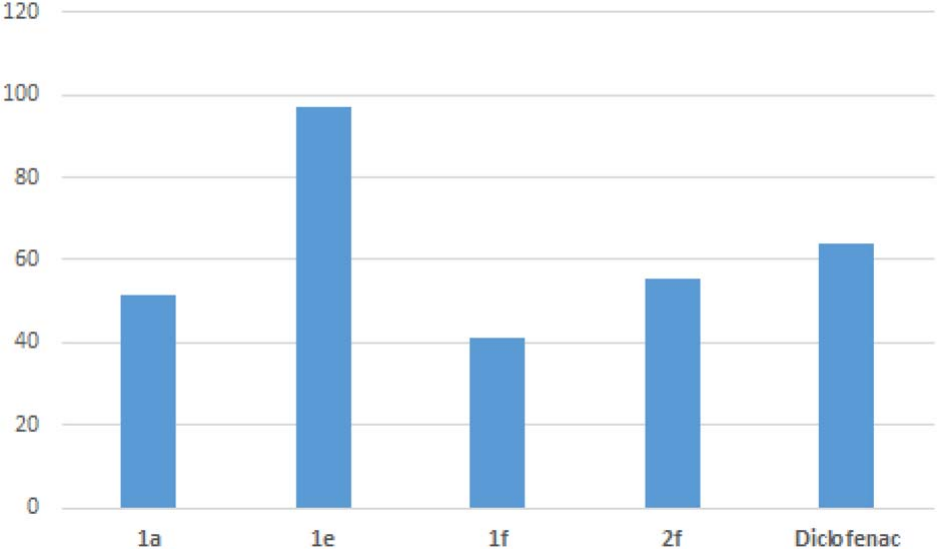
Percentage inhibition of edema for compounds 1a, 1e, 1f, 2f and the reference drug 5 h after carrageenan (25 mg kg^−1^ dose).

Also, edema inhibition was noted in the remaining compounds along the whole observation period. At 25 mg kg^−1^, the highest reduction of the edema was observed 5 h after carrageenan injection of three compounds of the series: 51.43% of inhibition for compound 1a, 41.18% of inhibition for compound 1f and 55.26% for compound 2f, whereas the reference drug (diclofenac, 25 mg kg^−1^) produced a reduction of 63.82% in paw volume (Table 3 and Fig. 6). At 50 mg kg^−1^, compounds 1a and 2f were more effective than at lower doses, reaching 73.75% and 67.79% inhibition at 3 h. On the other hand, the compound 1f consistently exhibited lower efficacy compared to diclofenac throughout the five-hour observation period, even at the 50 mg kg^−1^ dose.

#### SAR approach

The Structure–Activity Relationship study (SAR) indicates that the nature of substituents R and R′ play an important role in the anti-inflammatory activity of the structures of the synthesized compounds. In fact, the presence of an aromatic group at position R, instead of a aliphatic group, increase anti-inflammatory activity.^24^ The SAR also showed that the inhibitory potency and selectivity of the anti-inflammatory activity is dependent on the nature of the substituent on phenyl ring directly attached to a urea linker. The order of selectivity was OH > F > OMe > H ≈ Me > NHCOMe > Cl.^44^ This observation is consistent with literature findings, as studies on disubstituted urea's have shown that compounds lacking aryl ring substitution exhibit reduced or poor biological activity.^24^ Conversely, our study underscores the promising anti-inflammatory potential of compound 1e, which achieved an edema inhibition of 97.05%. This compound contains a urea moiety linked to an unsubstituted aryl ring. Additionally, compound 1a demonstrated significant activity, with 51.43% edema inhibition, despite featuring an aliphatic substituent at the R position and a phenyl group attached to the urea unit. Furthermore, replacing the phenyl ring in compound 1f (edema inhibition = 41.18%) with a naphthalene ring in compound 2f resulted in improved anti-inflammatory activity, achieving an edema inhibition of 55.26%.

#### ADMET: *in silico* studies profile

An *in silico* assessment of the highly active derivatives was carried out to evaluate their physicochemical properties and predict their ADMET profiles.^45^ Predictions were obtained using the pkCSM descriptor algorithm and the VEGA platform,^46^ and subsequently compared to Lipinski's Rule of Five.^47^ All results are summarized in Tables 4 and 5. *In silico* toxicity predictions indicate that compounds 1a, 1e, 1f, and 2f generally exhibit favorable safety profiles. All were predicted non-mutagenic by VEGA, with high applicability domain scores (>0.9) for 1a, 1e, and 1f, although 2f's predictions were inconclusive due to being outside the model's domain. Notably, compound 1f exhibited a potential for hepatotoxicity, warranting further experimental validation, whereas the other compounds, including 1a, 1e, 2f, and diclofenac, showed no hepatotoxic potential. Acute oral toxicity (LD_50_) values indicated moderate toxicity across all compounds, with 1e demonstrating slightly higher acute toxicity compared to diclofenac, while 1a and 2f displayed relatively lower toxicity. Chronic toxicity evaluation (LOAEL) suggested improved long-term tolerability for 1e and 1f in comparison to diclofenac and 1a. Moreover, none of the compounds posed significant risks of skin sensitization, and their predicted environmental toxicities were generally comparable.

**Table 4:** *In silico* mutagenicity prediction of compounds 1a, 1e, 1f, and 2f using CAESAR, ISS, and SarPy models with applicability domain and confidence metrics

Compound	Model	Mutagenicity result	Applicability domain (AD)	Accuracy index (AI)	Confidence index (CI)	Comments
1a	CAESAR	Non-mutagenic	>0.9	1 (High accuracy)	1	Within domain
	ISS	Non-mutagenic	>0.9	1	1	Within domain
	SarPy	Non-mutagenic	0.8	—	—	Out of domain
1e	CAESAR	Non-mutagenic	>0.9	1	—	Within domain
	ISS	Non-mutagenic	0.6	—	—	Out of domain
	SarPy	Non-mutagenic	>0.9	1	1	Within domain
1f	CAESAR	Non-mutagenic	>0.9	1	1	Within domain
	ISS	Non-mutagenic	>0.9	1	1	Within domain
	SarPy	Non-mutagenic	>0.9	1	1	Within domain
2f	CAESAR	Inconclusive	0.6	—	—	Out of domain
	ISS	Inconclusive	0.8	—	—	Out of domain
	SarPy	Inconclusive	0.6	—	—	Out of domain

**Table 5:** ADMET profile of compounds 1a, 1e, 1f and 2f

Parameters	1a	1e	1f	2f	Diclofenac
**Physicochemical properties**					
Molecular weight	208.217	270.288	323.352	373.412	296.153
Log *P*	1.2812	2.634	2.9853	4.1385	4.3641
Rotatable bonds	3	4	5	5	4
Acceptors	2	2	2	2	2
Donors	3	3	4	4	2
Surface area	87.108	115.800	138.056	160.738	120.331

**Absorption**					
Water solubility (log mol L^−1^)	−2.693	−3.789	−2.633	−5.494	−3.863
Caco-2 permeability (log *P*app)	0.845	0.724	0.002	0.817	1.379
Human intestinal absorption (%)	100	95.045	56.519	100	91.923
Skin permeability	−3.193	−2.732	−2.735	−3.103	−2.724

**Distribution**					
VDss (human)	−0.047	−1.729	−2.381	0.442	−1.605
Human unbound fraction (fu)	0.615	0.154	0.078	0.33	0
BBB permeability	−0.188	−0.581	−0.699	−0.324	0.236
CNS permeability	−2.950	−2.336	−2.339	−2.944	−1.97

**Metabolism**					
CYP interactions	None	None	None	Substrate (3A4) + inhibitor (2C19, 2C9)	None

**Excretion**					
Clearance	0.801	0.374	0.61	1.07	0.291

**Toxicity**					
AMES toxicity	Yes	No	No	No	No
Human max. Tolerated dose	0.389	0.962	0.651	−0.695	0.983
hERG inhibition	No	No	No	No	No
Acute toxicity (LD_50_)	3.015	1.954	2.496	3.079	2.405
Chronic toxicity (LOAEL)	0.715	1.676	2.691	1.06	1.562
Hepatotoxicity	No	No	Yes	No	No
Skin sensitization	No	No	No	No	No

Overall, compounds 1e and 2f emerge as promising candidates with favorable toxicity profiles, whereas 1f's hepatotoxic potential highlights the need for caution. Compound 1a, with its non-mutagenic profile and moderate toxicity, also represents a viable candidate for further development.

From the ADME perspective, all compounds comply with Lipinski's Rule of Five, indicating good oral drug-likeness. Log *P* values (1.28–4.36), hydrogen bond counts, and molecular weights are within acceptable ranges. However, solubility and permeability vary: 1f shows poor Caco-2 permeability, and 2f has the lowest solubility despite high intestinal absorption. All are P-gp substrates, with only 2f inhibiting P-gp I, CYP2C19, and CYP2C9, implying possible drug–drug interaction risks. None are predicted to significantly cross the BBB, reducing potential CNS side effects. Distribution and clearance parameters differ, with 2f showing higher tissue distribution and clearance rates. Overall, these findings support the further development of 1a, 1e, and 2f, with 1f requiring caution due to its hepatotoxicity signal.

Among the tested compounds, 1e and 2f displayed the most balanced safety and ADME profiles, combining non-mutagenicity, acceptable toxicity margins, and favorable drug-likeness parameters, making them strong candidates for further preclinical evaluation.

## Experimental

### General

Chemicals were purchased from Sigma-Aldrich chemical company and used without further purification. ^1^H NMR and ^13^C NMR spectra were recorded on Ultra shield Bruker Avance1 spectrometer 300 MHz using DMSO-*d*_6_ as the solvent. Chemical shifts (*δ*) are expressed in ppm relative to residual solvents (DMSO-*d*_6_: *δ*_H_ 2.50 *δ*_C_ 39.52). In all ^1^H NMR spectra, the number of protons for each signal and coupling constant values (Hertz) are indicated. Multiplicity is also reported and designated by the following abbreviations: s (singlet), d (doublet), m (multiplet). High-resolution mass spectrometry (HRMS) was recorded on a UPLC waters device spectrometer. Melting points were measured with a Wagner & Munz instrument.

### General procedures

#### Synthesis of new chiral amino acid derivatives 1a–h and 2a–g

In a flask, 5 mL of water and 200 mg of NaOH (5 mmol) were stirred until the base was fully dissolved, then 5 mmol of the amino acid were added under stirring. The medium was cooled to 0 °C (ice bath). 5 mmol of isocyanate was gradually added to the reaction mixture while maintaining the temperature at 0 °C. The reaction mixture was stirred for 5 minutes at 0 °C, after which it was allowed to proceed at room temperature for 5 hours. The reaction was then quenched by the addition of 4–5 mL of 1 M HCl until the pH reached 1–2, leading to the formation of a white precipitate. The product was isolated by performing vacuum filtration, without further purification.

(*S*)-2-(3-Phenylureido)propanoic acid (1a): white solid (439 mg, 5 mmol, 98%); *R*_f_ = 0.68 (90 : 10 EtOAc/CH_3_OH); mp = 176 ± 2 °C; [α]_D_^20^ = +9.5 (*c* = 1, MeOH); ^1^H NMR (300 MHz, DMSO-*d*_6_): *δ* (ppm) = 8.60 (s, 1H, N**H**–Ph), 7.37 (d, *J* = 11.7 Hz, 2H, Ar), 7.22 (m, *J* = 11.1 Hz, 2H, Ar), 6.89 (t, *J* = 10.9 Hz, 1H, Ar), 6.45 (d, *J* = 7.2 Hz, 1H, **NH**–CH), 4.50 (brs, 1H, COOH), 4.24–4.10 (m, 1H, **CH**), 1.30 (d, *J* = 6 Hz, 3H, **CH**_**3**_); ^13^C NMR (75 MHz, DMSO-*d*_6_): 174.9, 154.7, 140.2, 128.7 (2C), 121.3 (2C), 117.6, 48.0, 18.2; HRMS *m/z* calcd. for C_10_H_12_N_2_O_3_: 231.0746, found [M + H]^+^ 231.0741.

(*S*)-4-Amino-4-oxo-2-(3-phenylureido)butanoic acid (1b): white solid (567 mg, 5 mmol, 86%); *R*_f_ = 0.65 (90 : 10 EtOAc/MeOH); mp = 250 ± 2 °C; [α]_D_^20^ = +91.8 (*c* = 1, MeOH); ^1^H NMR (300 MHz, DMSO-*d*_6_): *δ* (ppm) = 8.89 (s, 1H, **NH**–Ph), 7.47–7.44 (m, 1H, Ar), 7.37 (d, *J* = 6.9 Hz, 2H, **NH**_**2**_), 7.25–7.18 (m, 2H, Ar), 7.00–6.91 (m, 1H, Ar), 6.91–6.86 (m, 1H, Ar), 6.50 (d, *J* = 8.4 Hz, 1H, **NH**), 4.44 (ddd, *J*_1_ = 13.2 Hz, *J*_2_ = 8.4 Hz, *J*_3_ = 4.8 Hz, 1H, **CH**), 2.68 (dd, *J*_1_ = 15.9 Hz, *J*_2_ = 5.7 Hz, 1H^a^, **H**_**2**_**C**), 2.53 (dd, *J* = 15.9 Hz, *J* = 5.1 Hz, 1H^b^, **CH**_**2**_); ^13^CNMR (75 MHz, DMSO-*d*_6_): 173.5, 171.8, 154.7, 140.4, 128.6, 128.7, 121.1, 117.4 (2C), 48.9, 37.1; HRMS *m*/*z* calcd. for C_11_H_14_N_3_O_4_: 252.0974, found [M + H]^+^ 252.0978.

(*S*)-4-Methyl-2-(3-phenylureido)pentanoic acid (1c): white solid (590 mg, 5 mmol, 90%); *R*_f_ = 0.69 (90 : 10 EtOAc/MeOH); mp = 160 ± 2 °C; [α]_D_^20^ = −6.2 (*c* = 1, MeOH); ^1^H NMR (300 MHz, DMSO-*d*_6_): *δ* (ppm) = 8.56 (s, 1H, **NH**–Ph), 7.37 (d, *J* = 11.7 Hz, 2H, Ar), 7.22 (t, *J* = 11.2 Hz, 2H, Ar), 6.89 (t, *J* = 10.6 Hz, 1H, Ar), 6.40 (d, *J* = 10.2 Hz, 1H, **NH**), 4.18 (dd, *J*_1_ = 21.3 Hz, *J*_2_ = 11.1 Hz, 1H, **HC**), 3.71 (brs, 1H, COOH), 1.84–1.60 (m, 1H, **H**C), 1.60–1.36 (m, 2H, **CH**_**2**_), 0.89 (t, *J* = 6.0 Hz, **2(CH**_**3**_**)**); ^13^C NMR (75 MHz, DMSO-*d*_6_): 175.0, 154.9, 140.3, 128.7 (2C), 121.7, 117.5 (2C), 50.8, 41.1, 24.4, 22.9, 21.7; HRMS *m*/*z* calcd. for C_13_H_18_N_2_O_3_: 273.1215, found [M + H]^+^ 273.1227.

(*S*)-3-Phenyl-2-(3-phenylureido)propanoic acid (1d): white solid (775 mg, 5 mmol, 94%); *R*_f_ = 0.63 (90 : 10 EtOAc/MeOH); mp = 198 ± 2 °C; [α]_D_^20^ = +74.3 (*c* = 1, MeOH); ^1^H NMR (300 MHz, DMSO-*d*_6_): *δ* (ppm) = 8.76 (s, 1H, **NH**–Ph), 7.38–7.16 (m, 9H, Ar), 6.89 (t, *J* = 11.1 Hz, 1H, Ar), 6.39 (d, *J* = 9 Hz, 1H, **NH**), 4.47–4.38 (m, 1H, **H**C), 3.82 (brs, 1H, COOH), 3.11 (dd, *J* = 13.8 Hz, *J* = 5.4 Hz, 1H^a^, **CH**_**2**_), 2.97 (dd, *J*_1_ = 13.8 Hz, *J*_2_ = 5.7 Hz, 1H^b^, **CH**_**2**_); ^13^C NMR (75 MHz, DMSO-*d*_6_): 173.6, 154.6, 140.2, 137.3, 129.3 (2C), 128.7 (2C), 128.2 (2C), 126.5, 121.1, 117.5 (2C), 53.6, 37.4; HRMS *m*/*z* calcd. for C_16_H_17_N_2_O_3_: 285.1239, found [M + H]^+^ 285.1243.

(*S*)-2-Phenyl-2-(3-phenylureido)acetic acid (1e): white solid (718 mg, 5 mmol, 95%); *R*_f_ = 0.59 (90 : 10 EtOAc/MeOH); mp = 194 ± 2 °C; [α]_D_^20^ = +55.9 (*c* = 1, MeOH); ^1^H NMR (300 MHz, DMSO-*d*_6_): *δ* (ppm) = 8.69 (s, 1H, **NH**–Ph), 7.41–7.33 (m, 8H, Ar), 7.22 (t, *J* = 7.8 Hz, 2H), 7.00 (d, *J* = 6 Hz, 1H, **NH**), 6.90 (t, *J* = 7.3 Hz, 1H), 5.24 (d, *J* = 7.9 Hz, 1H, **CH**); ^13^C NMR (75 MHz, DMSO-*d*_6_): 172.5, 154.3, 140.1, 138.2, 128.8 (2C), 128.7 (2C), 128.0, 127.1 (2C), 121.4, 117.6 (2C), 56.7; HRMS *m*/*z* calcd. for C_15_H_15_N_2_O_3_: 271.1083, found [M + H]^+^ 271.1081.

(*S*)-3-(3a,7a-Dihydro-1*H*-indol-3-yl)-2-(3-phenylureido)propanoic acid (1f): white solid (929 mg, 5 mmol, 91%); *R*_f_ = 0.61 (90 : 10 EtOAc/MeOH); mp = 162 ± 2 °C; [α]_D_^20^ = +243 (*c* = 1, MeOH); ^1^H NMR (300 MHz, DMSO-*d*_6_): *δ* (ppm) = 10.88 (s, 1H, COOH), 8.83 (s, 1H, **NH**–Ph), 7.55 (d, *J* = 8.1 Hz, 1H, Ar), 7.38–7.32 (m, 3H, Ar), 7.21 (t, *J* = 7.9 Hz, 2H, Ar), 7.15 (d, *J* = 2.4 Hz, 1H, Ar), 7.05 (t, *J* = 7.3 Hz, 1H, Ar), 7.03–6.84 (m, 2H, Ar), 6.42 (d, *J* = 7.8 Hz, 1H, **NH**), 4.47 (m, 1H, **HC**), 3.23 (dd, *J*_1_ = 12 Hz, *J*_2_ = 3 Hz, 1H^a^, **HC**), 3.13 (dd, *J*_1_ = 14.7 Hz, *J*_2_ = 6.3 Hz, 1H^b^, **HC**); ^13^C NMR (75 MHz, DMSO-*d*_6_): 174.2, 154.8, 140.5, 136.1, 128.6 (2C), 127.6, 123.7, 121.0, 120.8, 118.4, 118.3, 118.1, 117.5 (2C), 111.2, 109.7, 53.4, 27.7; HRMS *m*/*z* calcd. for C_18_H_18_N_3_O_3_: 324.1348, found [M + H]^+^ 324.1348.

(*S*)-3-Methyl-2-(3-phenylureido)butanoic acid (1g): white solid (515 mg, 5 mmol, 88%); *R*_f_ = 0.66 (90 : 10 EtOAc/MeOH); mp = 146 ± 2 °C; [α]_D_^20^ = −15,3 (*c* = 1, MeOH); ^1^H NMR (300 MHz, DMSO-*d*_6_): *δ* (ppm) = 8.61 (s, 1H, **NH**–Ph), 7.37 (t, *J* = 7.8 Hz, 2H, Ar), 7.22 (t, *J* = 7.8 Hz, 2H, Ar), 6.90 (t, *J* = 7.3 Hz, 1H, Ar), 6.39 (d, *J* = 8.7 Hz, 1H, **NH**), 4,12 (dd, *J*_1_ = 8.7 Hz, *J*_2_ = 4.8 Hz, 1H, **CH**), 2.16–2.01 (m, 1H, **H**C), 0.92 (d, *J* = 6.9 Hz, 3H, **CH**_**3**_), 0.87 (d, *J* = 6.9 Hz, 3H, **CH**_**3**_); ^13^C NMR (75 MHz, DMSO-*d*_6_): 173.8, 155.0, 140.2, 128.7 (2C), 121.1, 117.4 (2C), 57.1, 30.2, 19.2, 17.5; HRMS *m*/*z* calcd. for C_12_H_17_N_2_O_3_: 237.1239, found [M + H]^+^ 237.1233.

(*S*)-4-(Methylthio)-2-(3-phenylureido)butanoic acid (1h): according to the general procedure, the product was isolated as white solid (671 mg, 5 mmol, 90%); *R*_f_ = 0.62 (90 : 10 EtOAc/MeOH); mp = 120 ± 2 °C; ^1^H NMR (300 MHz, DMSO-*d*_6_): *δ* (ppm) = 8.64 (s, 1H, **NH**–Ph), 7.37 (d, *J* = 7.5 Hz, 2H, Ar), 7.21 (t, *J* = 7.9 Hz, 2H, Ar), 6.88 (t, *J* = 7.3 Hz, 1H, Ar), 6.52 (d, *J* = 7.8 Hz, 1H, **NH**), 4.27 (ddd, *J*_1_ = 15 Hz, *J*_2_ = 6 Hz; *J*_3_ = 3 Hz, 1H, **H**C), 2.54–2.45 (m, 2H, SCH_2_), 2.04 (s, 3H, **CH**_**3**_), 2.02–1.93 (m, 1H, **CH**_**2**_), 1.92–1.78 (m, 1H, **CH**_**2**_); ^13^C NMR (75 MHz, DMSO-*d*_6_): 174.1, 154.9, 140.2, 128.7 (2C), 121.3, 117.7 (2C), 51.6, 31.7, 29.6, 14.7; HRMS *m*/*z* calcd. for C_12_H_17_N_2_O_3_S: 269.0960, found [M + H]^+^ 269.0956.

(*S*)-2-(3-(Naphthalen-2-yl)ureido)propanoic acid (2a): white solid (432 mg, 5 mmol, 97%); *R*_f_ = 0.67 (90 : 10 EtOAc/MeOH); mp = 220 ± 2 °C; [α]_D_^20^ = +26.7 (*c* = 1, MeOH); 1H NMR (300 MHz, DMSO-*d*_6_): *δ* (ppm) = 12.51 (s, 1H, COOH), 8.70 (s, 1H, **NH**–naph), 8.12 (d, *J* = 7.8 Hz, 1H, Ar), 8.02 (d, *J* = 7.2 Hz, 1H, Ar), 7.93–7.86 (m, 1H, Ar), 7.60–7.47 (m, 3H, Ar), 7.42 (t, *J* = 7.9 Hz, 1H, Ar), 6.99 (d, *J* = 7.5 Hz, 1H, **NH**), 4.30–4.21 (m, 1H, C**H**), 1.36 (d, *J* = 7.2 Hz, 3H, **CH**_**3**_); ^13^C NMR (75 MHz, DMSO-*d*_6_): 174.9, 155.0, 134.9, 133.7, 128.4, 125.9, 125.8, 125.5, 125.3, 122.1, 121.3, 116.2, 48.2, 18.4; HRMS *m*/*z* calcd. for C_14_H_15_N_2_O_3_: 259.1083, found [M + H]^+^ 259.1083.

(*S*)-4-Amino-2-(3-(naphthalen-2-yl)ureido)-4-oxobutanoic acid (2b): white solid (560 mg, 5 mmol, 85%); *R*_f_ = 0.68 (90 : 10 EtOAc/MeOH); mp = 250 ± 2 °C; [α]_D_^20^ = +320 (*c* = 1, MeOH); ^1^H NMR (300 MHz, DMSO-*d*_6_): *δ* (ppm) = 8.92 (s, 1H, **NH**–naph), 8.20–8.15 (m, 1H, Ar), 8.03 (d, *J* = 6.9 Hz, 1H, Ar), 7.91–7.85 (m, 1H, Ar), 7.56–7.47 (m, 3H, Ar), 7.41 (t, *J* = 7.8 Hz, 1H, Ar), 7.10 (d, *J* = 8.1 Hz, 1H, **NH**), 6.97 (s, 2H, **NH**_**2**_), 4.47–4.54 (m, 1H), 3.53 (brs, 1H, COOH), 2.71 (dd, *J*_1_ = 15.9 Hz, *J*_2_ = 5.7 Hz, 1H^a^, **HC**), 2.60 (dd, *J*_1_ = 15.9 Hz, *J*_2_ = 5.1 Hz, 1H^b^, **HC**); ^13^C NMR (75 MHz, DMSO-*d*_6_): 173.6, 171.8, 155.3, 135.1, 133.7, 128.3, 125.9, 125.7, 125.4, 125.3, 122.1, 121.5, 116.3, 49.3, 37.5; HRMS *m*/*z* calcd. for C_15_H_16_N_3_O_4_: 302.1141, found [M + H]^+^ 302.1136.

(*S*)-4-Methyl-2-(3-(naphthalen-2-yl)ureido)pentanoic acid (2c): white solid (583 mg, 5 mmol, 89%); *R*_f_ = 0.68 (90 : 10 EtOAc/MeOH); mp = 178 ± 2 °C; [α]_D_^20^ = +18.2 (*c* = 1, MeOH); ^1^H NMR (300 MHz, DMSO-*d*_6_): *δ* (ppm) = 8.67 (s, 1H, **NH**–naph), 8.11 (d, *J* = 8.1 Hz, 1H, Ar), 8.03 (d, *J* = 7.5 Hz, 1H, Ar), 7.89 (d, *J* = 9.3 Hz, 1H, Ar), 7.59–7.48 (m, 3H, Ar), 7.41 (t, *J* = 7.9 Hz, 1H, Ar), 6.93 (d, *J* = 8.4 Hz, 1H, **NH**), 4.22–4.31 (m, 1H), 1.84–1.69 (m, 1H, **H**C), 1.65–1.49 (m, 2H, **CH**_**2**_), 0.95 (d, *J* = 6.6 Hz, 3H, **CH**_**3**_), 0.92 (d, *J* = 6.6 Hz, 3H, **CH**_**3**_); ^13^C NMR (75 MHz, DMSO-*d*_6_): 174.9, 155.2, 134.9, 133.7, 128.4, 125.9, 125.8, 125.5, 125.2, 122.1, 121.2, 116.1, 50.9, 41.1, 24.5, 22.9, 21.7; HRMS *m*/*z* calcd. for C_17_H_21_N_2_O_3_: 301.1552, found [M + H]^+^ 301.1560.

(*S*)-2-(3-(Naphthalen-2-yl)ureido)-3-phenylpropanoic acid (2d): white solid (759 mg, 5 mmol, 92%); *R*_f_ = 0.64 (90 : 10 EtOAc/MeOH); mp = 218 ± 2 °C; [α]_D_^20^ = +48.9 (*c* = 1, MeOH); ^1^H NMR (300 MHz, DMSO-*d*_6_): *δ* (ppm) = 8.76 (s, 1H, **NH**–naph), 8.08 (d, *J* = 9.0 Hz, 1H, Ar), 7.97 (d, *J* = 7.5 Hz, 1H, Ar), 7.90–7.87 (m, 1H, Ar), 7.61–7.46 (m, 3H, Ar), 7.41 (t, *J* = 7.8 Hz, 1H, Ar), 7.36–7.19 (m, 5H, Ar), 6.92 (d, *J* = 7.8 Hz, 1H, **NH**), 4.58–4.51 (m, 1H, **H**C), 3.36 (brs, 1H, COOH), 3.14 (dd, *J*_1_ = 13.8 Hz, *J*_2_ = 5.1 Hz, 1H^a^, **CH**_**2**_), 2.99 (dd, *J*_1_ = 13.5 Hz, *J*_2_ = 7.2 Hz, 1H^b^, **CH**_**2**_); ^13^C NMR (75 MHz, DMSO-*d*_6_): 173.4, 155.0, 137.2, 134.9, 133.7, 129.3 (2C), 128.3, 128.2 (2C), 126.5, 125.9, 125.7, 125.4, 122.2, 121.4, 116.4, 53.7, 37.5; HRMS *m*/*z* calcd. for C_20_H_19_N_2_O_3_: 335.1396, found [M + H]^+^ 335.1398.

(*S*)-2-(3-(Naphthalen-1-yl)ureido)-2-phenylglycine (2e): white solid (649 mg, 5 mmol, 86%); *R*_f_ = 0.60 (90 : 10 EtOAc/MeOH); mp = 230 ± 2 °C; [α]_D_^20^ = +347 (*c* = 1, MeOH); ^1^H NMR (300 MHz, DMSO-*d*_6_): *δ* (ppm) = 8.83 (s, 1H, **NH**–naph), 8.13 (d, *J* = 6.9 Hz, 1H, **NH**), 8.06–8.03 (m, 1H, Ar), 7.91–7.87 (m, 1H, Ar), 7.63–7.49 (m, 10H, Ar), 5.33 (d, *J* = 7.2 Hz, 1H, **CH**); ^13^C NMR (75 MHz, DMSO-*d*_6_): 172.5, 154.6, 138.2, 134.8, 133.7, 128.7 (2C), 128.4, 128.0, 127.2, 127.1, 125.9, 125.8, 125.5, 125.1, 122.1, 121.1, 115.8, 56.9; HRMS *m*/*z* calcd. for C_19_H_17_N_2_O_3_: 321.1239, found [M + H]^+^ 321.1231.

(*S*)-3-(1H-indol-3-yl)-2-(3-(naphthalen-2-yl)ureido)propanoic acid (2f): white solid (948 mg, 5 mmol, 93%); *R*_f_ = 0.58 (90 : 10 EtOAc/MeOH); mp = 190 ± 2 °C; [α]_D_^20^ = +30.1 (*c* = 1, MeOH); ^1^H NMR (300 MHz, DMSO-*d*_6_): *δ* (ppm) = 10.93 (s, 1H, COOH), 8.77 (s, 1H, **NH**–naph), 8.12–8.05 (m, 1H, Ar), 8.00 (d, *J* = 7.2 Hz, 1H, Ar), 7.92–7.85 (m, 1H, Ar), 7.61–7.47 (m, 4H, Ar), 7.41 (t, *J* = 7.8 Hz, 1H, Ar), 7.36 (d, *J* = 7.8 Hz, 1H, Ar), 7.19 (d, *J* = 2.1 Hz, 1H, Ar), 7.07 (t, *J* = 7.2 Hz, 1H, Ar), 7.01–6.88 (m, 2H, Ar and **NH**), 4.59 (dd, *J*_1_ = 13.4 Hz, *J*_2_ = 6.3 Hz, 1H, **HC**), 3.27 (dd, *J*_1_ = 12 Hz, *J*_2_ = 3 Hz, 1H^a^, **CH**_**2**_), 3.17 (dd, *J*_1_ = 15 Hz, *J*_2_ = 6.3 Hz, 1H^b^, **CH**_**2**_); ^13^C NMR (75 MHz, DMSO-*d*_6_): 173.9, 155.1, 136.1, 134.9, 133.7, 128.4, 127.5, 125.9, 125.8, 125.5, 125.4, 123.8, 122.2, 121.4, 120.9, 118.4 (2C), 116.5, 111.4, 109.4, 53.4, 27.8; HRMS *m*/*z* calcd. for C_22_H_20_N_3_O_3_: 374.1505, found [M + H]^+^ 374.1507.

(*S*)-3-Methyl-2-(3-(naphthalen-2-yl)ureido)butanoic acid (2g): white solid (509 mg, 5 mmol, 87%); *R*_f_ = 0.63 (90 : 10 EtOAc/MeOH); mp = 210 ± 2 °C; [α]_D_^20^ = +229 (*c* = 1, MeOH); ^1^H NMR (300 MHz, DMSO-*d*_6_): *δ* (ppm) = 8.74 (s, 1H, **NH**–naph), 8.13 (d, *J* = 8.1 Hz, 1H, Ar), 8.05 (dd, *J*_1_ = 7.6 Hz, *J*_2_ = 0.6 Hz, 1H, Ar), 7.91–7.88 (m, 1H, Ar), 7.60–7.48 (m, 3H, Ar), 7.41 (t, *J* = 7.8 Hz, 1H, Ar), 6.96 (d, *J* = 8.7 Hz, 1H, **NH**), 4.20 (dd, *J*_1_ = 8.7 Hz, *J*_2_ = 4.5 Hz, 1H), 2.19–2.08 (m, 1H, **H**C), 0.97 (d, *J* = 6.9 Hz, 3H, **CH**_**3**_), 0.93 (d, *J* = 6.9 Hz, 3H, **CH**_**3**_); ^13^C NMR (75 MHz, DMSO-*d*_6_): 173.7, 155.4, 134.9, 133.7, 128.4, 125.9, 125.8, 125.4, 125.1, 121.9, 121.1, 115.8, 57.4, 30.4, 19.2, 17.6; HRMS *m*/*z* calcd. for C_16_H_19_N_2_O_3_: 287.1396, found [M + H]^+^ 287.1409.

### Pharmacology

#### Animals

Wistar rats of either sex, weighing 200–220 g, were obtained from Pasteur Institute (Tunis, Tunisia). They were kept in groups of 6 animals in stainless-steel cages at 20–25 °C and maintained on a standard pellet diet with free access to water. All animals were treated according to the guidelines established by the European Union regarding the Use and the Animal Care (CCE Council 86/609).

#### Anti-inflammatory activity

The anti-inflammatory activity of compounds 1a, 1e, 1f, 2f on carrageenan-induced rat paw edema was determined by the method of Winter *et al.* (1962).^43^ Rats were divided into groups of 6 animals each *n* = 6. The control group received 2.5 mL kg^−1^ of vehicle solution (tween 80/absolute ethanol/saline solution (0.9%) in the ratio 1 : 1 : 18) by the intraperitoneal (i.p.) route. The reference group received diclofenac which is a non-steroidal anti-inflammatory drug (25 mg kg^−1^, i.p.) and the test groups received compounds 1a, 1f, 2f at increasing doses (25 and 50 mg kg^−1^, i.p) and 1e (25, 12.5 mg kg^−1^ i.p.). After 30 minutes, 0.05 mL of a 1% carrageenan suspension was administered by subplantar injection into the left hind paw. The paw volume up to the tibiotarsal articulation was measured using a plethysmometer (Ugo Basile no.7140, Italy) immediately before carrageenan injection (V0) and then 1, 2, 3, 4 and 5 h post carrageenan injection (VT). The percentage inhibition in the increase of paw volume for each rat was calculated using the formula given below:[(VT − V0) control − (VT − V0) treated] × 100/(VT − V0) control.

#### Statements for the animal experiments

All animal procedures were performed according to the guidelines established by the European Union regarding the Use and the Animal Care (CCE Council 86/609) and with the approval of the ethic committee on the research in life sciences and health of the Higher Institute of Biotechnology of Monastir (University of Monastir, Tunisia).

#### Molecular docking procedure

Molecular docking studies were carried out using the AutoDock 4.2 software package.^48^ All the geometries of the compounds were built and optimized with ACD (3D Viewer) software accessed on July 10, 2022 by http://www.filefacts.com/acd3d-viewer-freeware-info. The crystal structures of COX-1(PDB: 6Y3C) and COX-2 (PDB: 5KIR) were obtained from the RCSB Protein Data Bank.^49^ Initially, water molecules were removed from the system, and the missing hydrogens and Gasteiger charges were added during the preparation of the receptor input file. Then, AutoDock Tools were employed to prepare the ligand and protein files (in PDBQT format). The grid maps were done using AutoGrid to expedite the docking process. The docking calculation was then performed with a grid map of 40 × 40 × 40 Å points and a grid-point spacing of 0.375 Å, centered on the receptor in order to determine the active site. Finally, interactions were visualized and analyzed using the software Discovery Studio 2017R2 (https://www.3dsbiovia.com/products/collaborative-science/biovia-discovery-studio/).

#### Statements for the animal experiments

All animal procedures were performed according to the guidelines established by the European Union regarding the Use and the Animal Care (CCE Council 86/609) and with the approval of the ethic committee on the research in life sciences and health of the Higher Institute of Biotechnology of Monastir (University of Monastir, Tunisia).

#### ADME properties

The pharmacokinetic, toxicity, and drug-likeness properties of the predominant constituents were assessed using the pkCSM server https://biosig.lab.uq.edu.au/pkcsm/, while mutagenicity was investigated with the VEGA https://www.vegahub.eu/ platform through a QSAR model evaluated within its applicability domain.

## Conclusions

In summary, this study describes an efficient and environmentally friendly method for synthesizing a novel series of chiral aryl ureido amino acid derivatives from the chiral pool, using water as a green solvent. The process demonstrates outstanding sustainability metrics, including a perfect atom economy (AE = 100%) and an excellent E-factor (0.15–0.25). The *in silico* study was expanded to include molecular docking to evaluate the binding affinity of the synthesized compounds for cyclooxygenase (COX) isoenzymes, thus facilitating the pre-selection of compounds for *in vivo* testing. Significant results were obtained, and some of these compounds, such as 1a, 1e, 1f and 2f, showed interesting binding energies and types of interactions compared to the diclofenac that was employed as a reference drug. These compounds were evaluated for their anti-inflammatory activity. The *in vivo* results were consistent, particularly for compound 1e, which exhibited the highest activity (edema inhibition = 97.05%) compared to diclofenac (edema inhibition = 63.82%). Based on these results, compound 1e can be considered a promising lead that merits further investigation for the development of more potent and safer anti-inflammatory agents. Overall, the candidate compounds exhibited favorable ADMET profiles. Among them, 1e and 2f demonstrated the most balanced safety and ADME characteristics, combining non-mutagenicity, acceptable toxicity margins, and favorable drug-likeness, highlighting their potential as lead compounds in drug discovery.

## Conflicts of interest

There are no conflicts to declare.

## Supplementary Material

RA-015-D5RA04473A-s001

## Data Availability

The data supporting this article have been included as part of the supplementary information (SI). Supplementary information: copies of NMR spectra and 2D diagram of the interactions resulting from molecular docking. See DOI: https://doi.org/10.1039/d5ra04473a.
