# Supplementary material for: Green synthesis of new chiral amino acid urea derivatives and evaluation of their anti-inflammatory activity
Source: RSC Adv. 2025 Sep 30;15(43):36219–29. doi: 10.1039/d5ra04473a (PMC12481247; doi:10.1039/d5ra04473a)
Supplement: RA-015-D5RA04473A-s001 [file RA-015-D5RA04473A-s001.pdf]

Supplementary Information (SI) for RSC Advances.
This journal is © The Royal Society of Chemistry 2025

Supplementary Material (ESI) for RSC Advances
This journal is (c) The Royal Society of Chemistry 2025

Green synthesis of new chiral amino acid urea derivatives and evaluation of their anti-inflammatory activity

Sirine Mehrez[a], Sarah Absi[a], Assia Hamdi[a], Sylvain Marque*[b], Abderrahman Bouraoui[a], Yakdhane Kacem*[a], Jamil Kraiem*[a]

[a] Laboratoire de Développement Chimique, Galénique et Pharmacologique des Médicaments, Faculté de Pharmacie de Monastir, Université de Monastir, Rue Avicenne, 5000 Monastir, Tunisie.
[b] Université Paris-Saclay, UVSQ, CNRS, UMR 8180, Institut Lavoisier de Versailles (ILV), 45 avenue des Etats-Unis, 78 035 Versailles Cedex, France. E-mail: sylvain.marque@uvsq.fr

SUPPORTING INFORMATION

Table of contents

Part 1: Spectroscopies......S1
1. Labels of **1a-h** and **2a-g** for spectrum section and characterization......S2
2. NMR spectra of **1a**......S3
3. NMR spectra of **1b**......S6
4. NMR spectra of **1c**......S9
5. NMR spectra of **1d**......S12
6. NMR spectra of **1e**......S16
7. NMR spectra of **1f**......S19
8. NMR spectra of **1g**......S24
9. NMR spectra of **1h**......S26
10. NMR spectra of **2a**......S30
11. NMR spectra of **2b**......S35
12. NMR spectra of **2c**......S38
13. NMR spectra of **2d**......S41
14. NMR spectra of **2e**......S45
15. NMR spectra of **2f**......S49
16. NMR spectra of **2g**......S55

Part 2: Molecular docking......S60
1. The corresponding 2D diagram of the interactions of the compounds **1a-h**, **2a-g** and diclofenac at the active site of COX-1......S60
2. The corresponding 2D diagram of the interactions of the compounds **1a-h**, **2a-g** and diclofenac at the active site of COX-2......S63

Part 1: Spectroscopies

1. Labels of 1a-h and 2a-g for spectrum section and characterization

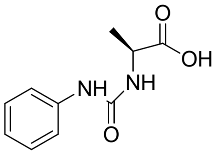

1a

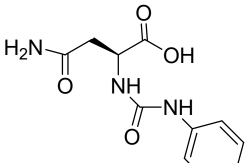

1b

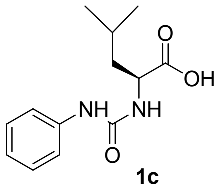

1c

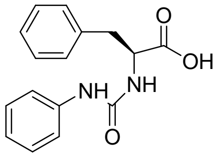

1d

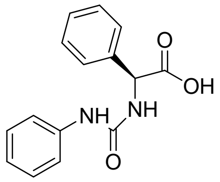

1e

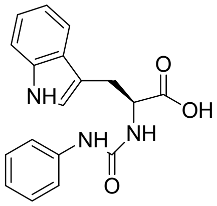

1f

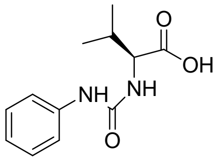

1g

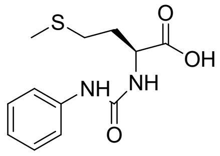

1h

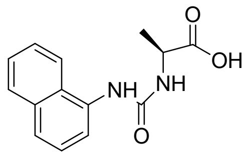

2a

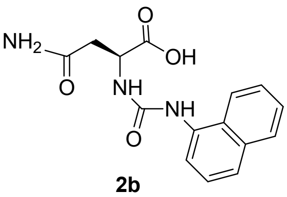

2b

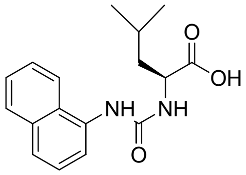

2c

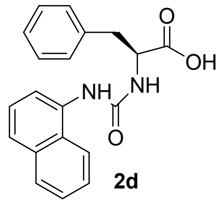

2d

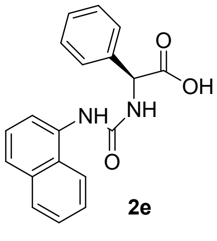

2e

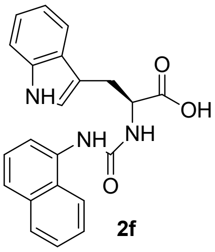

2f

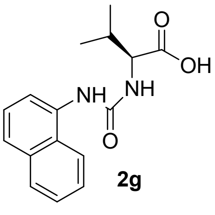

2g

2. NMR spectra of 1a

^{1}H NMR (DMSO-d_6) of **1a** at 300 MHz

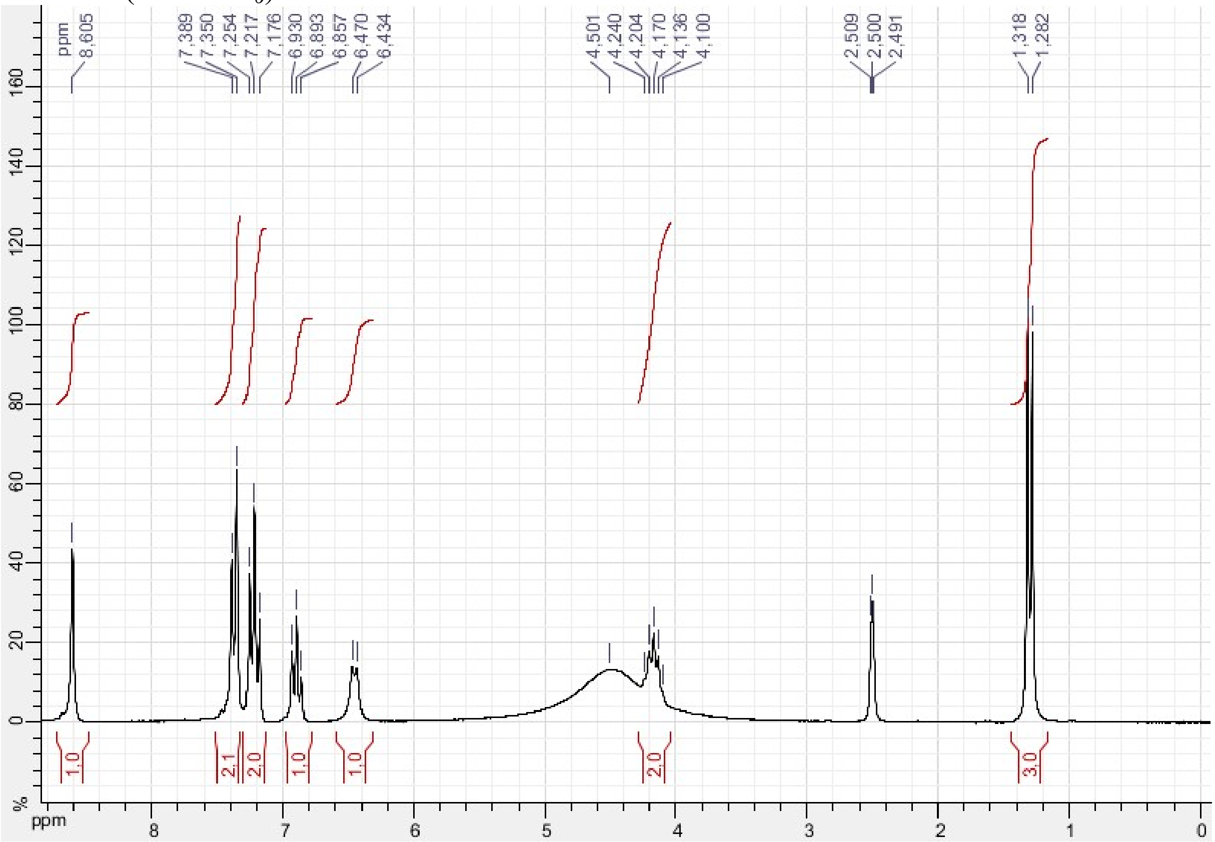

^{13}C NMR (DMSO-d_6) of **1a** at 75 MHz

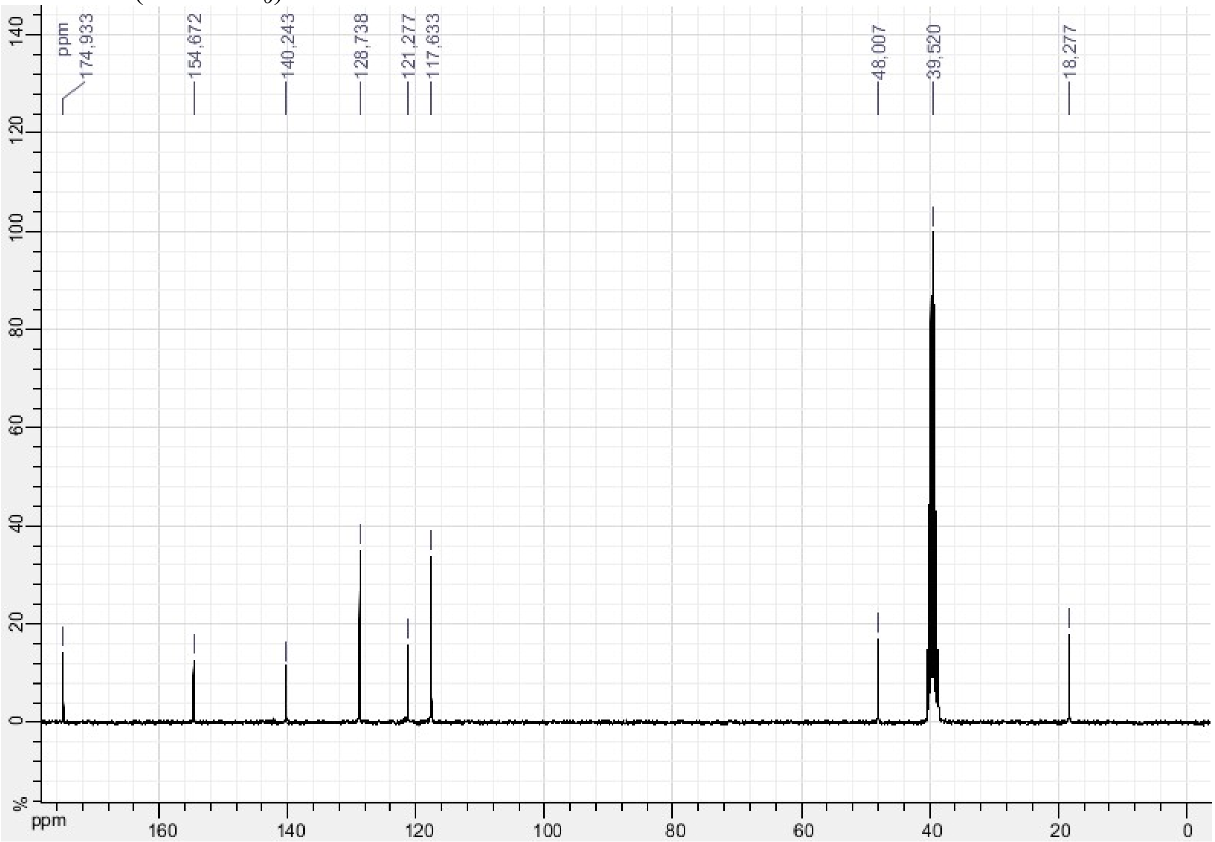

^{13}C (Dept135) NMR spectrum of **1a** in DMSO-d_6 at 75 MHz

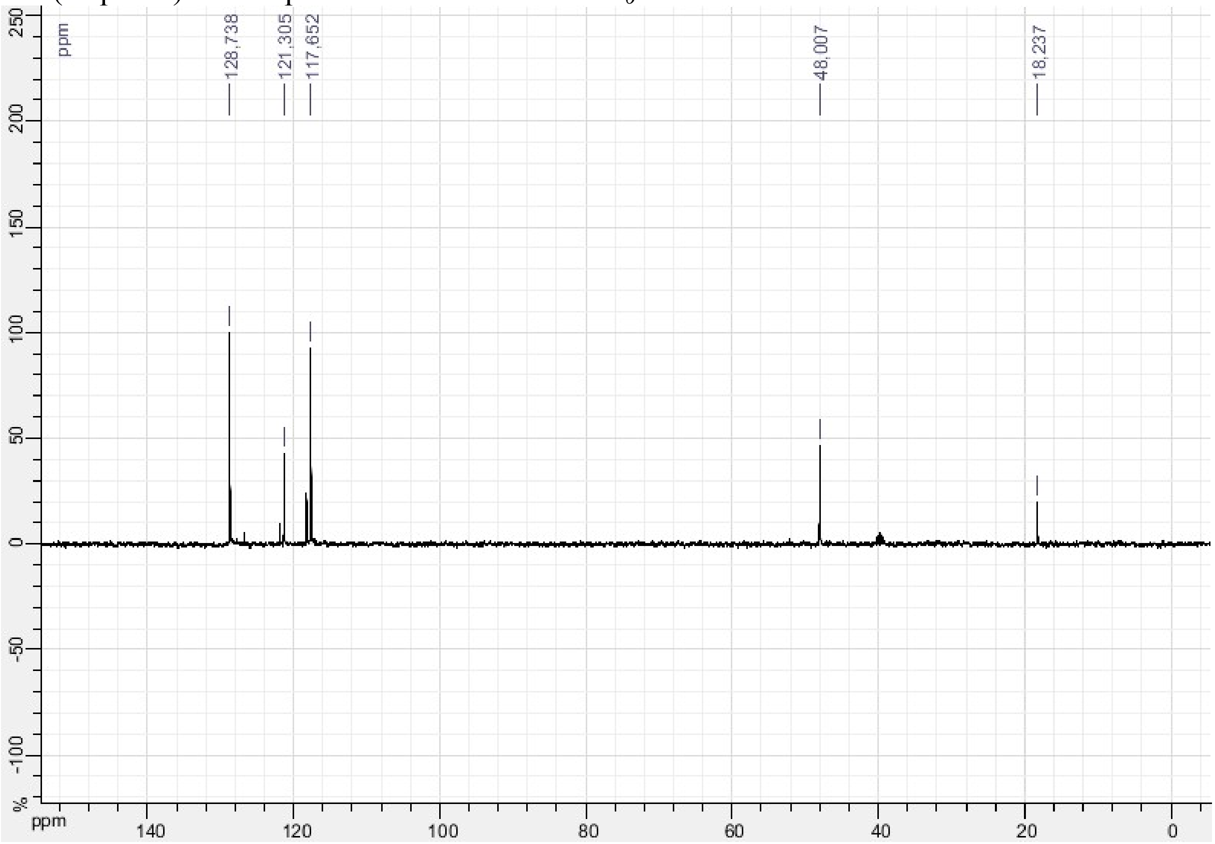

COSY NMR spectrum of **1a** in DMSO-d_6

Cosy NMR spectrum of **1a** in DMSO-d_6

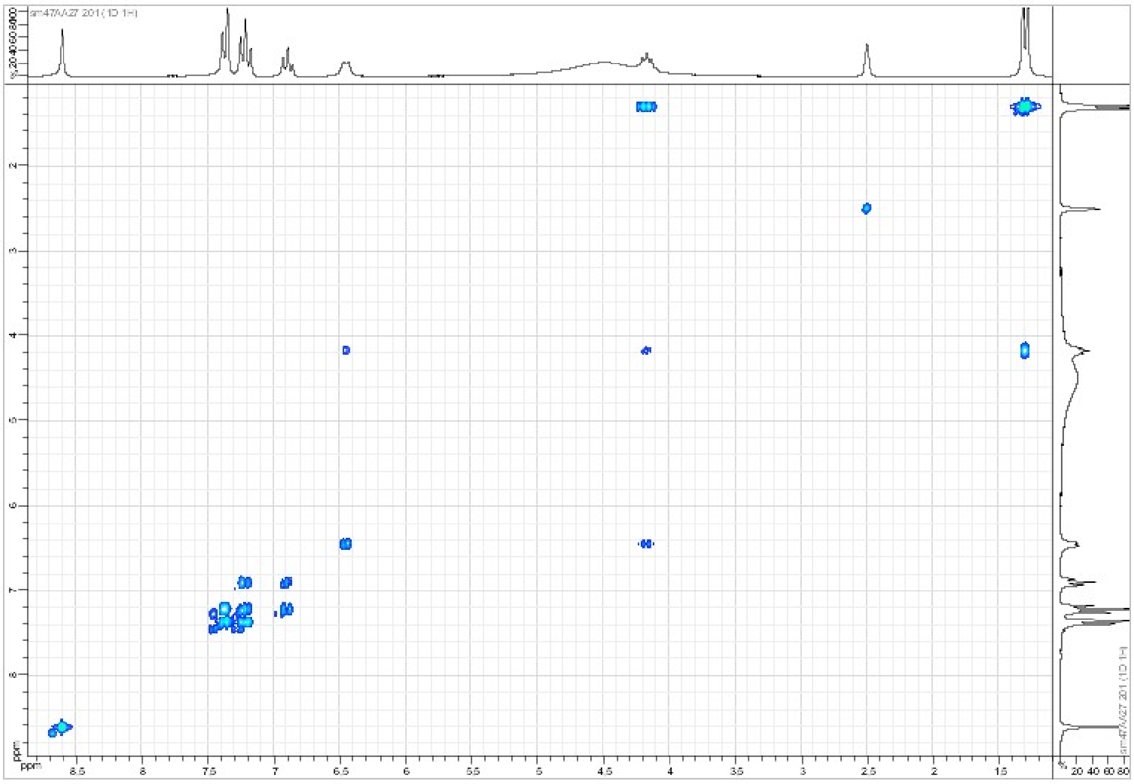

HSQC NMR spectrum of **1a** in DMSO-d_6

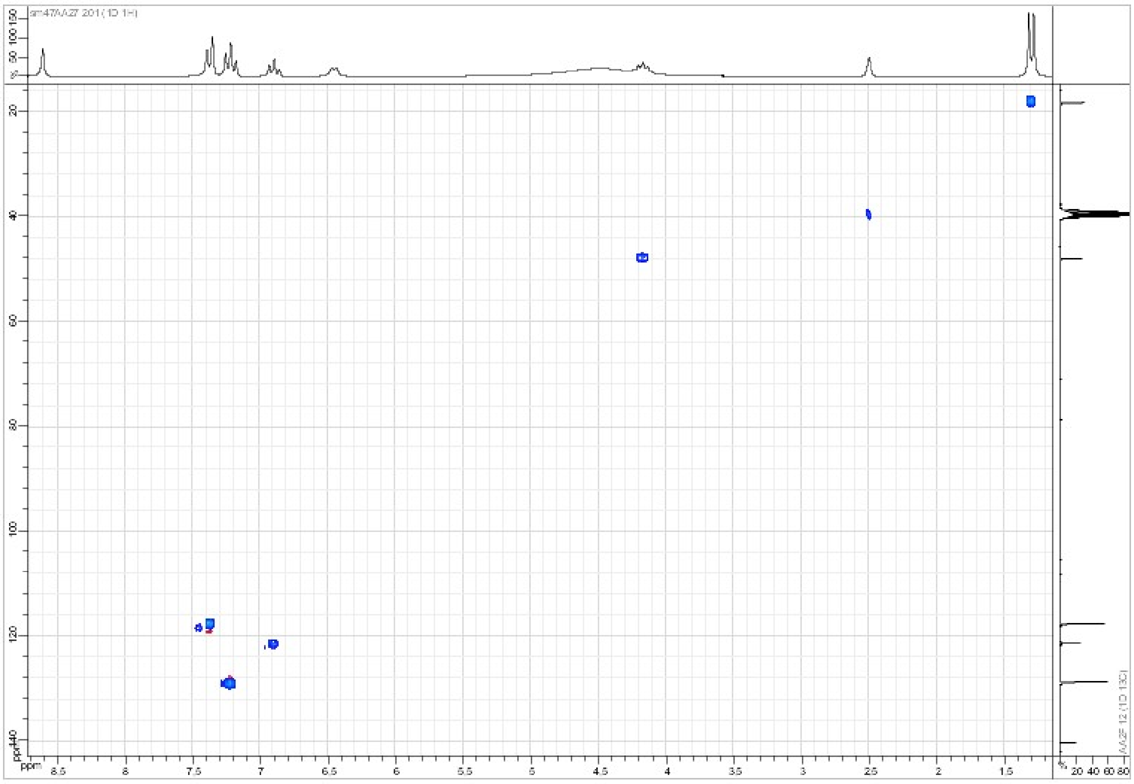

HMBC NMR spectrum of **1a** in DMSO-d_6

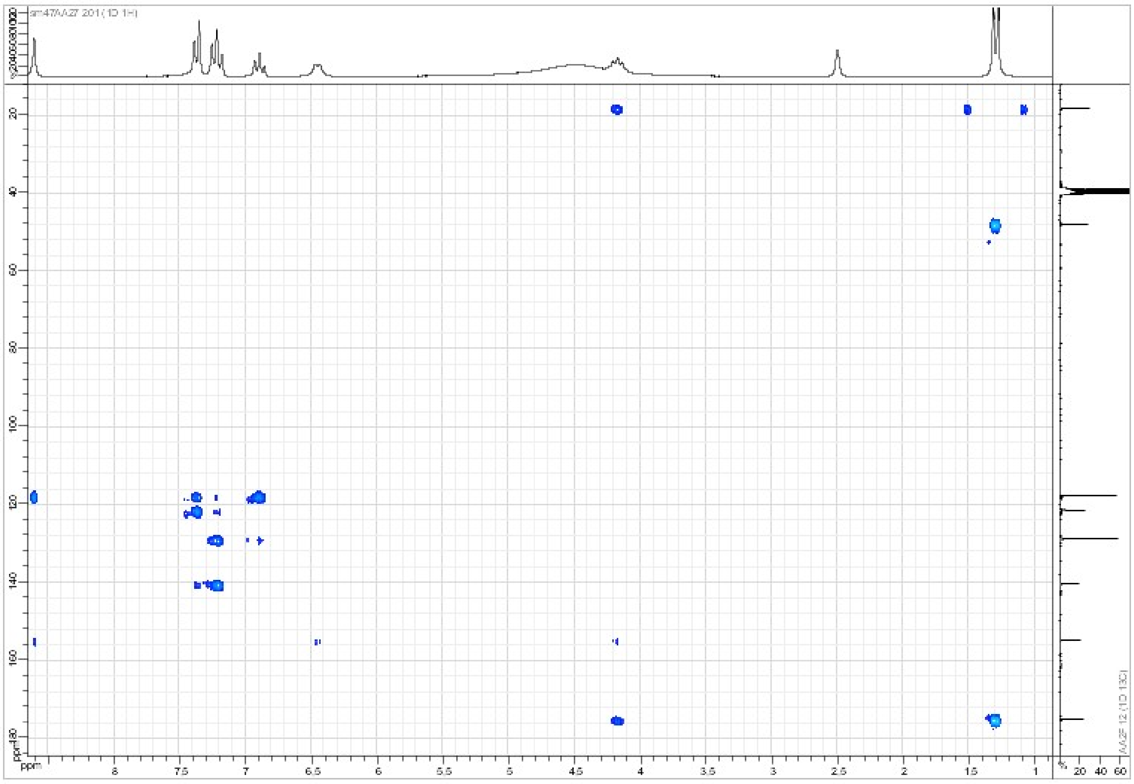

3. NMR spectra of 1b

^{1}H NMR (DMSO-d_6) of **1b** at 300 MHz

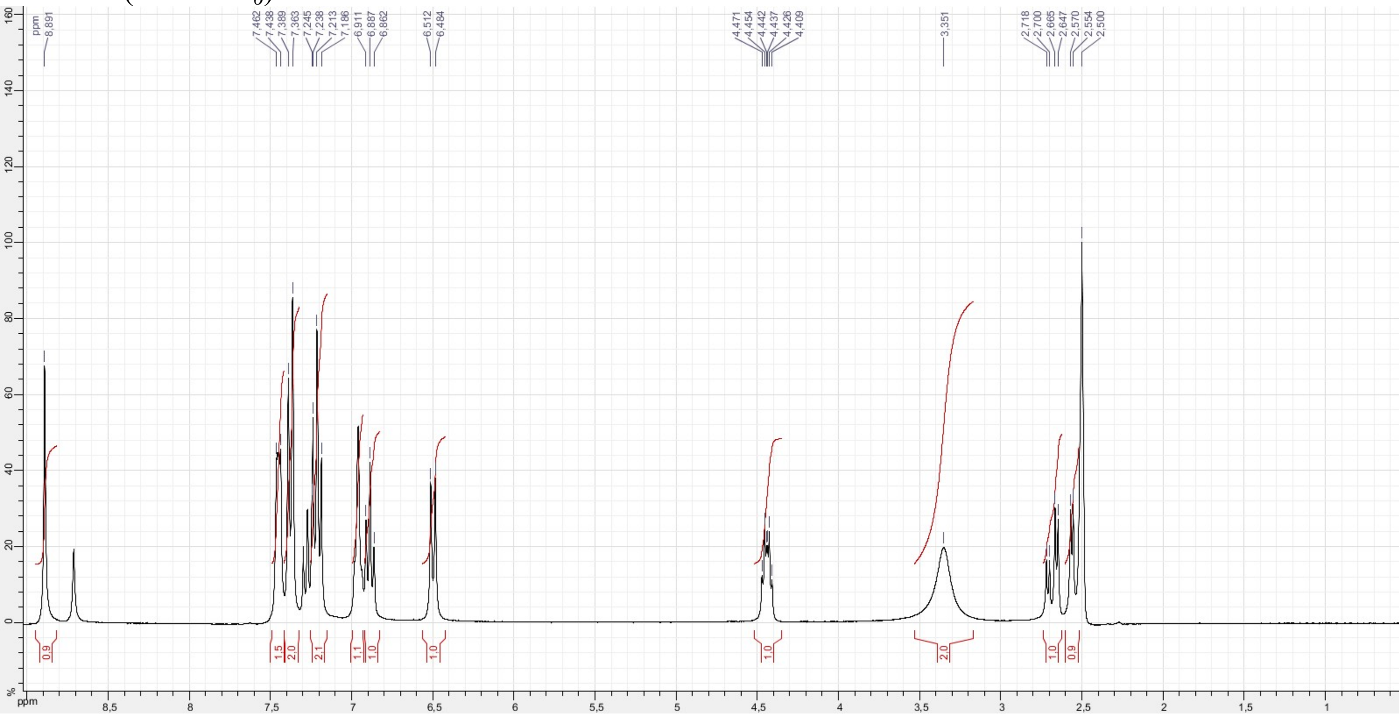

^{13}C NMR (DMSO-d_6) of **1b** at 75 MHz

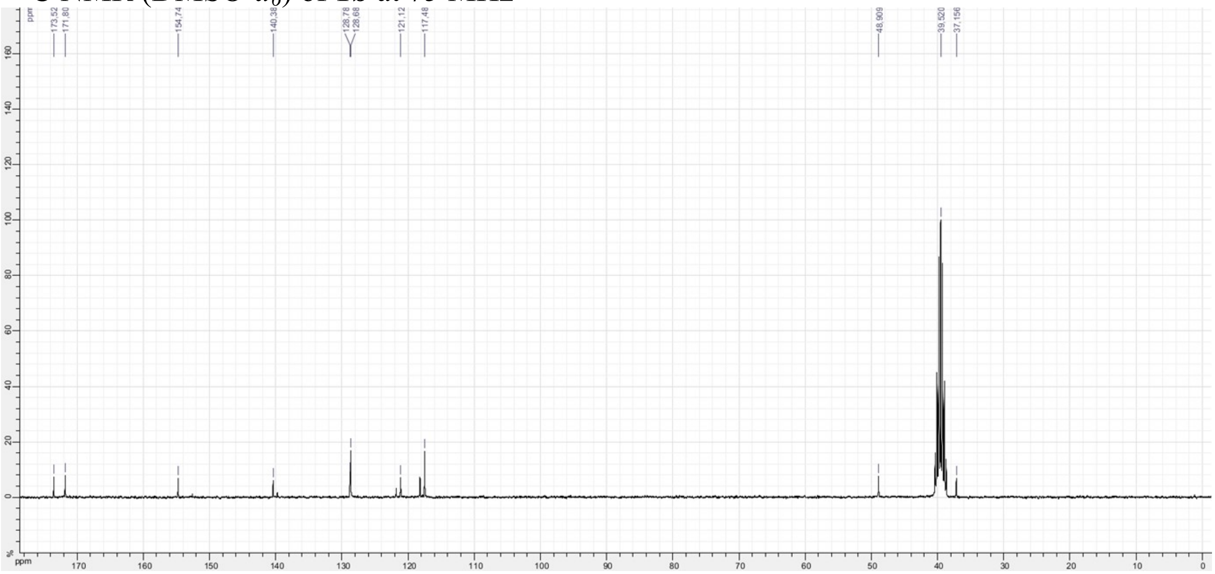

^{13}C (Dept135) NMR spectrum of **1b** in DMSO-d_6 at 75 MHz

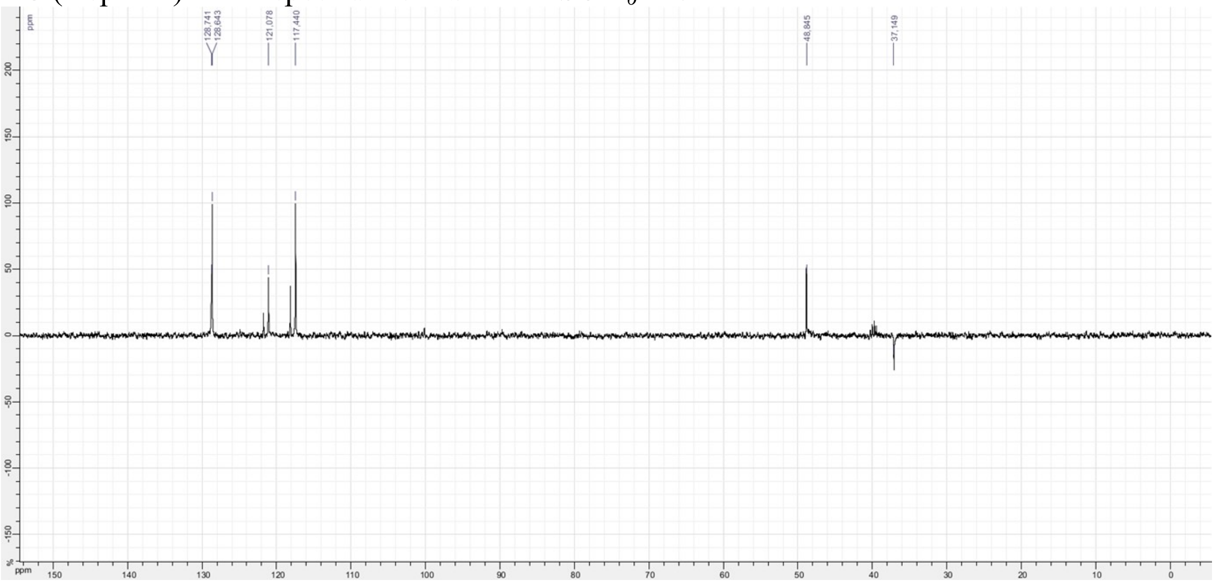

COSY NMR spectrum of **1b** in DMSO-d_6

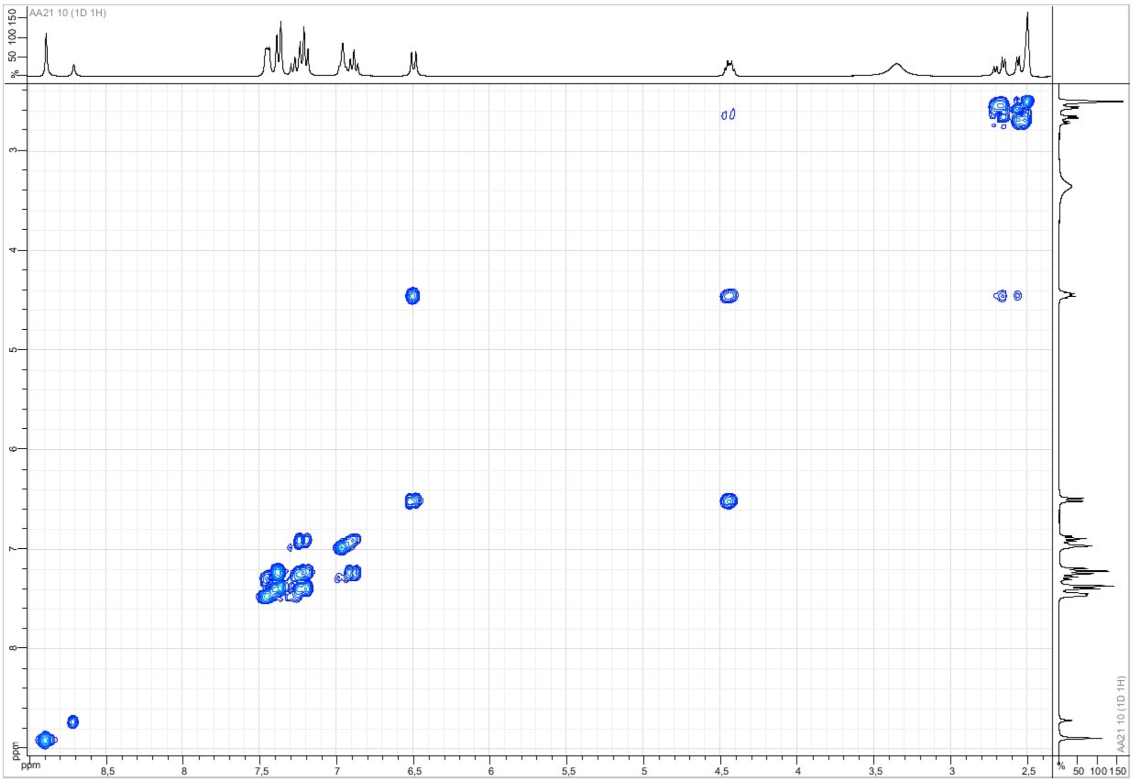

HSQC NMR spectrum of **1b** in DMSO-d_6

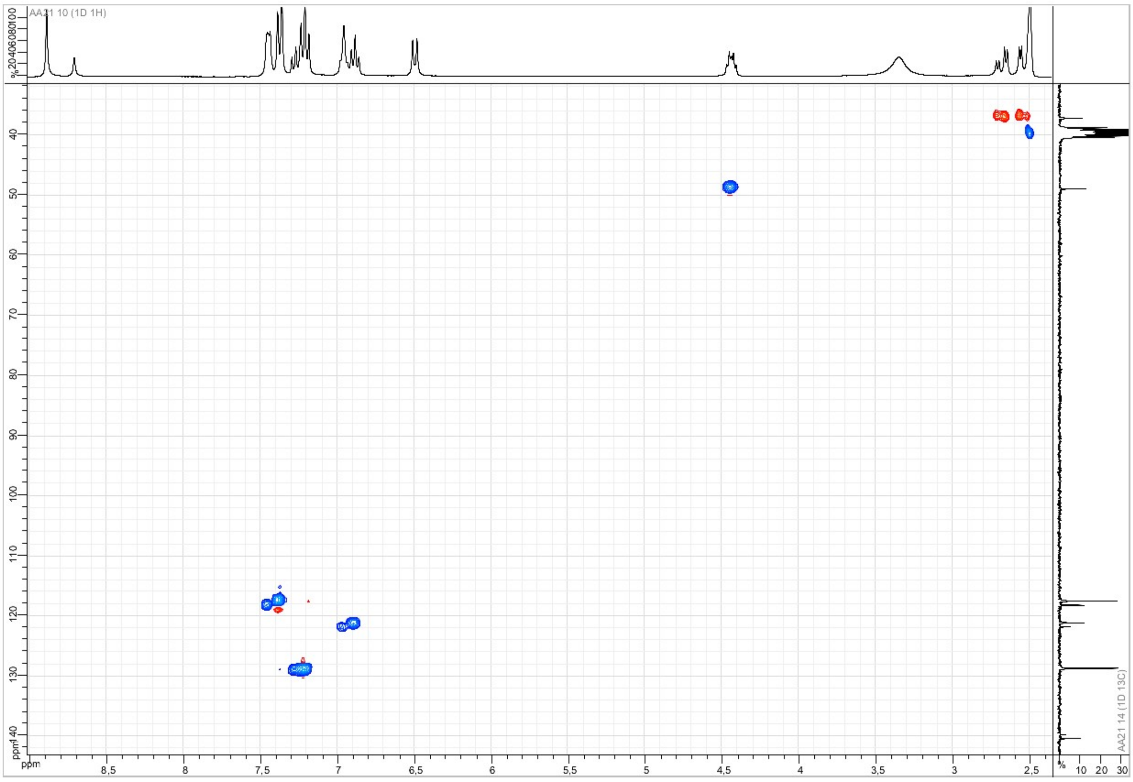

HMBC NMR spectrum of **1b** in DMSO-d_6

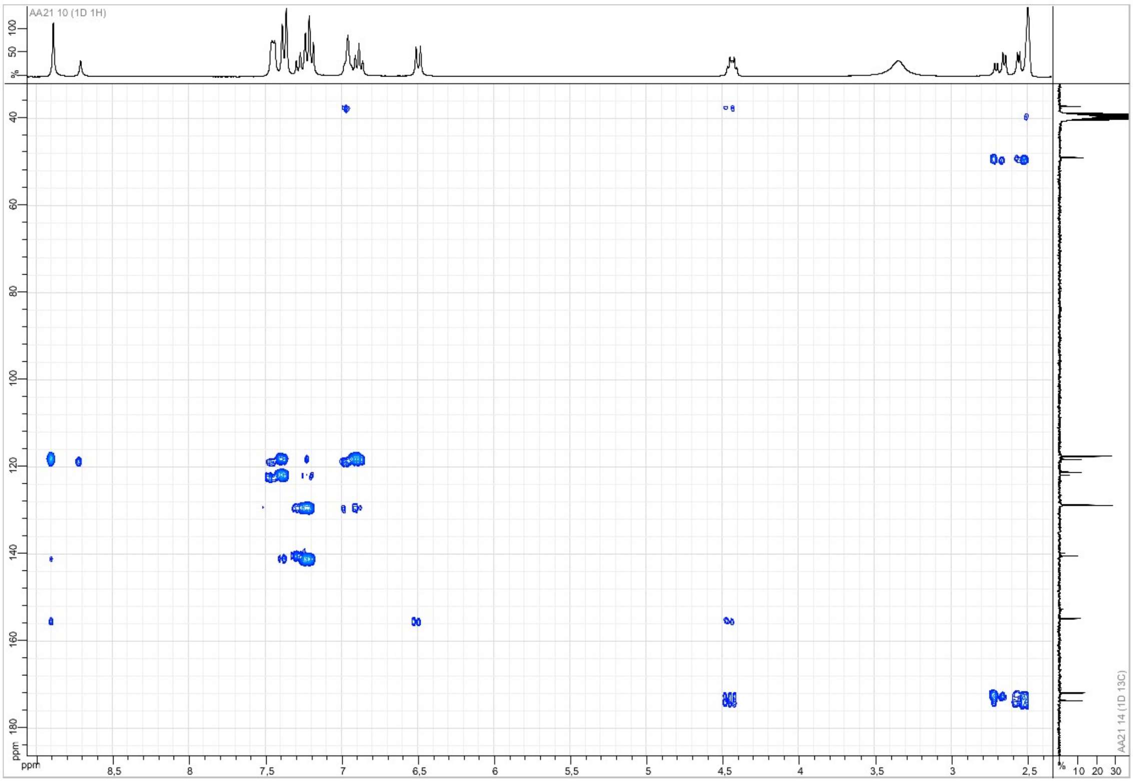

4. NMR spectra of 1c

^{1}H NMR (DMSO-d_6) of **1c** at 300 MHz

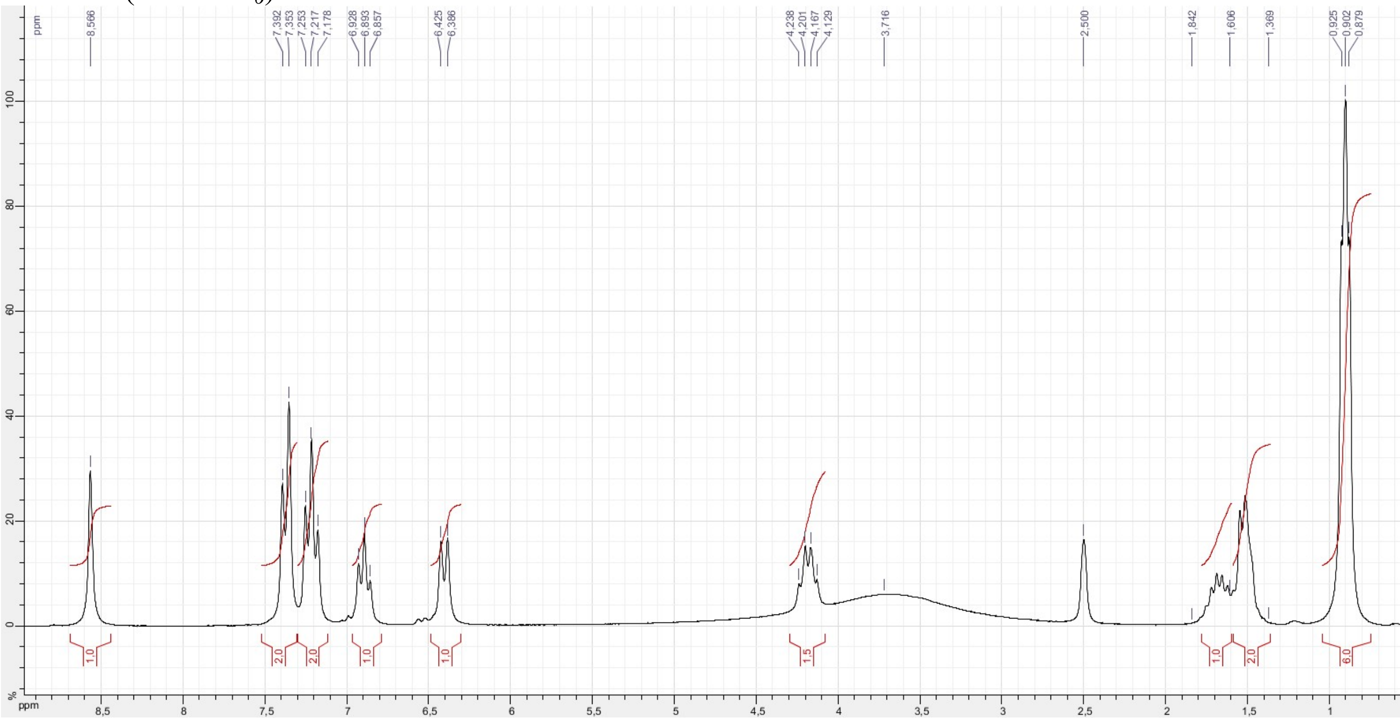

^{13}C NMR (DMSO-d_6) of **1c** at 75 MHz

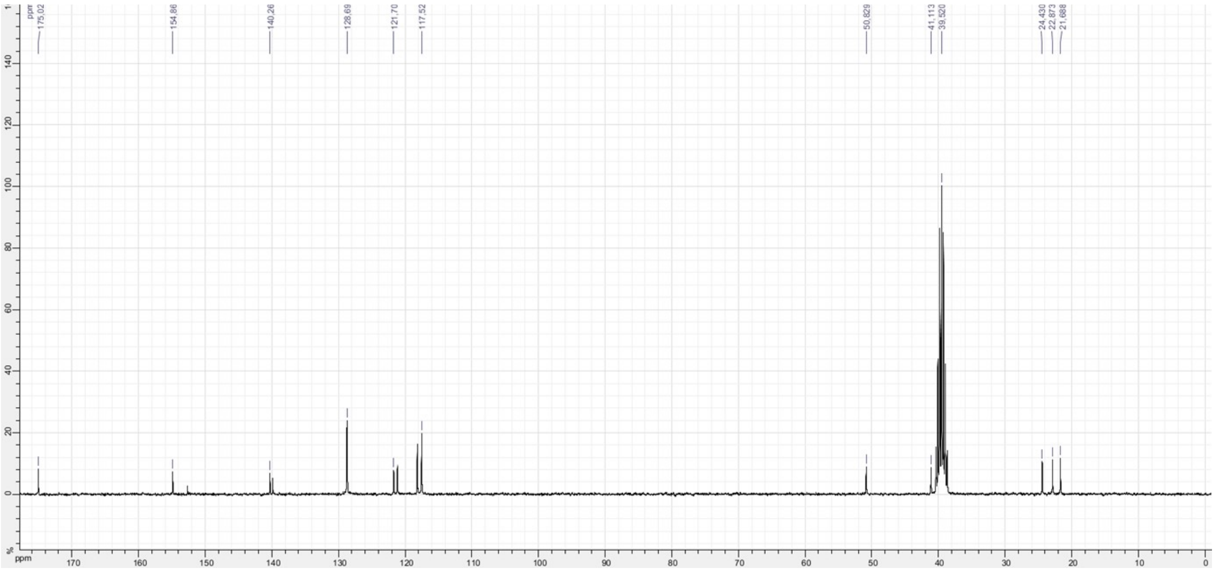

^{13}C (Dept135) NMR spectrum of **1c** in DMSO-d_6 at 75 MHz

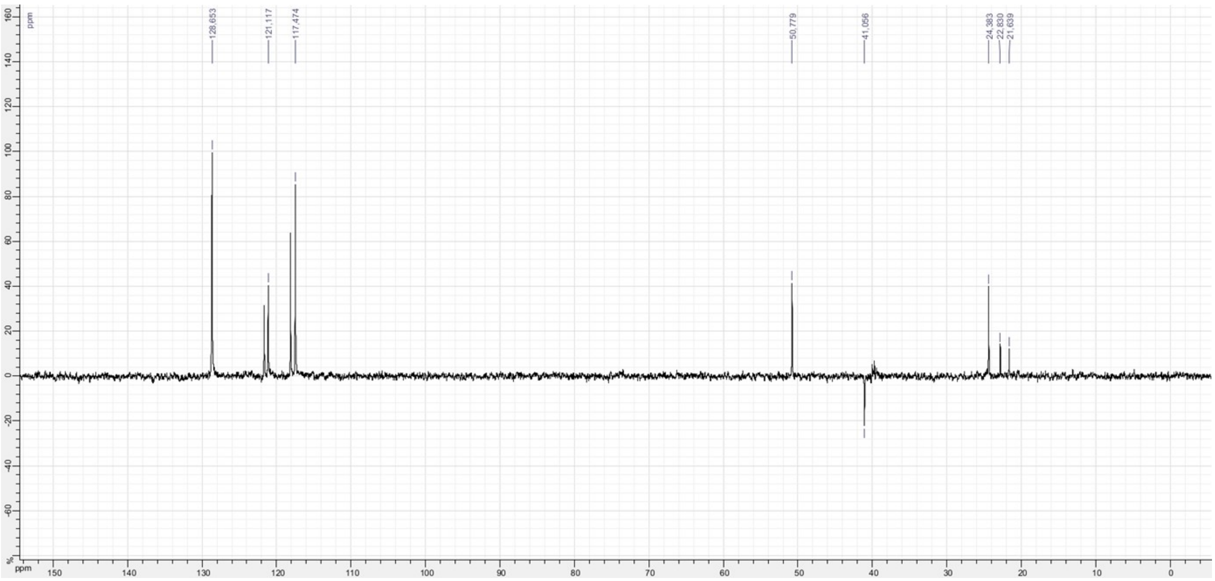

COSY NMR spectrum of **1c** in DMSO-d_6

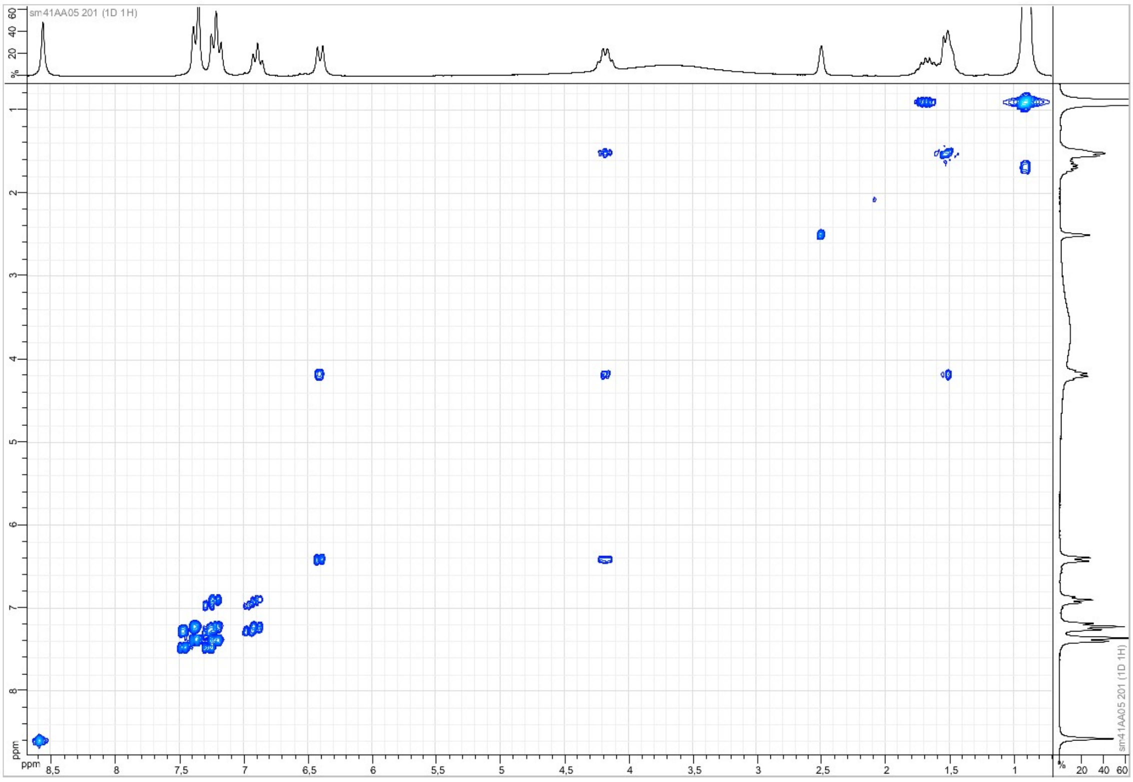

HSQC NMR spectrum of **1c** in DMSO-d_6

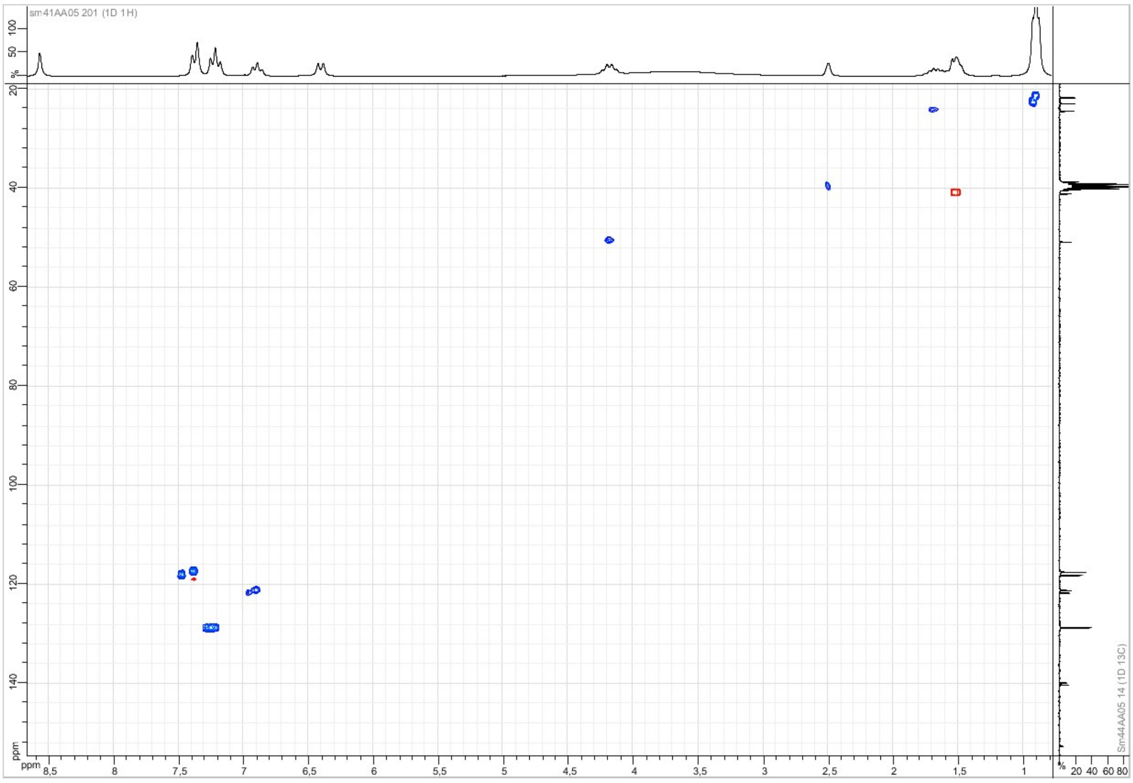

HMBC NMR spectrum of **1c** in DMSO-d_6

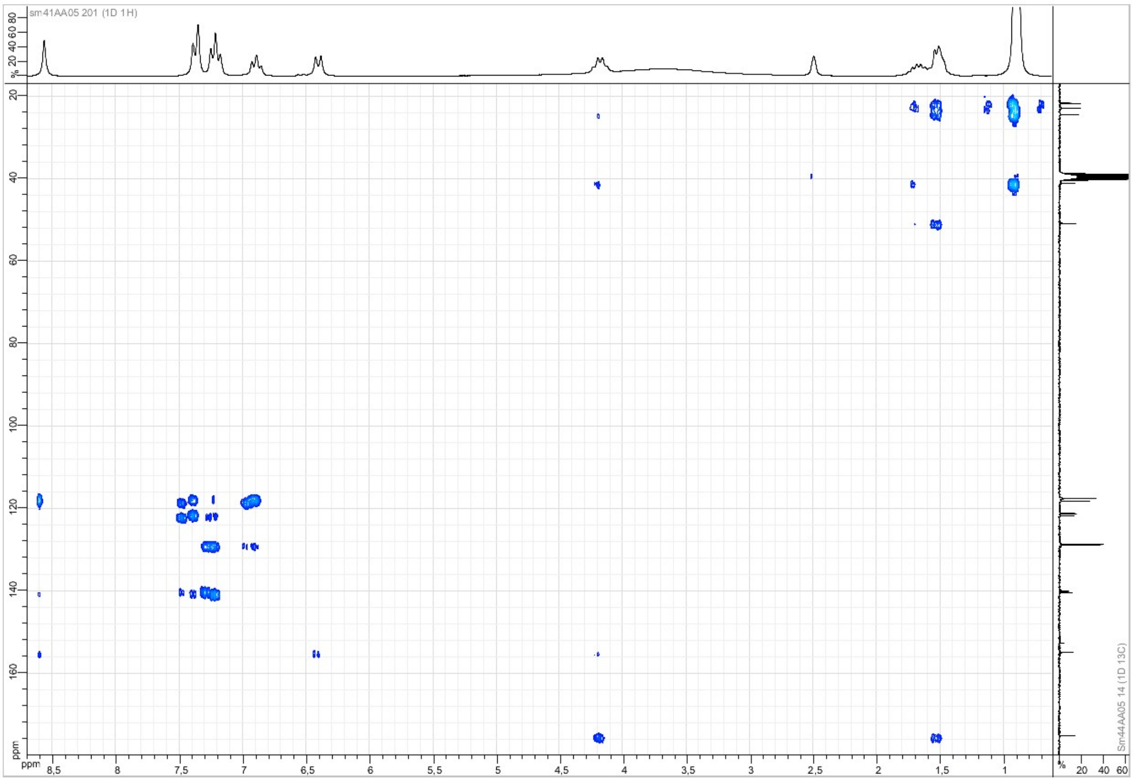

5. NMR spectra of 1d

^{1}H NMR (DMSO-d_6) of **1d** at 300 MHz

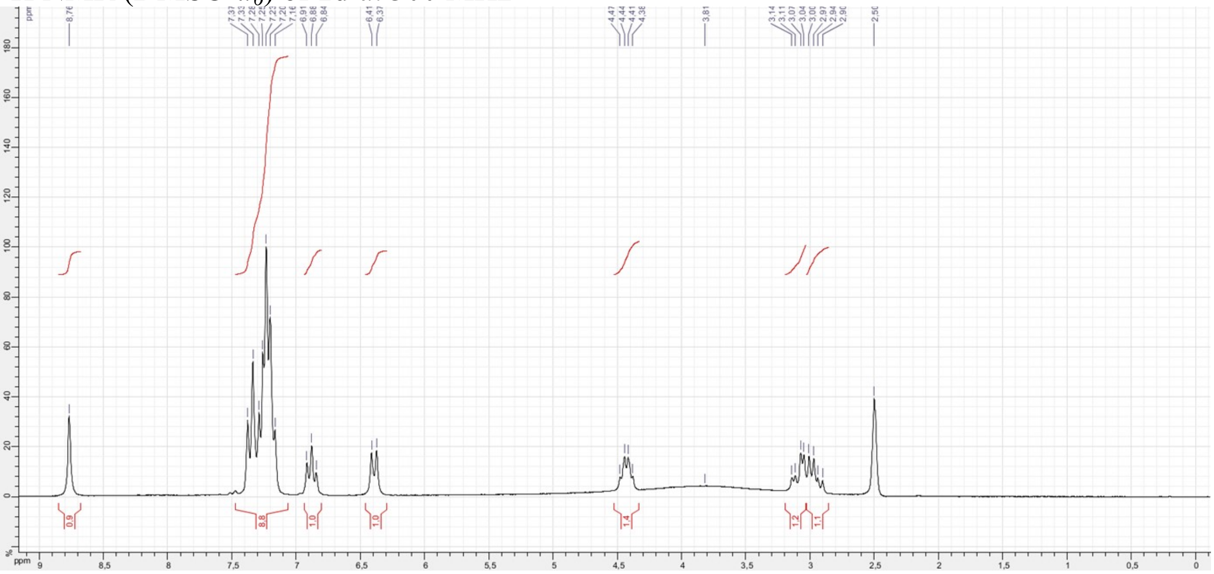

^{13}C NMR (DMSO-d_6) of **1d** at 75 MHz

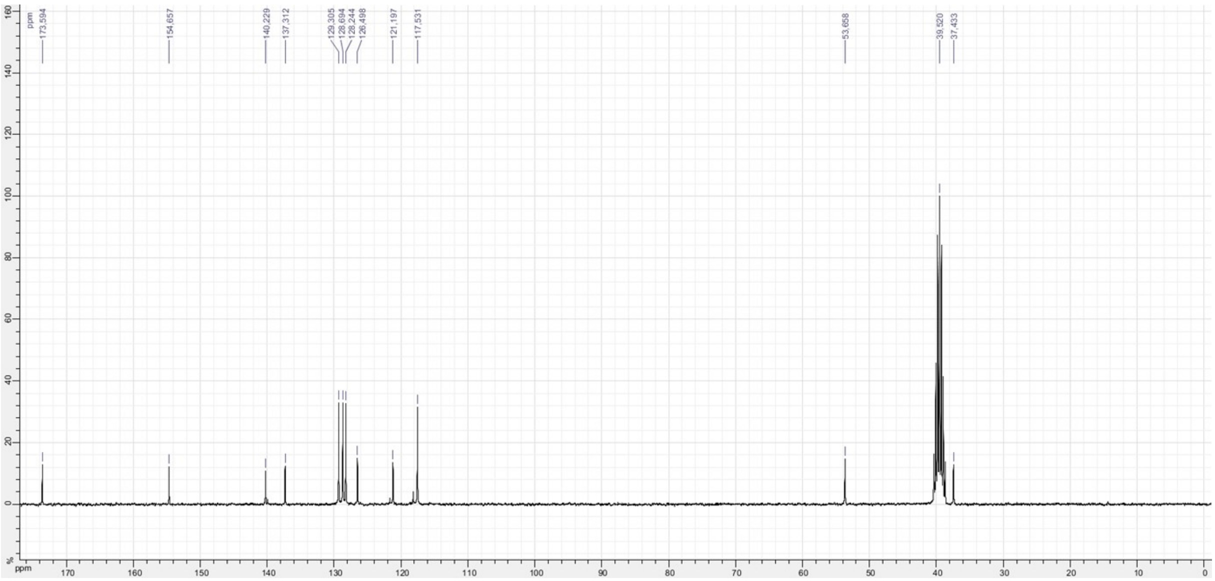

^{13}C (Dept135) NMR spectrum of **1d** in DMSO-d_6 at 75 MHz

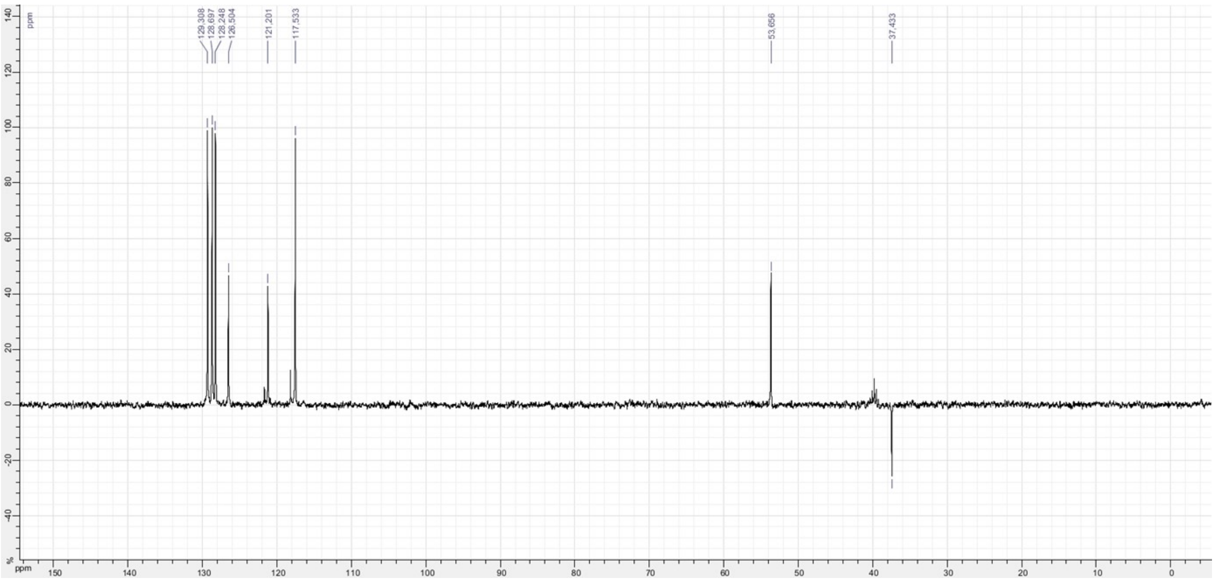

COSY NMR spectrum of **1d** in DMSO-d_6

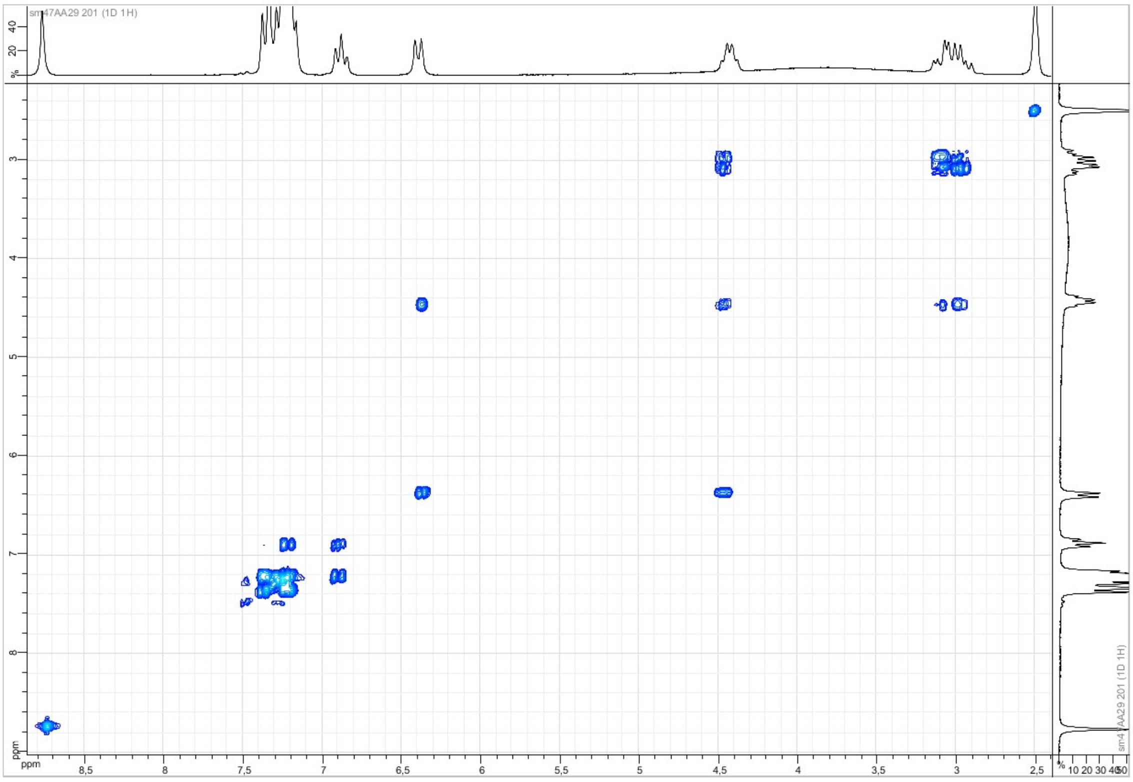

HSQC NMR spectrum of **1d** in DMSO-d_6

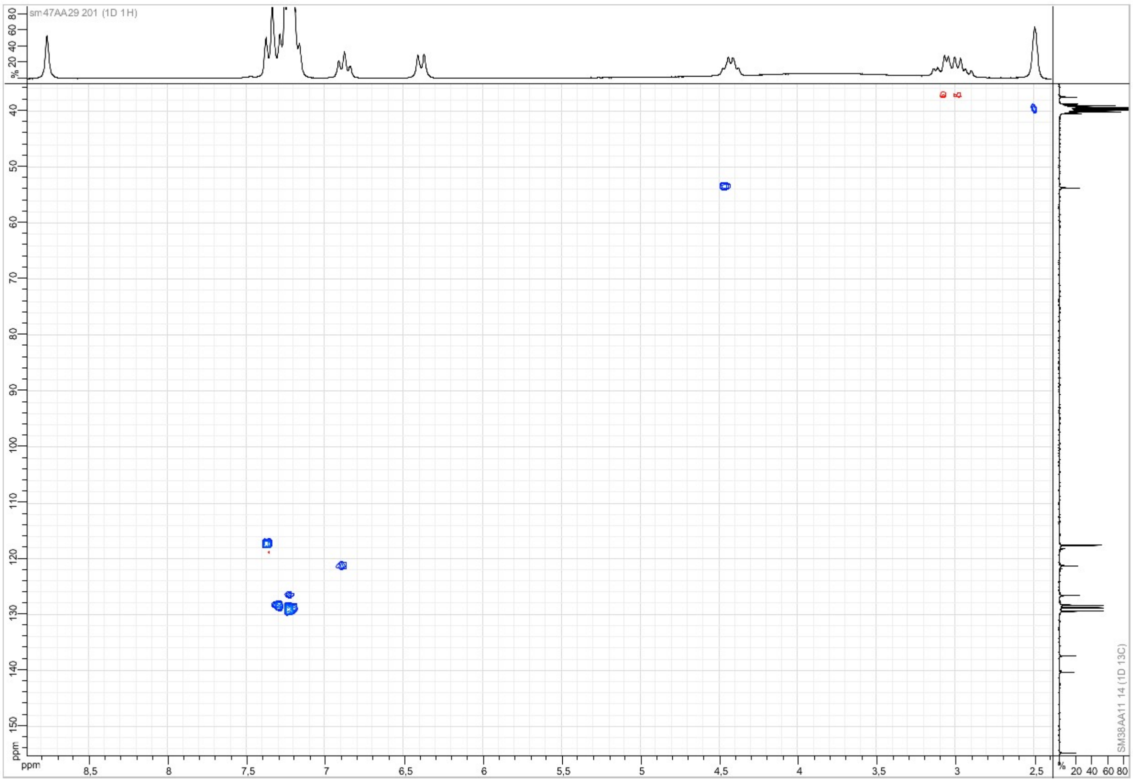

HMBC NMR spectrum of **1d** in DMSO-d_6

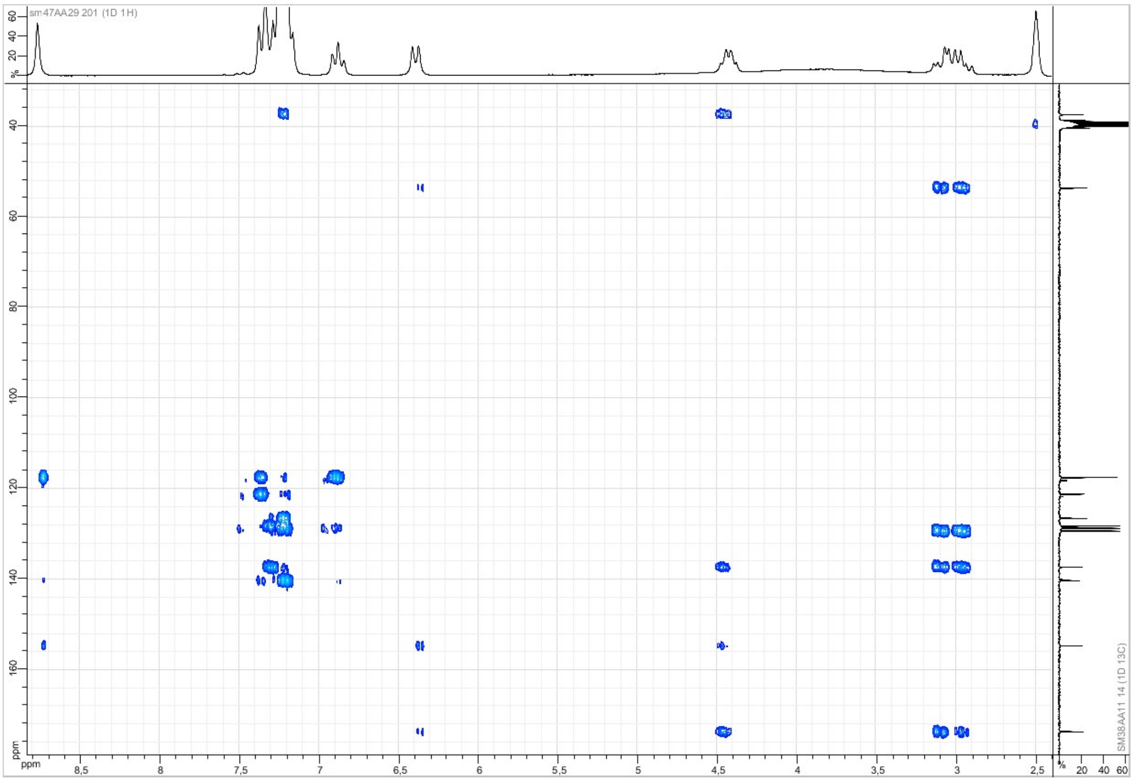

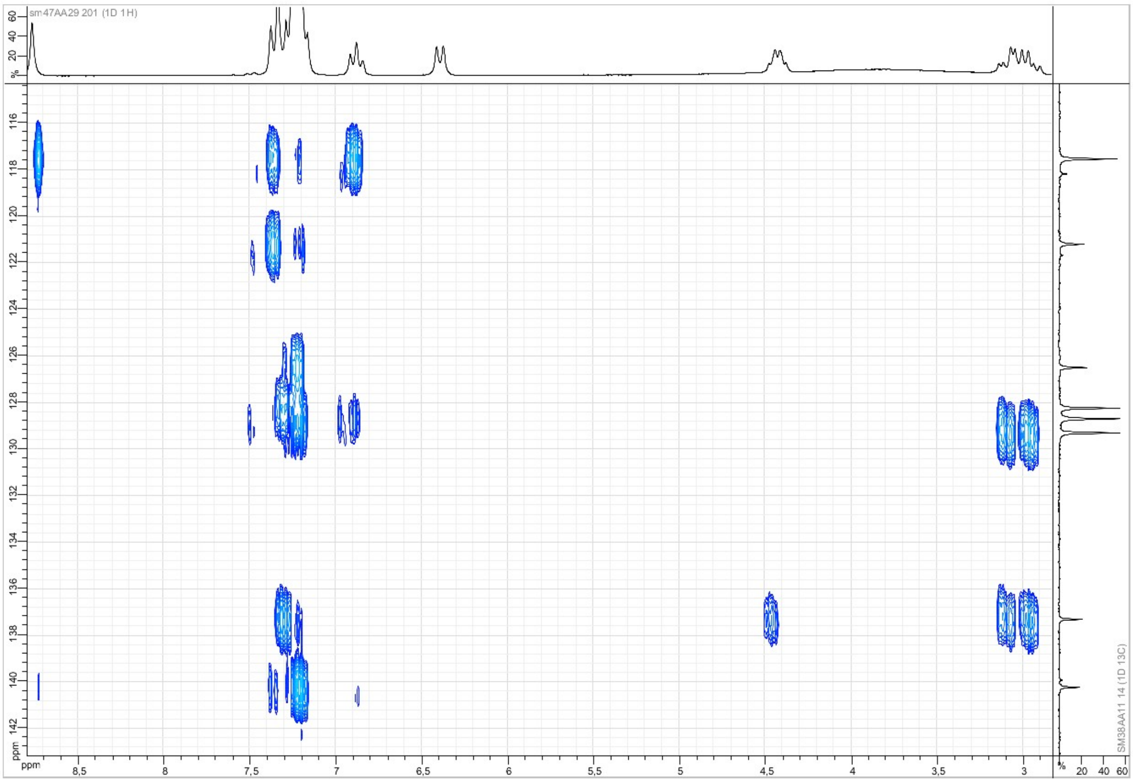

6. NMR spectra of 1e

^{1}H NMR (DMSO-d_6) of **1e** at 300 MHz

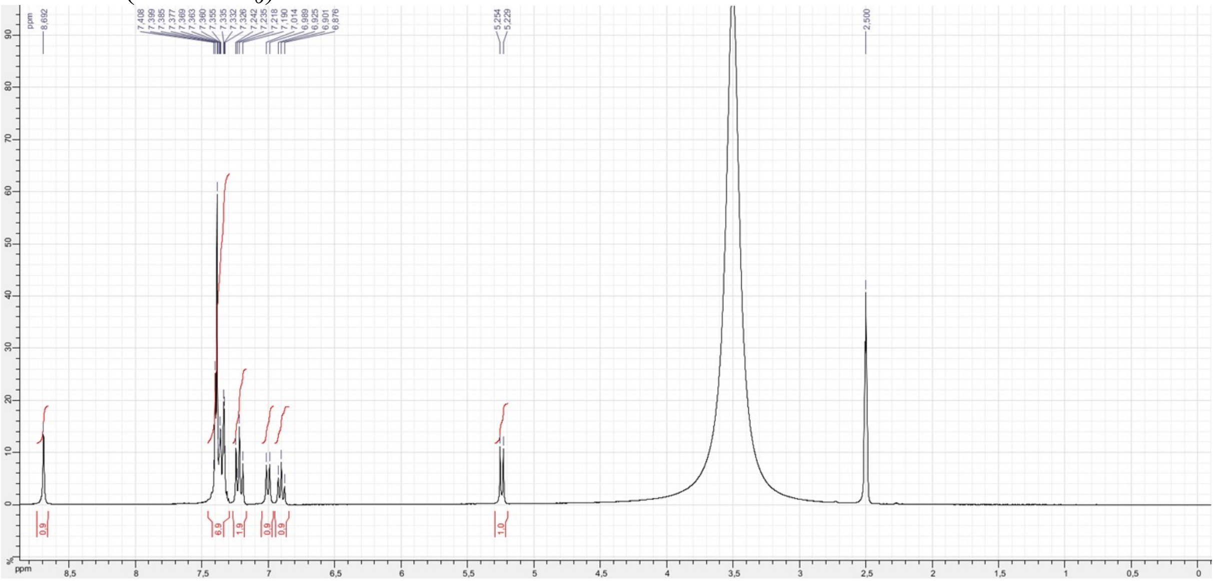

^{13}C NMR (DMSO-d_6) of **1e** at 75 MHz

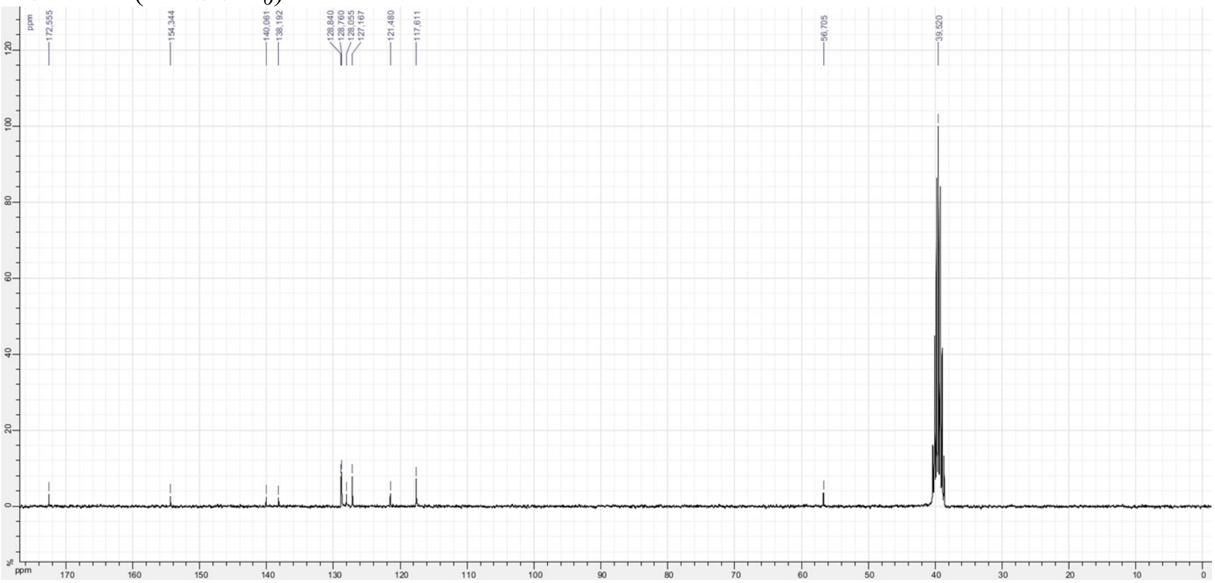

^{13}C (Dept135) NMR spectrum of **1e** in DMSO-d_6 at 75 MHz

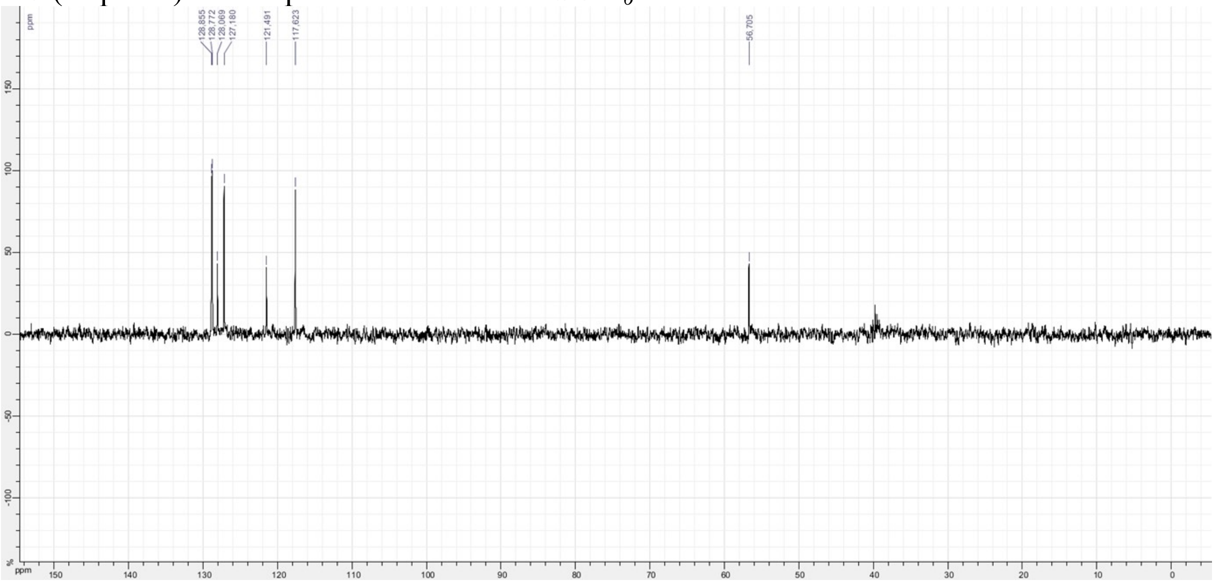

COSY NMR spectrum of **1e** in DMSO-d_6

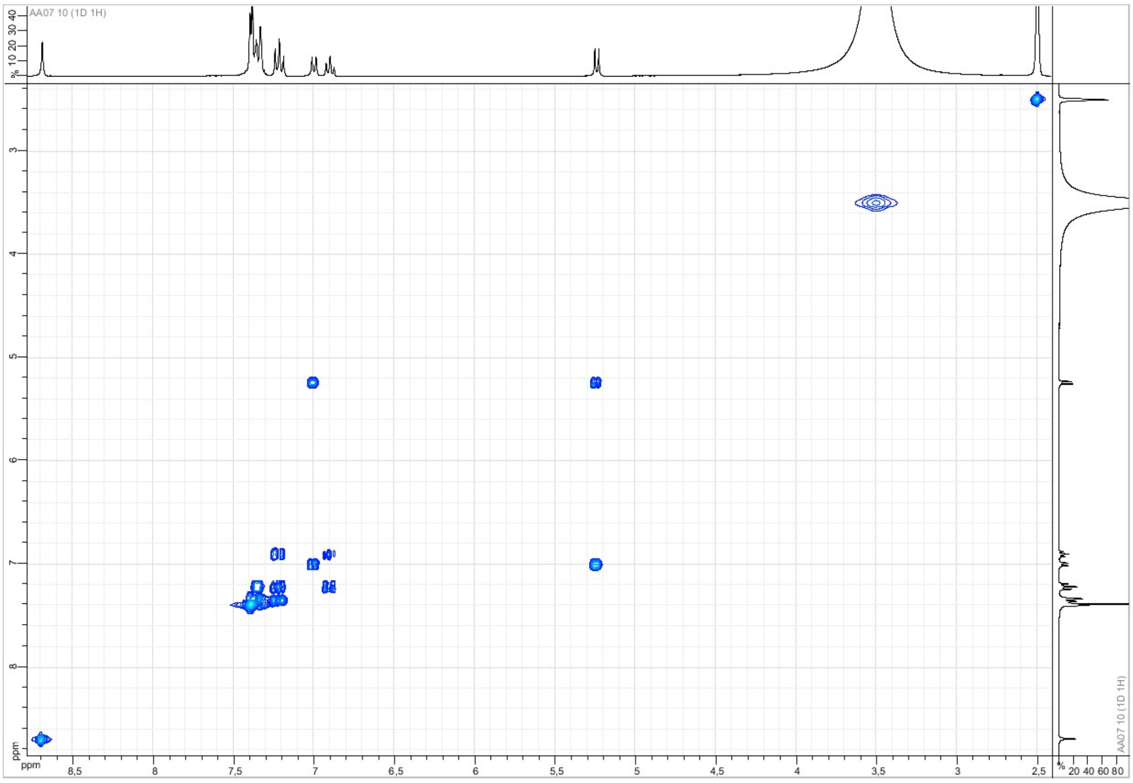

HSQC NMR spectrum of **1e** in DMSO-d_6

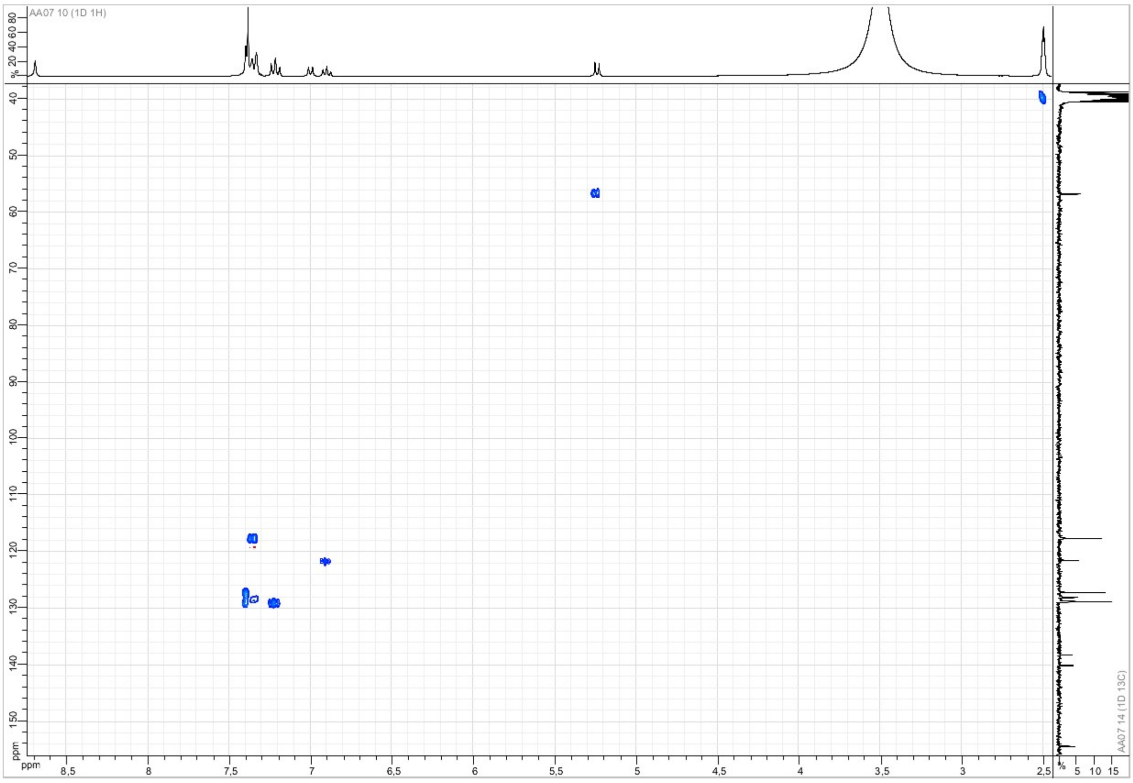

HMBC NMR spectrum of **1e** in DMSO-d_6

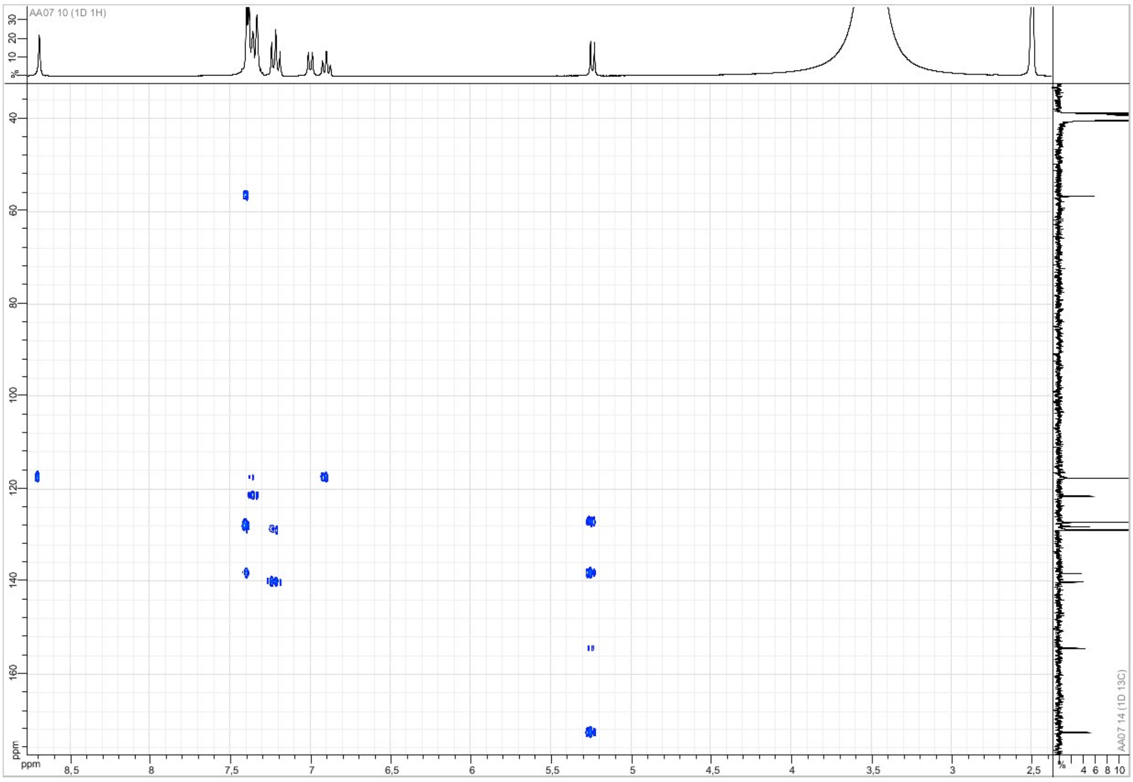

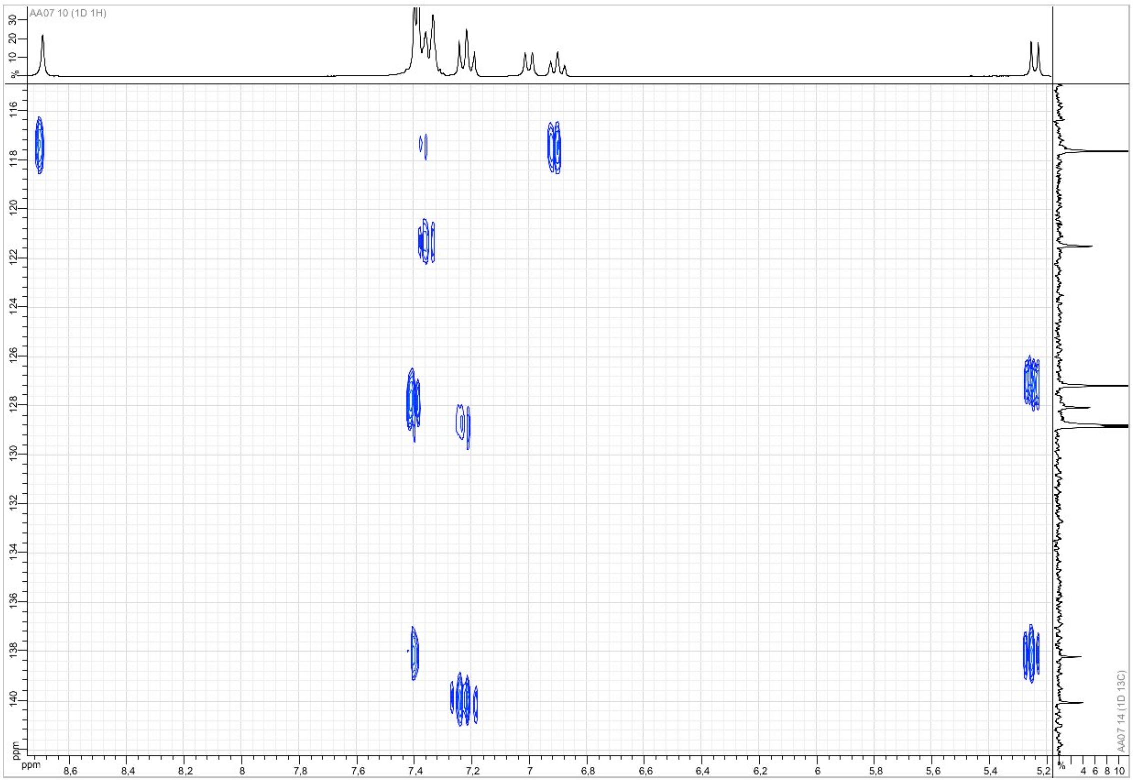

7. NMR spectra of 1f

^{1}H NMR (DMSO-d_6) of **1f** at 300 MHz

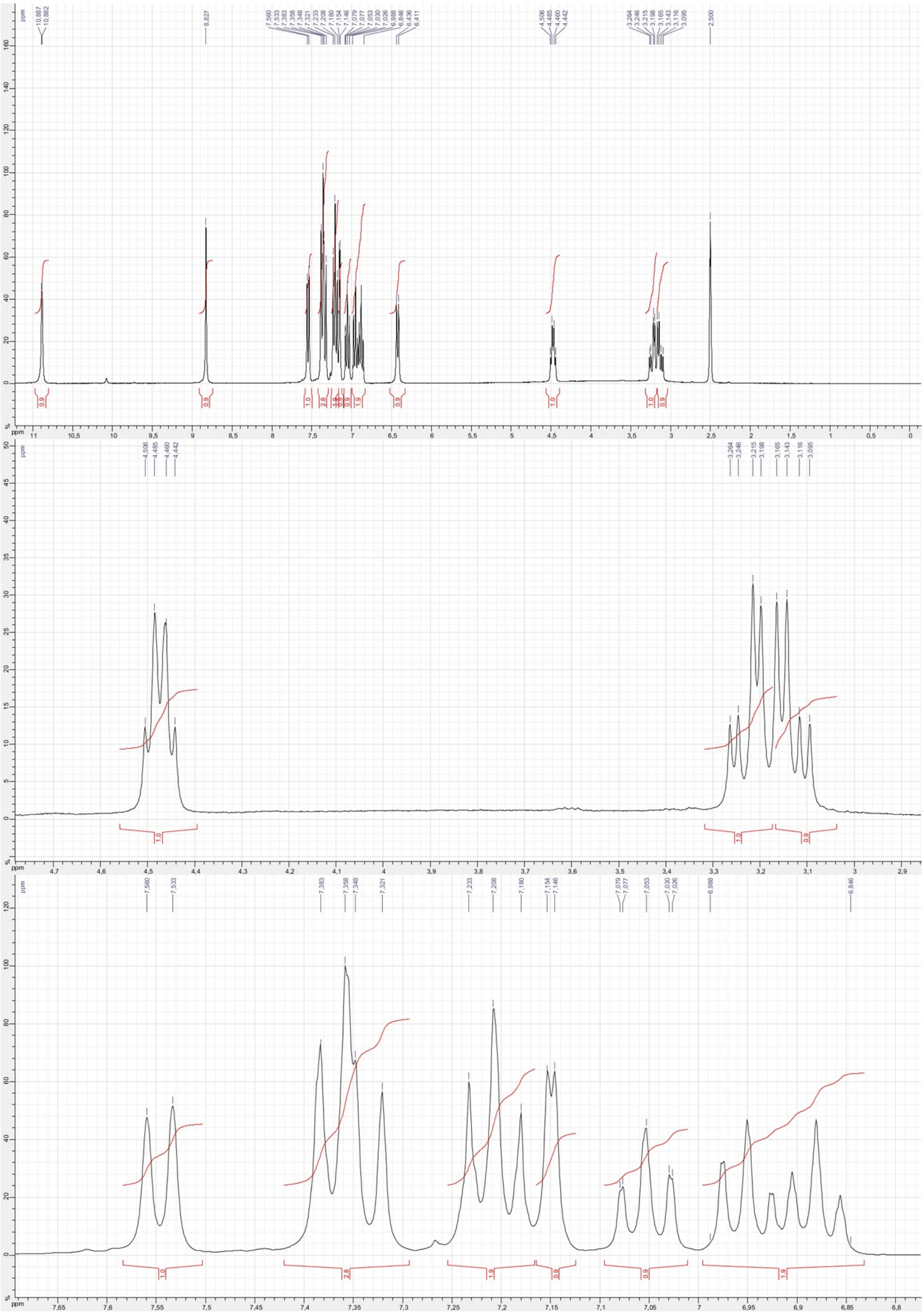

^{13}C NMR (DMSO-d_6) of **1f** at 75 MHz

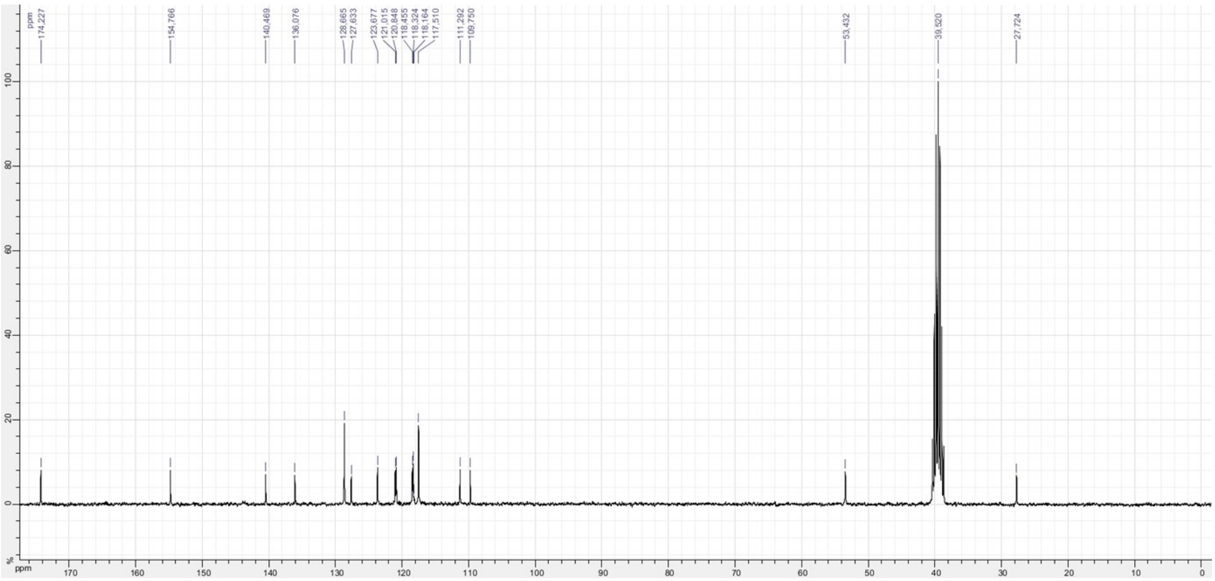

^{13}C (Dept135) NMR spectrum of **1f** in DMSO-d_6 at 75 MHz

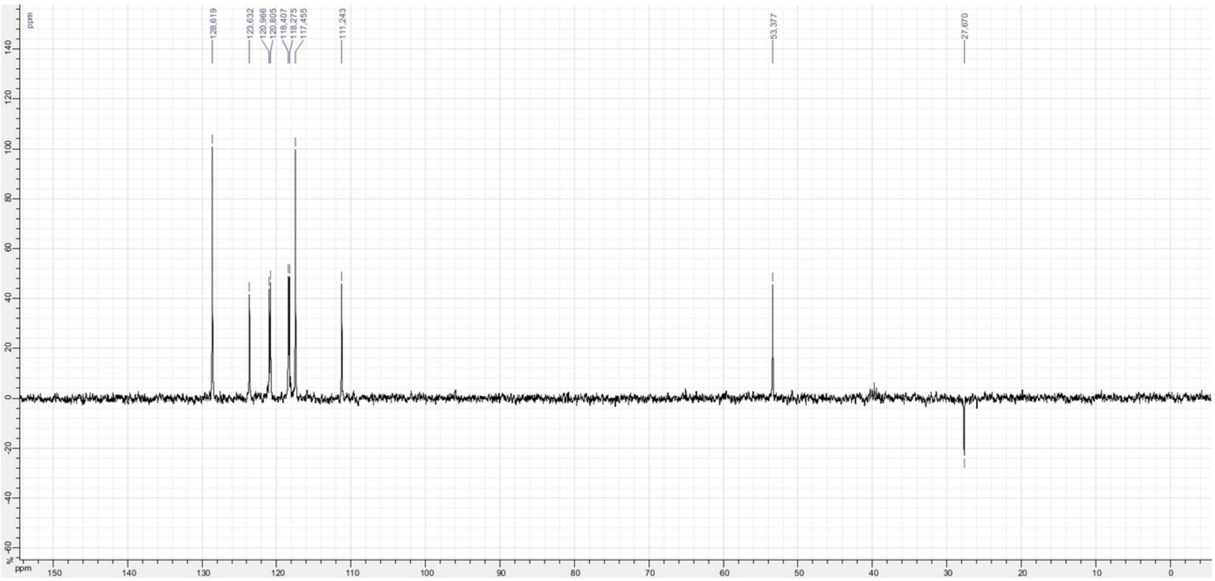

COSY NMR spectrum of **1f** in DMSO-d_6

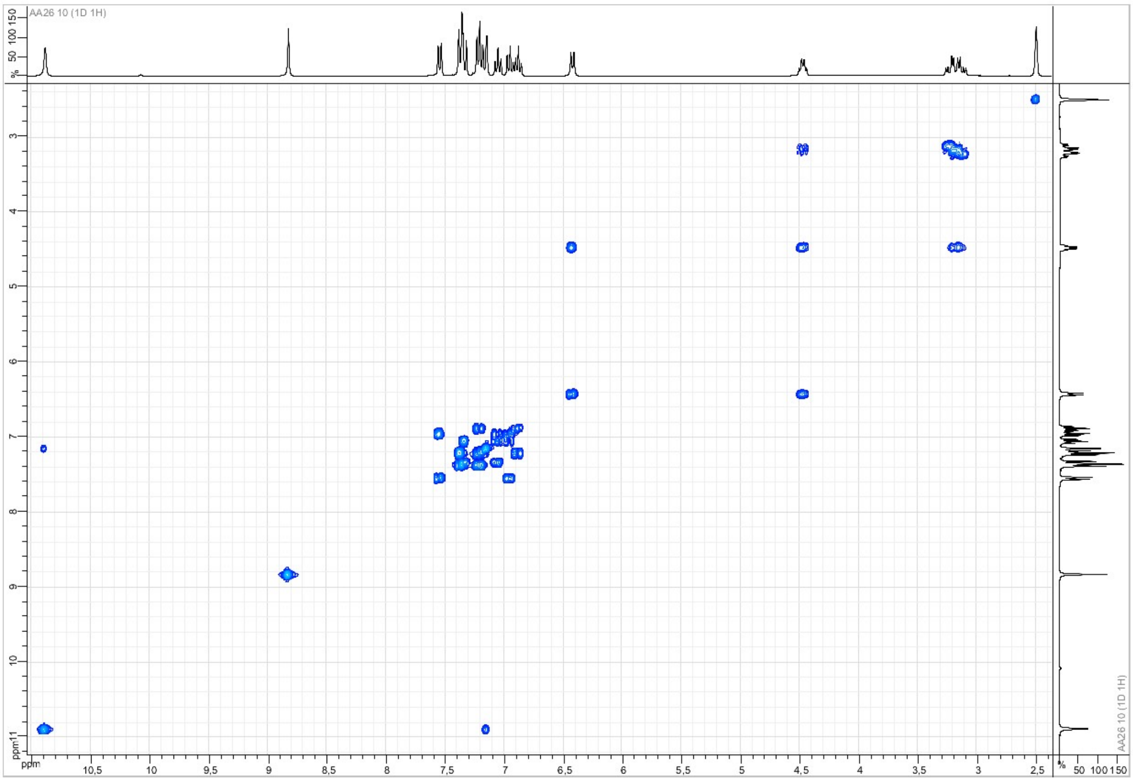

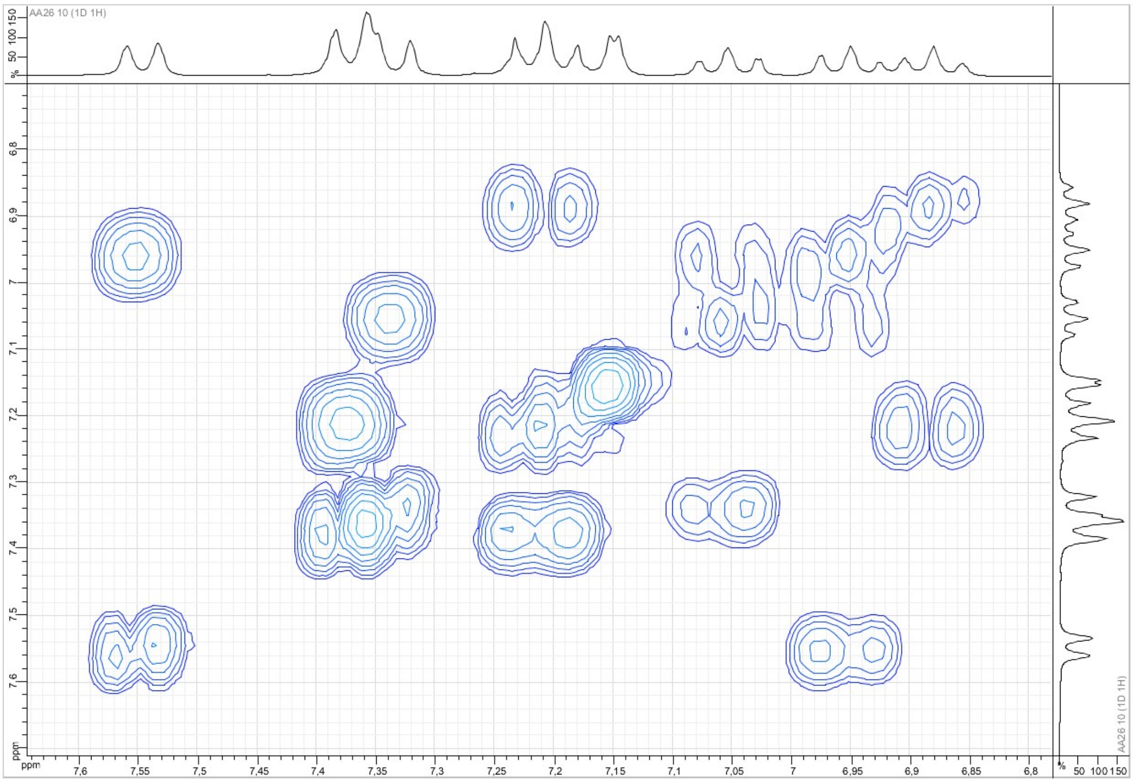

HSQC NMR spectrum of **1f** in DMSO-d_6

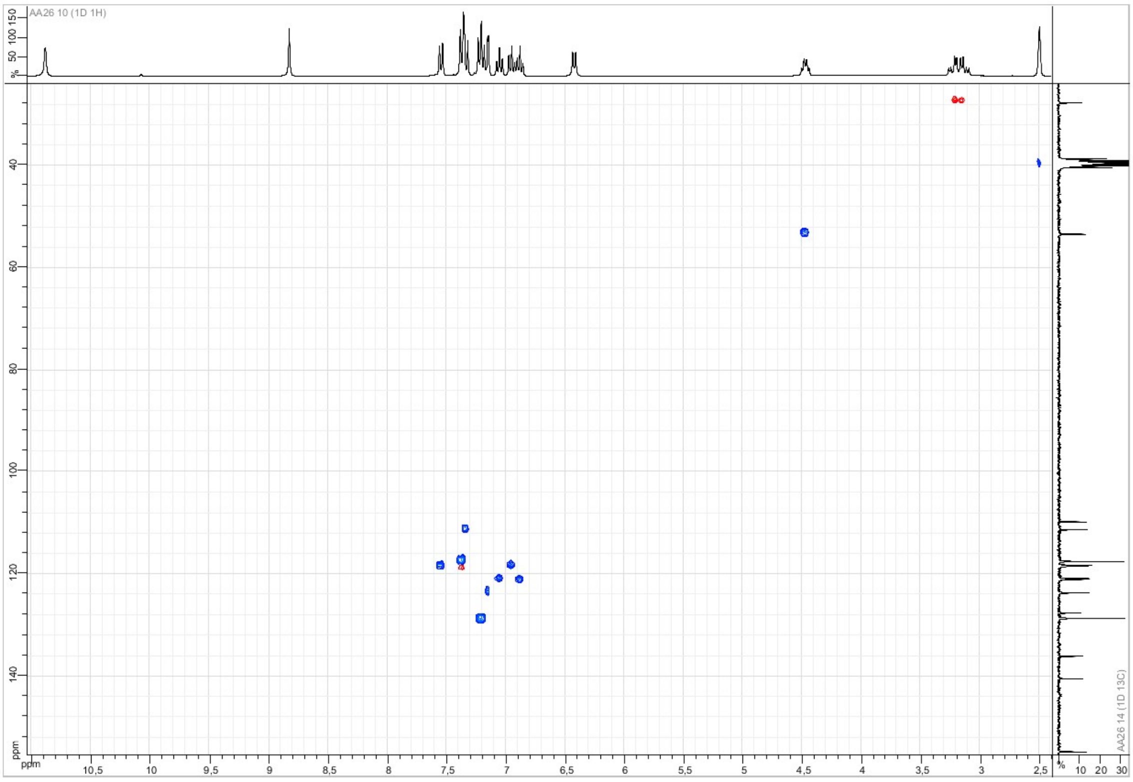

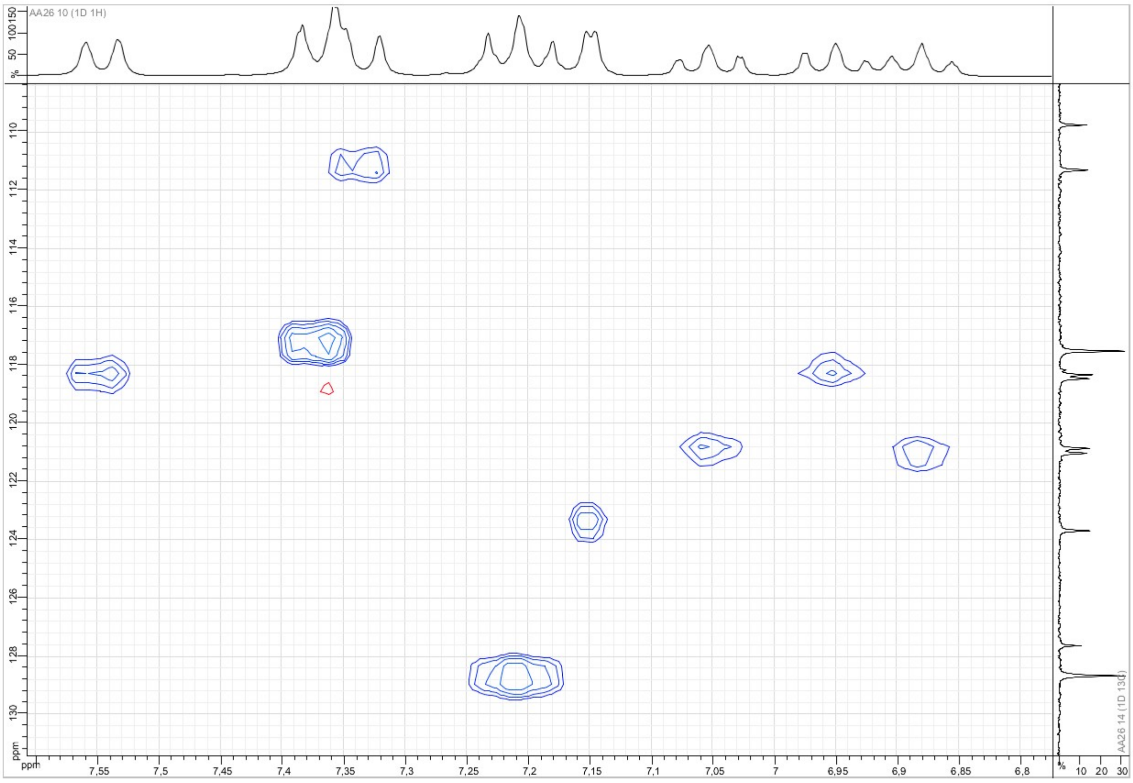

HMBC NMR spectrum of **1f** in DMSO-d_6

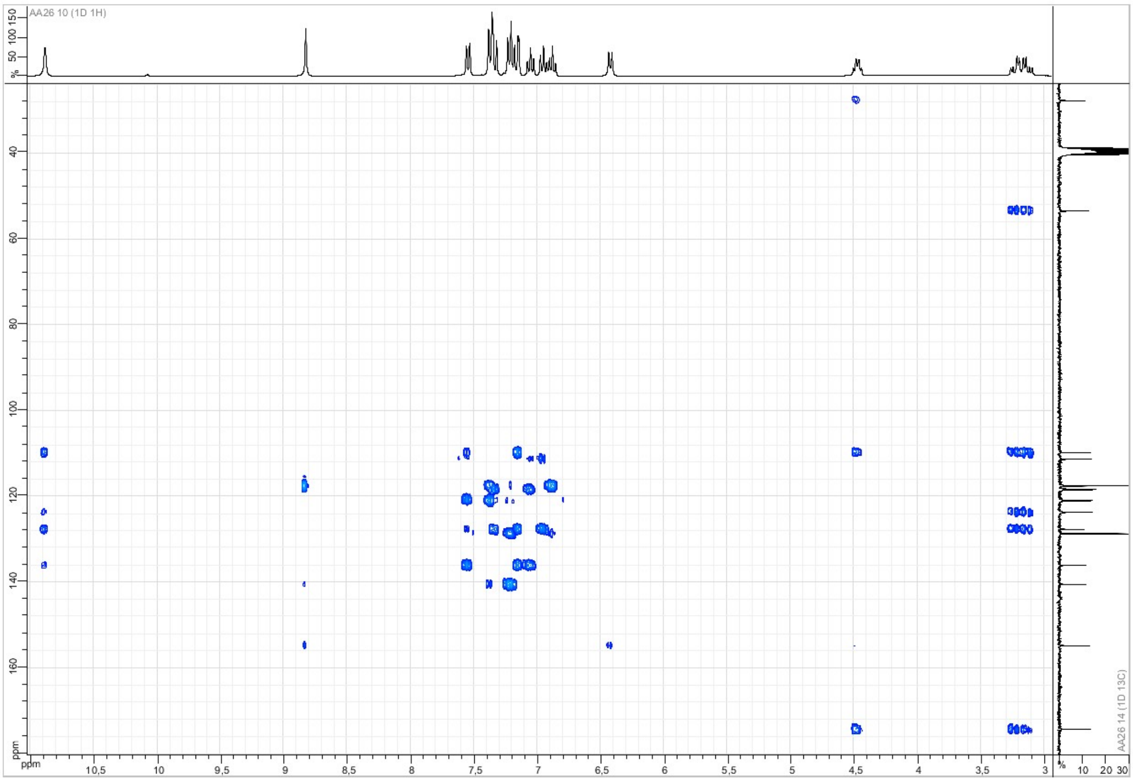

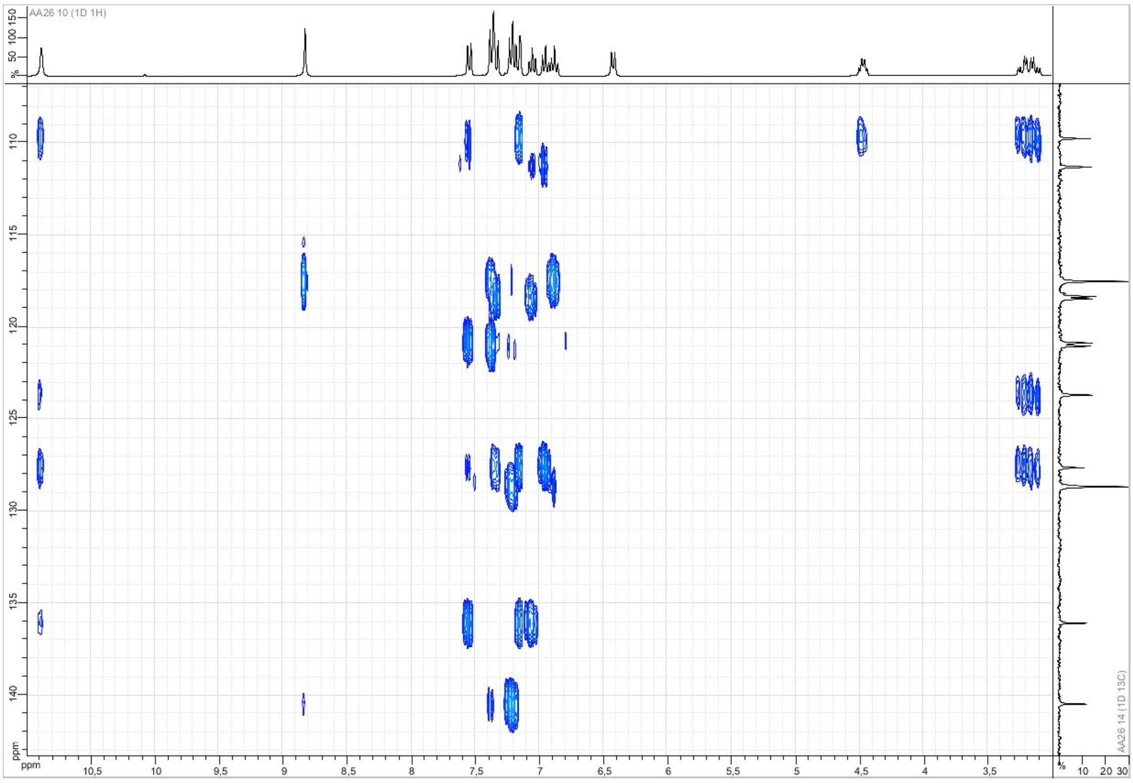

8. NMR spectra of 1g

^{1}H NMR (DMSO-d_6) of **1g** at 300 MHz

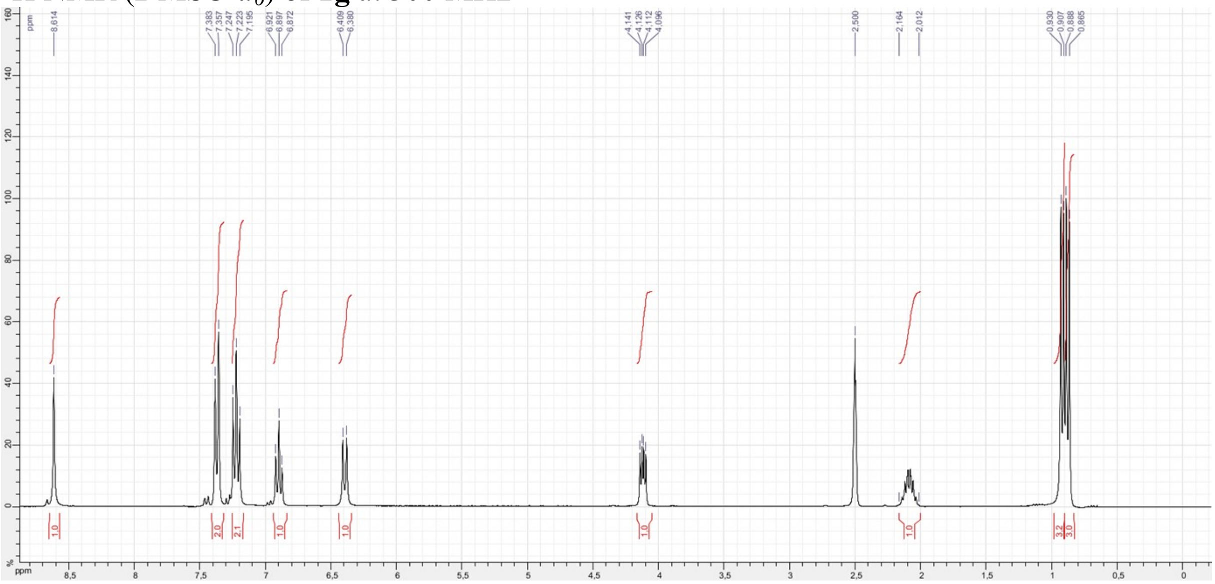

^{13}C NMR (DMSO-d_6) of **1g** at 75 MHz

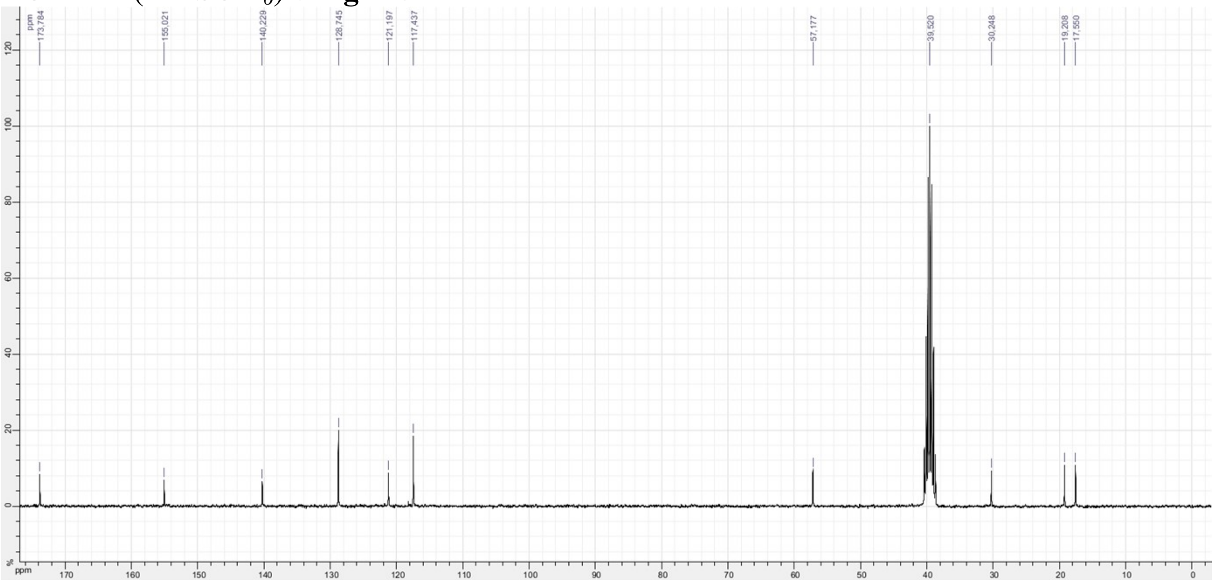

^{13}C (Dept135) NMR spectrum of **1g** in DMSO-d_6 at 75 MHz

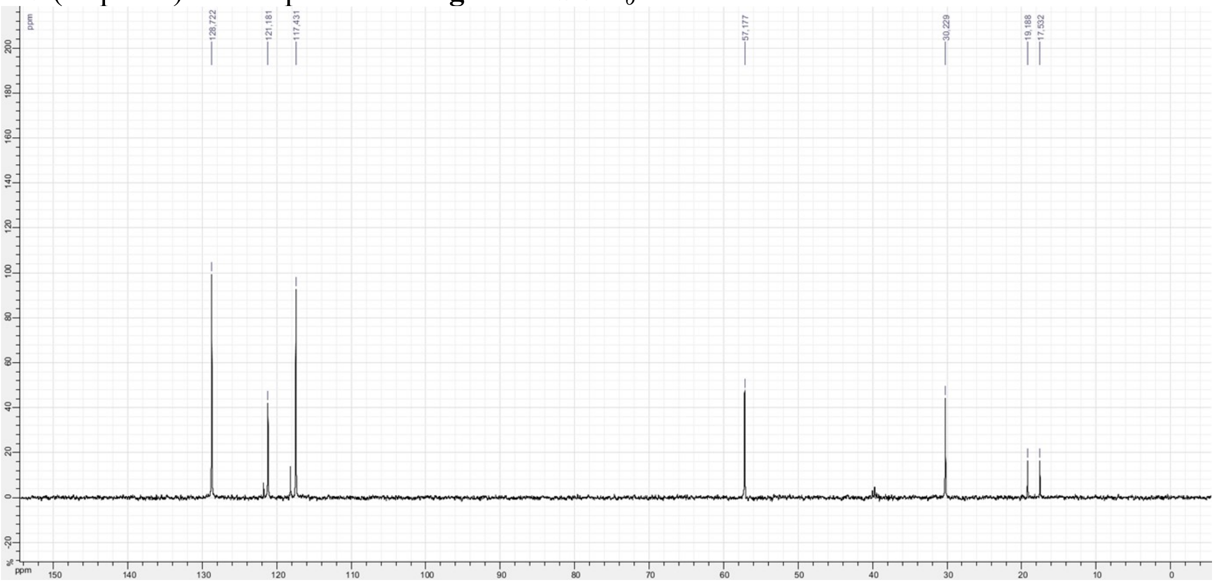

COSY NMR spectrum of **1g** in DMSO-d_6

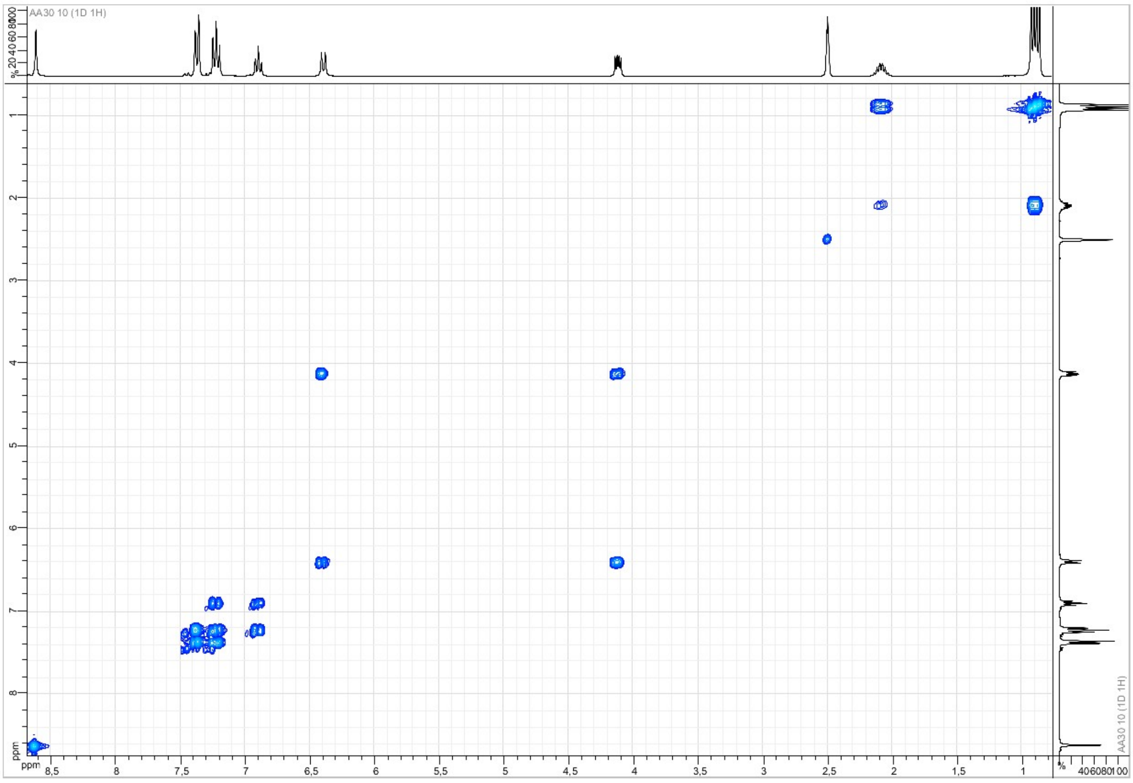

HSQC NMR spectrum of **1g** in DMSO-d_6

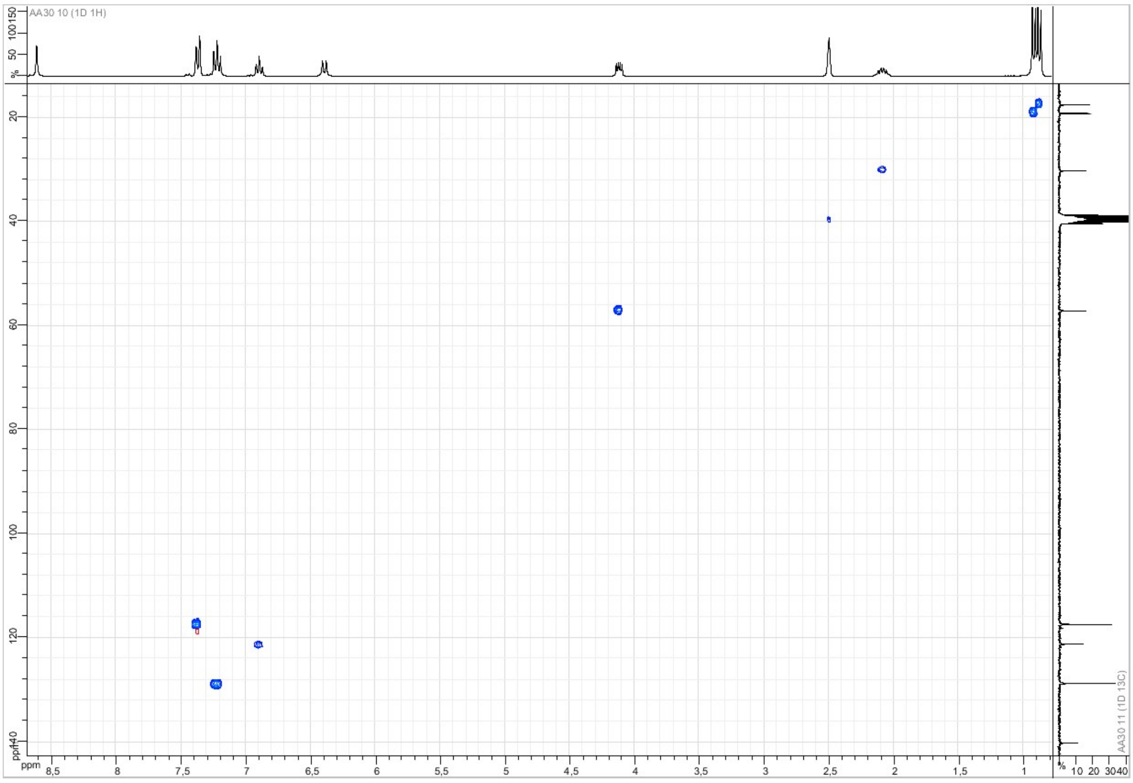

HMBC NMR spectrum of **1g** in DMSO-d_6

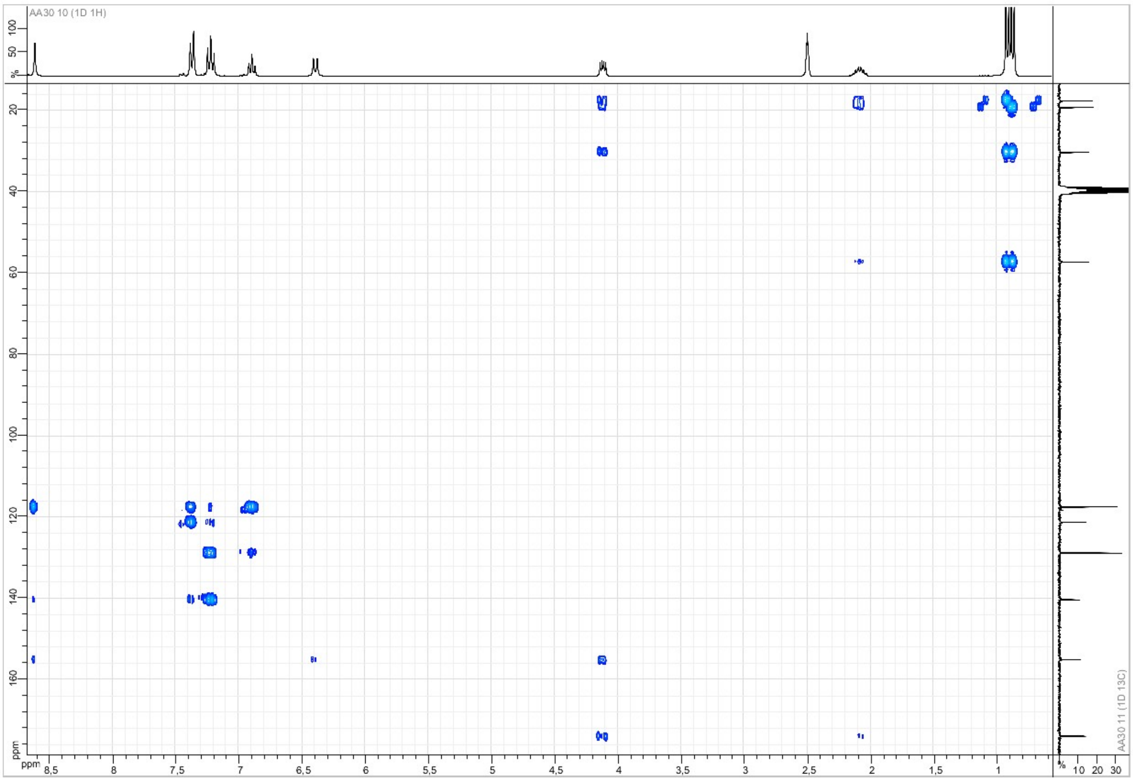

NMR spectra of 1h

^{1}H NMR (DMSO-d_6) of **1h** at 300 MHz

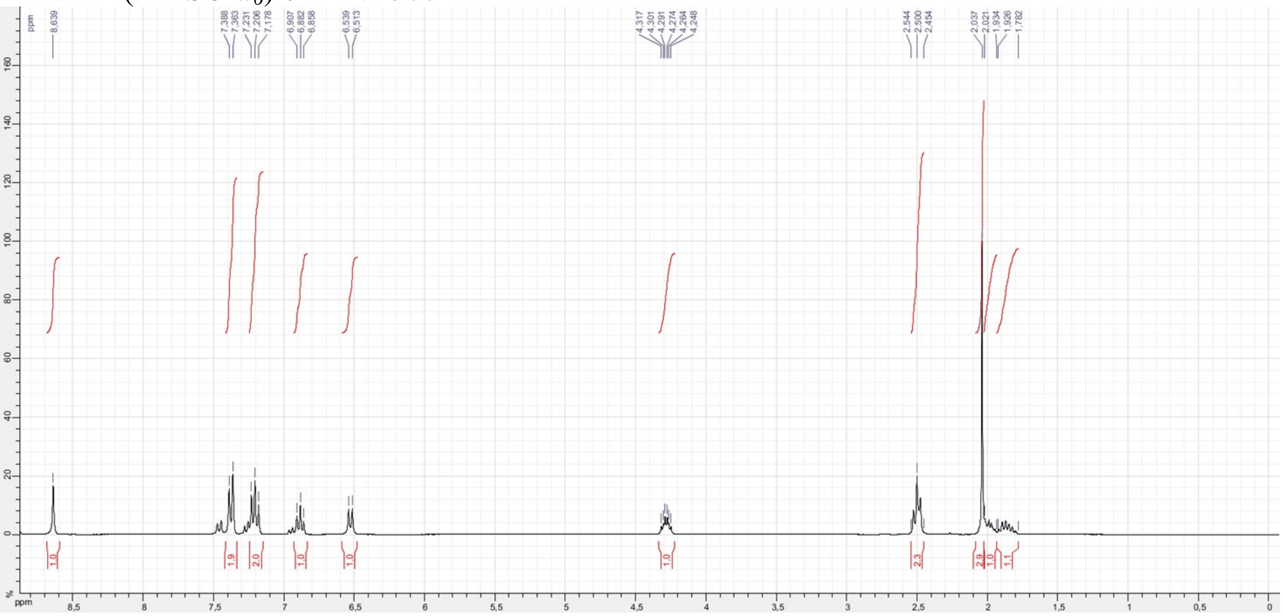

1

[3]C NMR (DMSO-d_6) of **1h** at 75 MHz

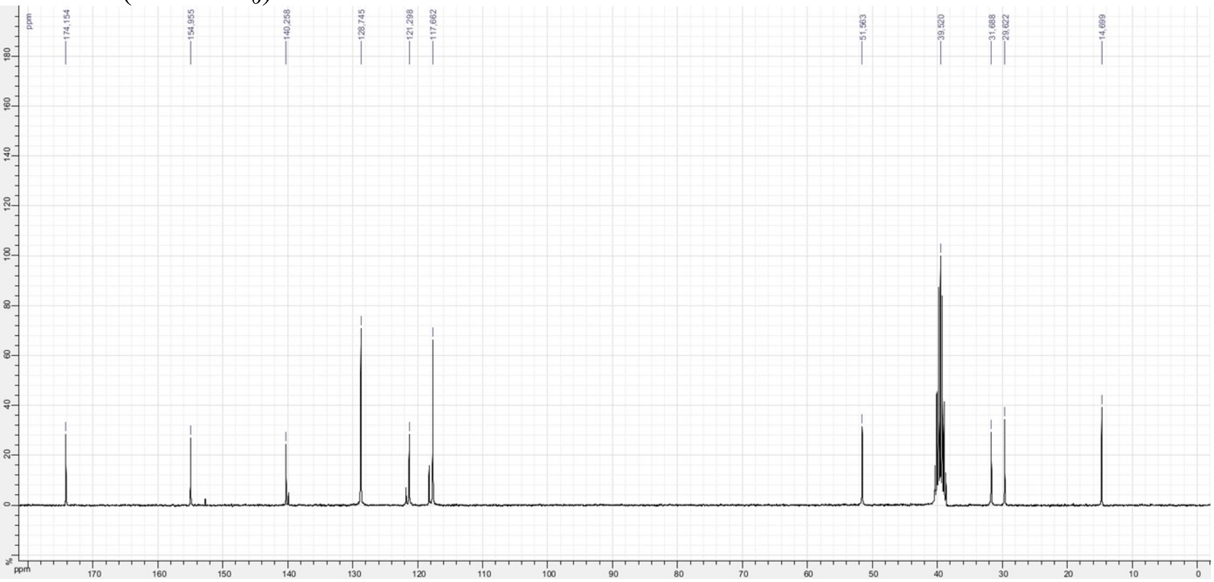

^{13}C (Dept135) NMR spectrum of **1h** in DMSO-d_6 at 75 MHz

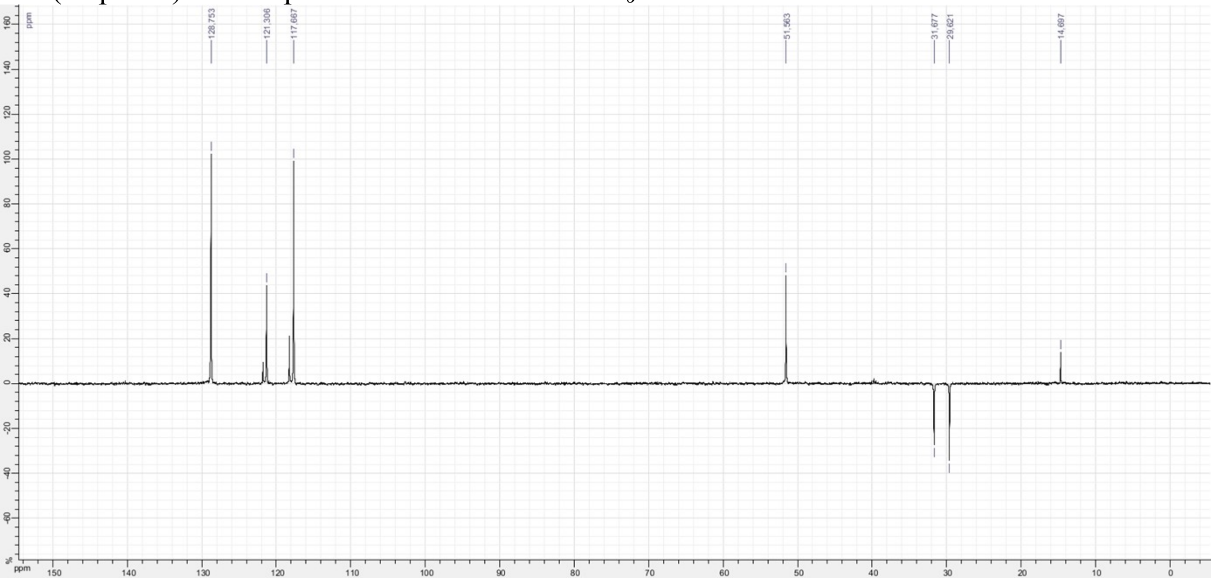

COSY NMR spectrum of **1h** in DMSO-d_6

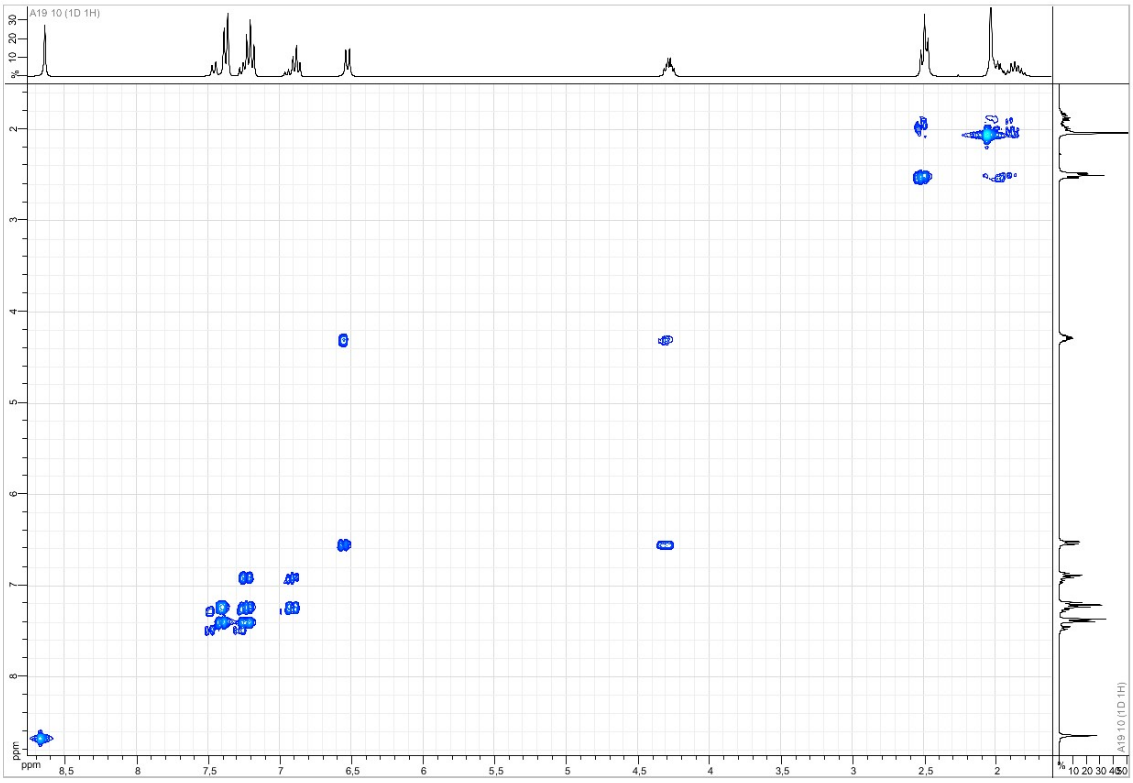

HSQC NMR spectrum of **1h** in DMSO-d_6

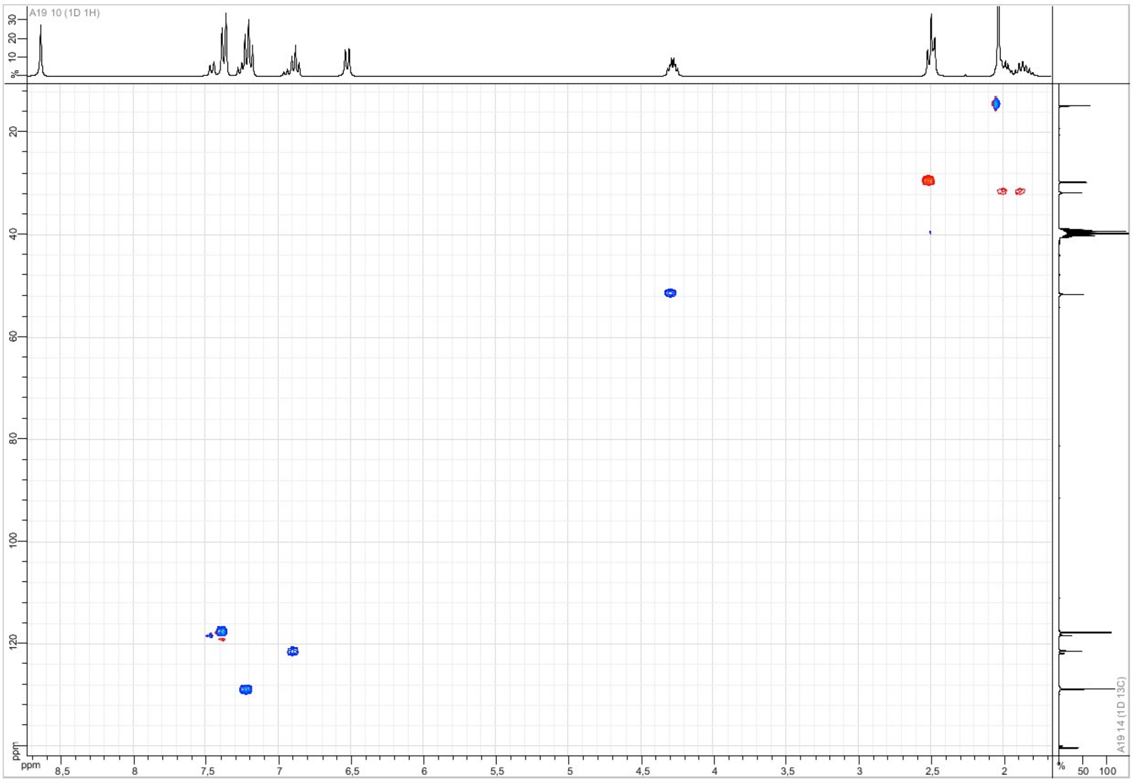

HMBC NMR spectrum of **1h** in DMSO-d_6

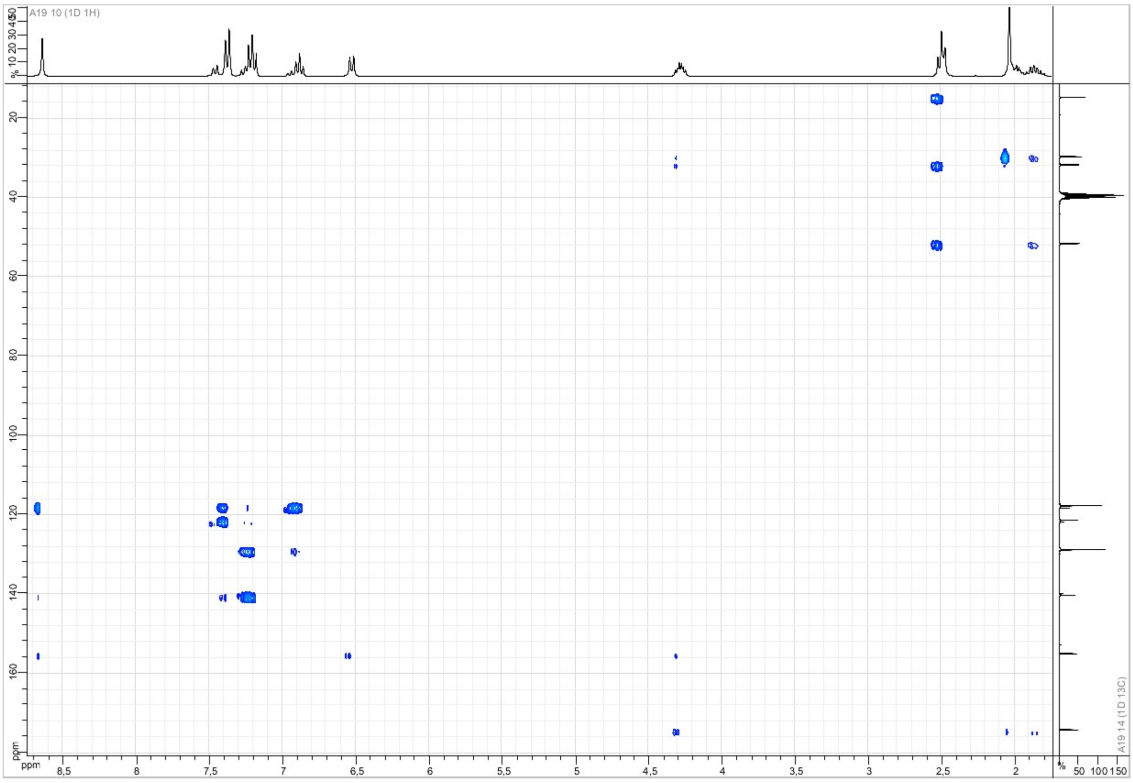

9. NMR spectra of 2a

^{1}H NMR (DMSO-d_6) of **2a** at 300 MHz

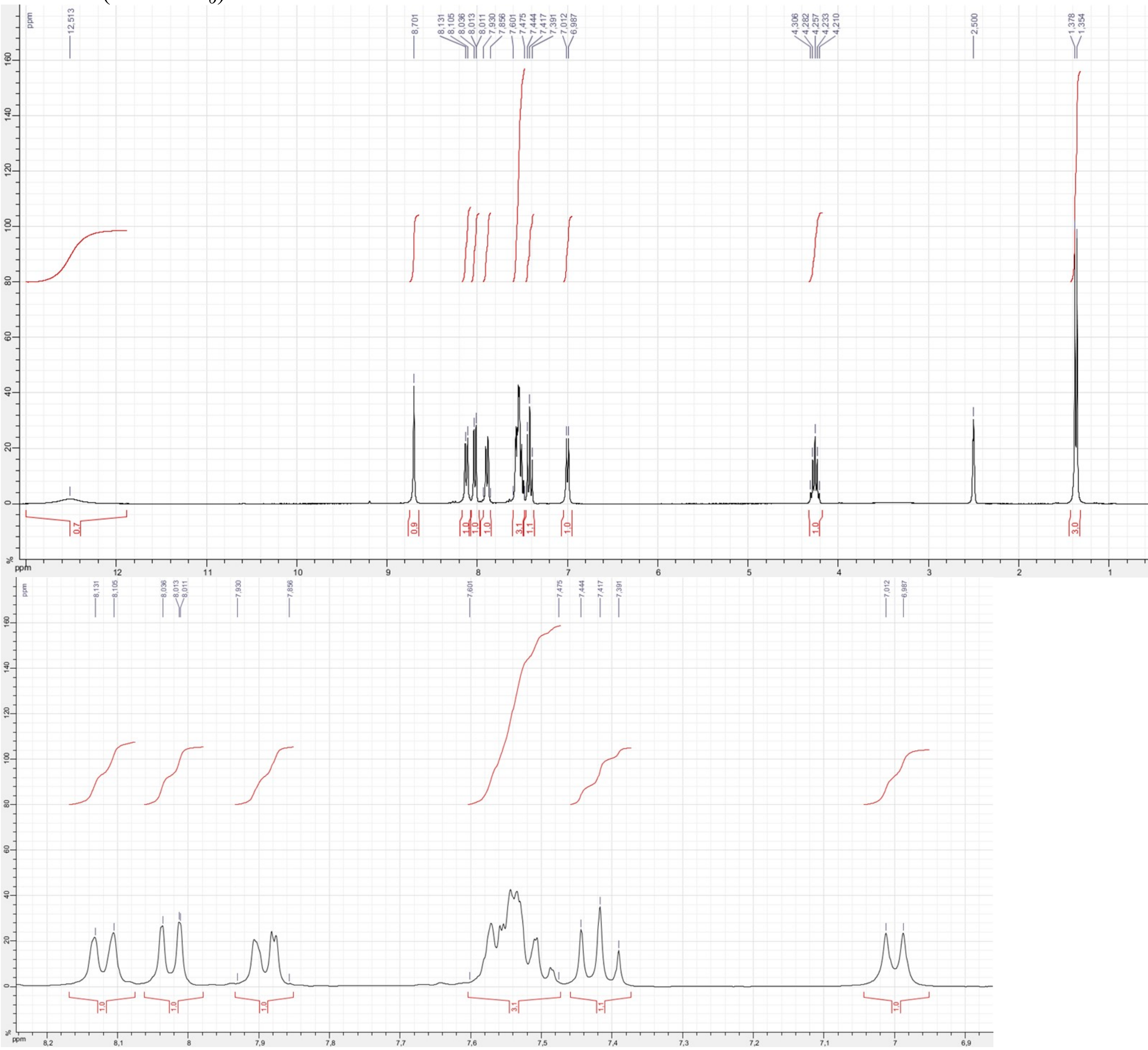

^{13}C NMR (DMSO-d_6) of **2a** at 75 MHz

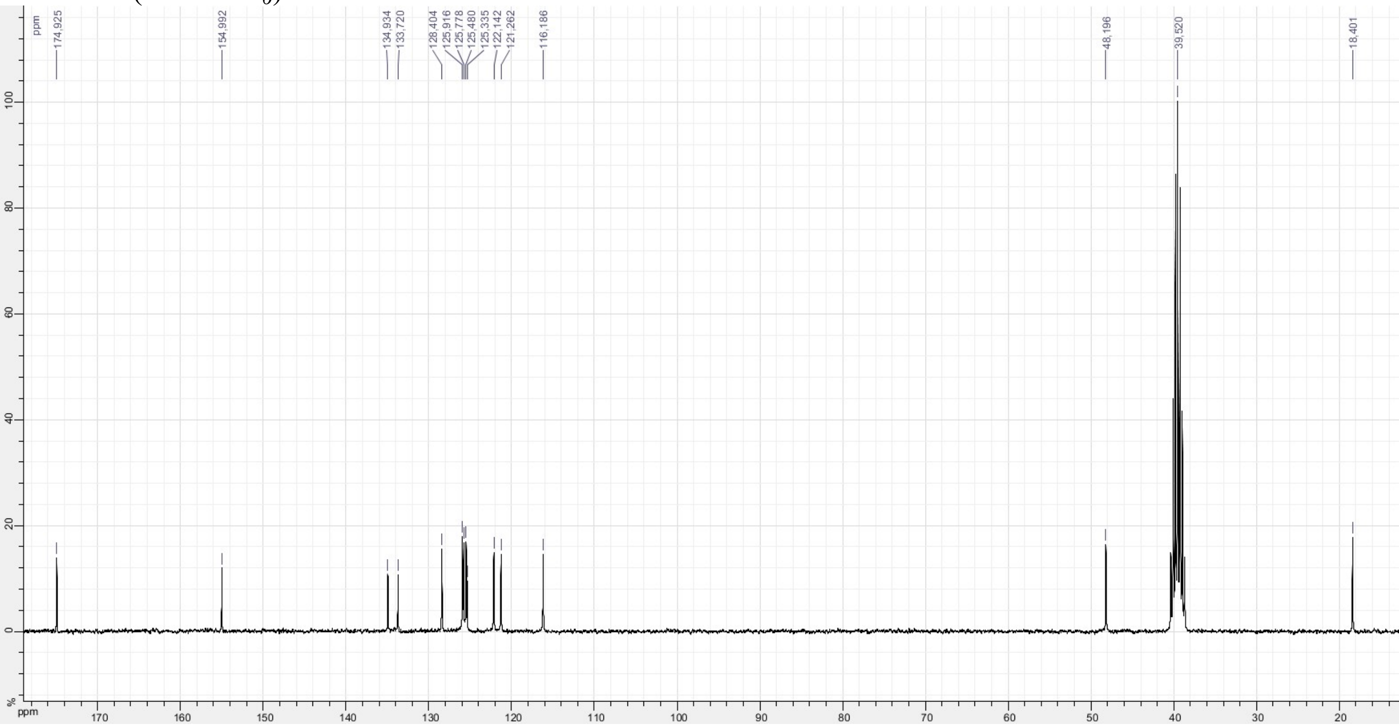

^{13}C (Dept135) NMR spectrum of **2a** in DMSO-d_6 at 75 MHz

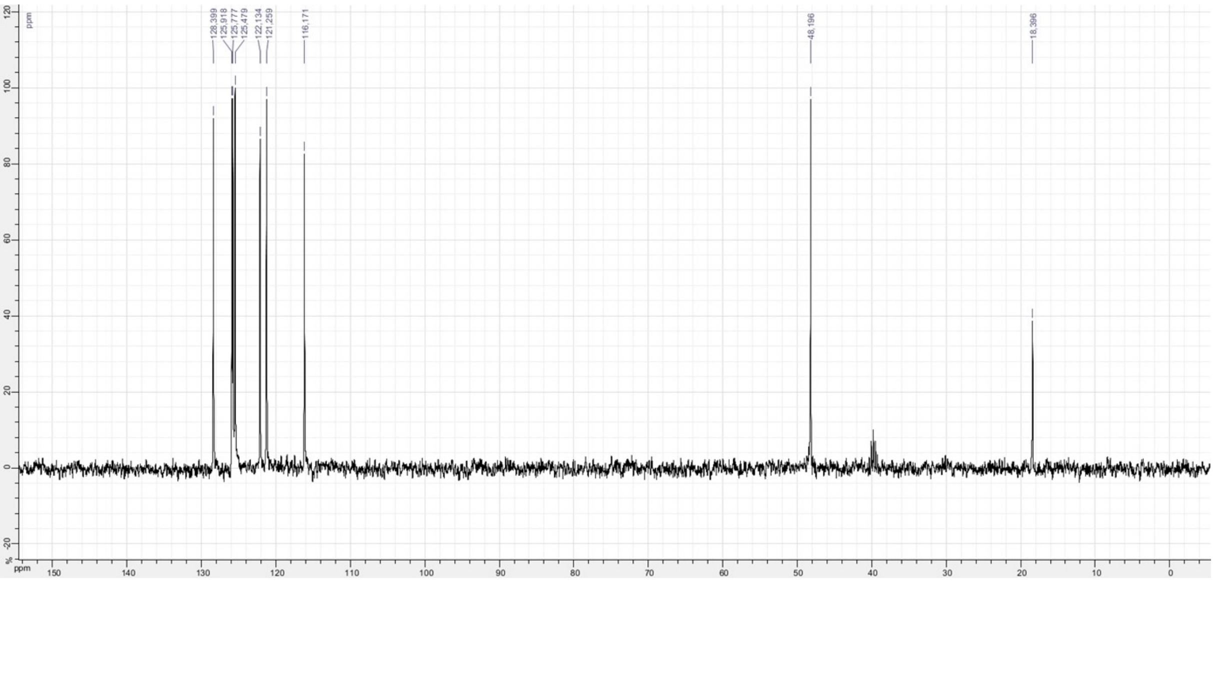

COSY NMR spectrum of **2a** in DMSO-d_6

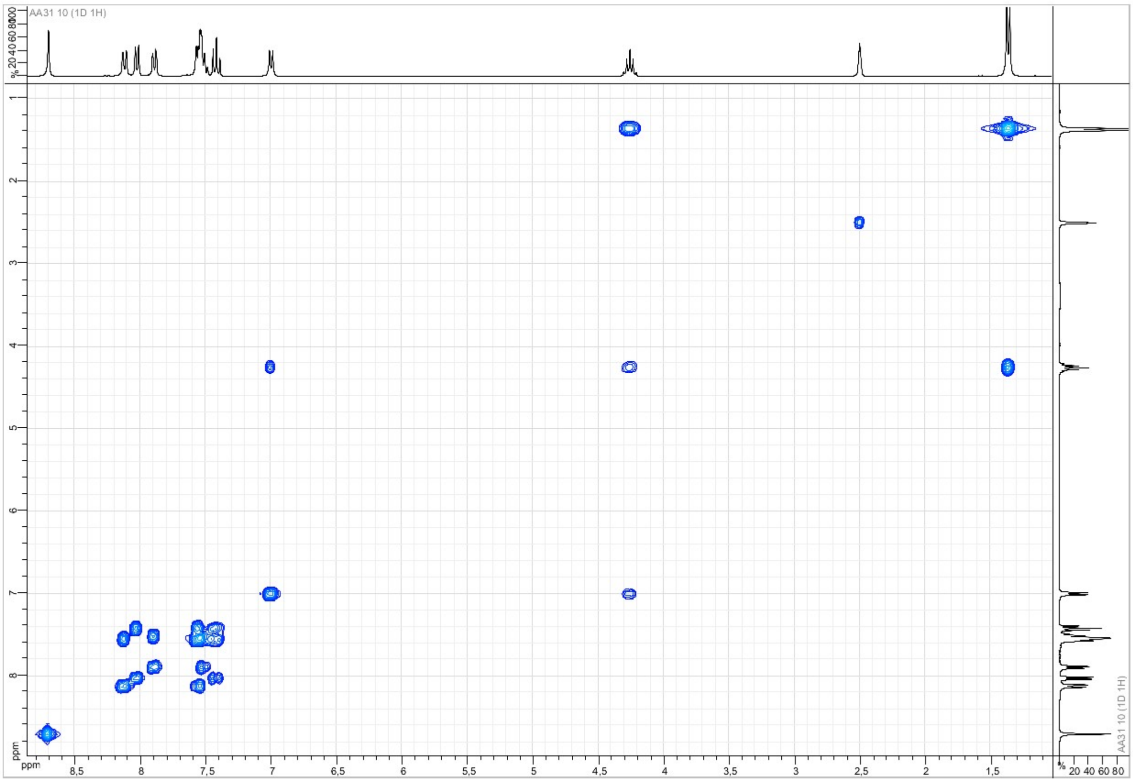

HSQC NMR spectrum of **2a** in DMSO-d_6

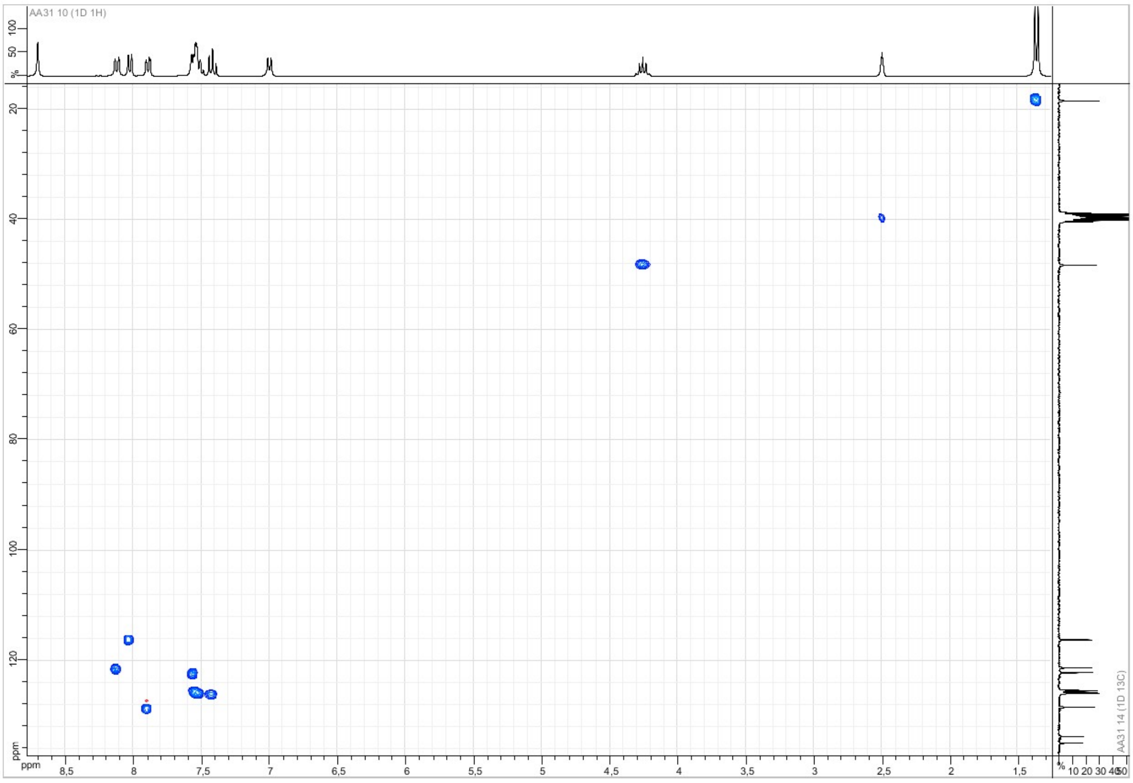

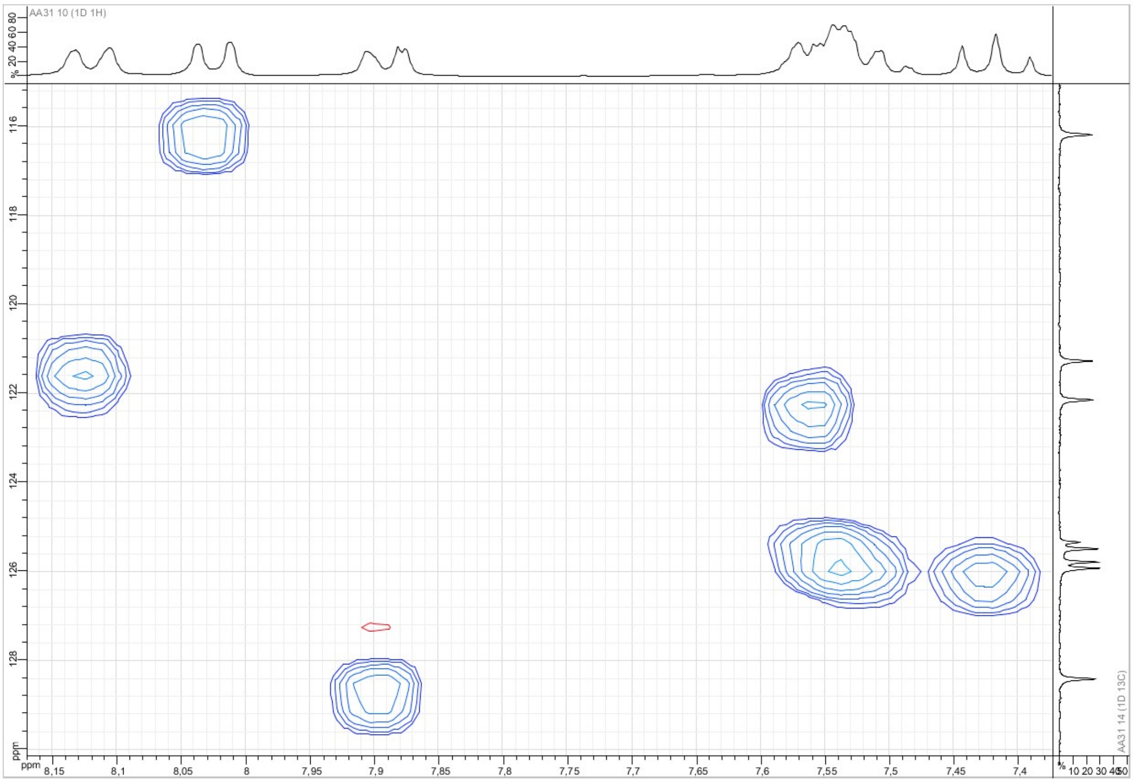

HMBC NMR spectrum of **2a** in DMSO-d_6

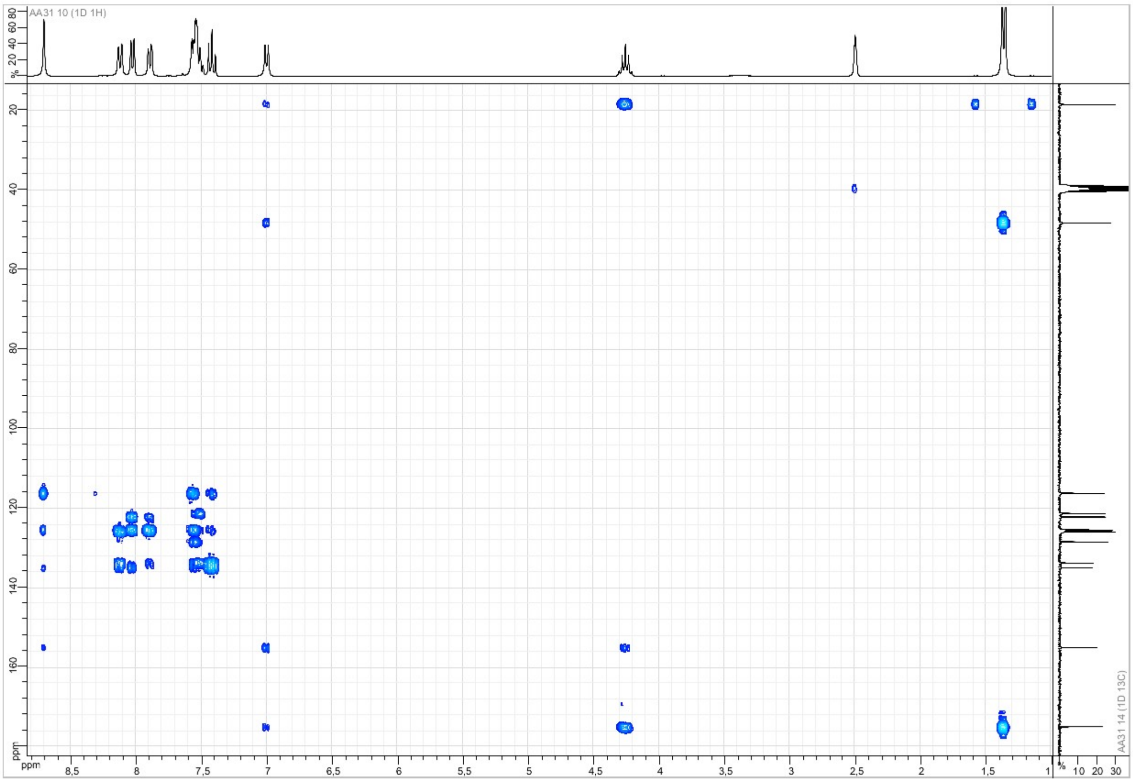

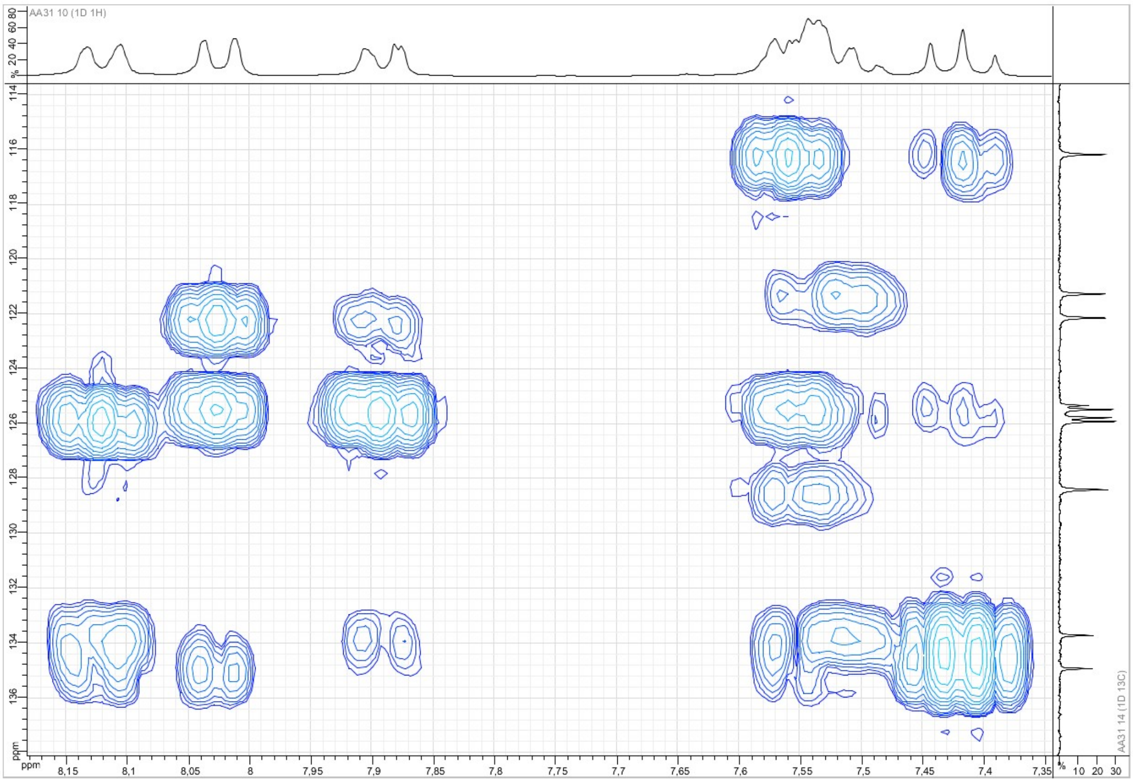

10. NMR spectra of 2b

^{1}H NMR (DMSO-d_6) of **2b** at 300 MHz

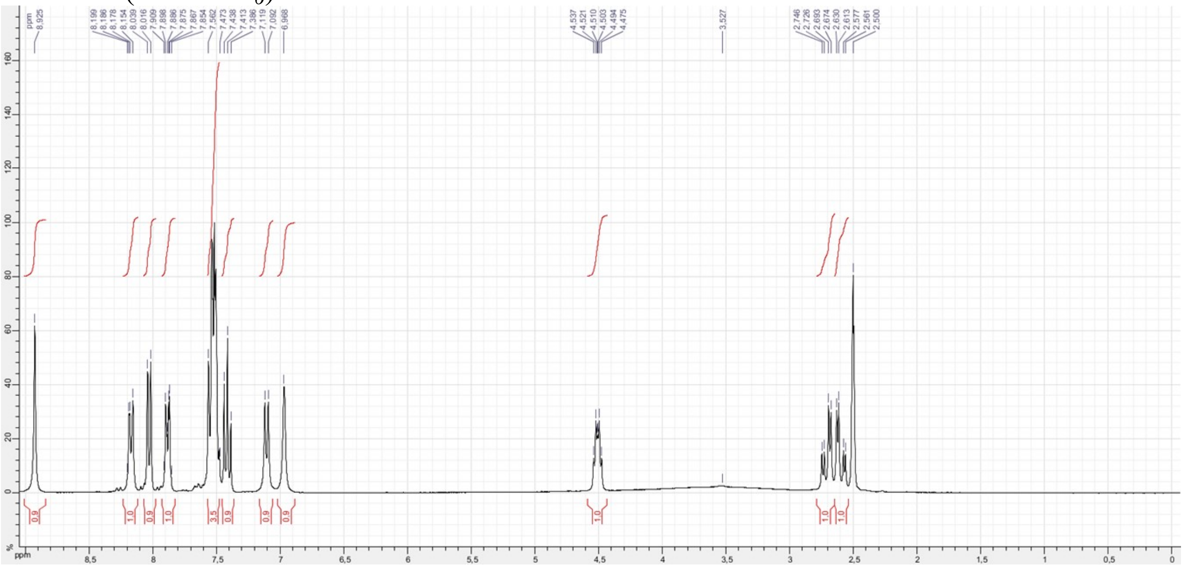

^{13}C NMR (DMSO-d_6) of **2b** at 75 MHz

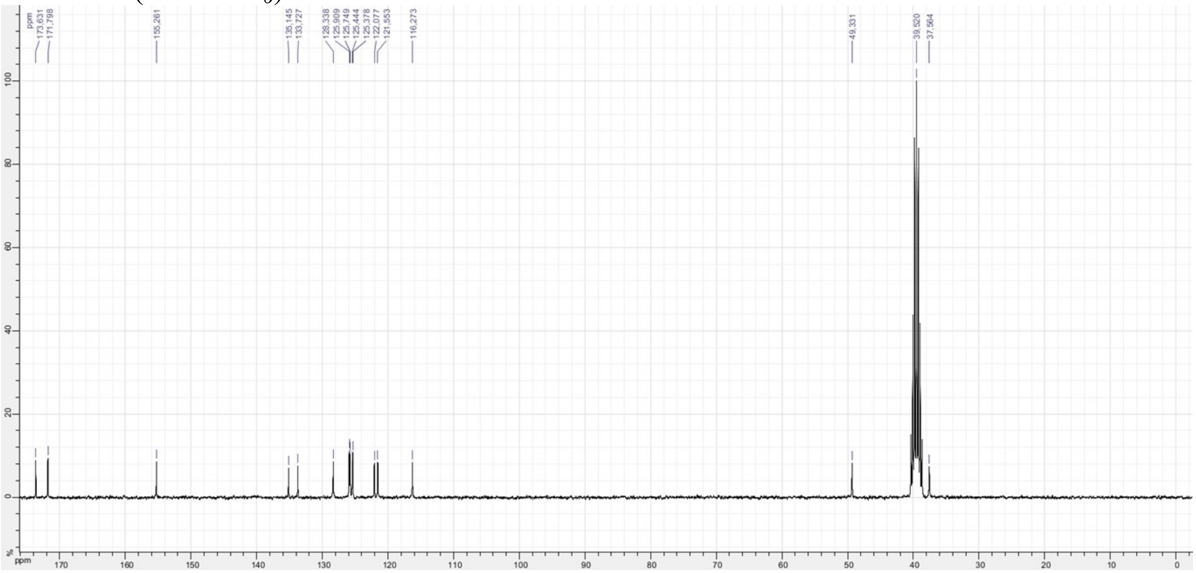

^{13}C (Dept135) NMR spectrum of **2b** in DMSO-d_6 at 75 MHz

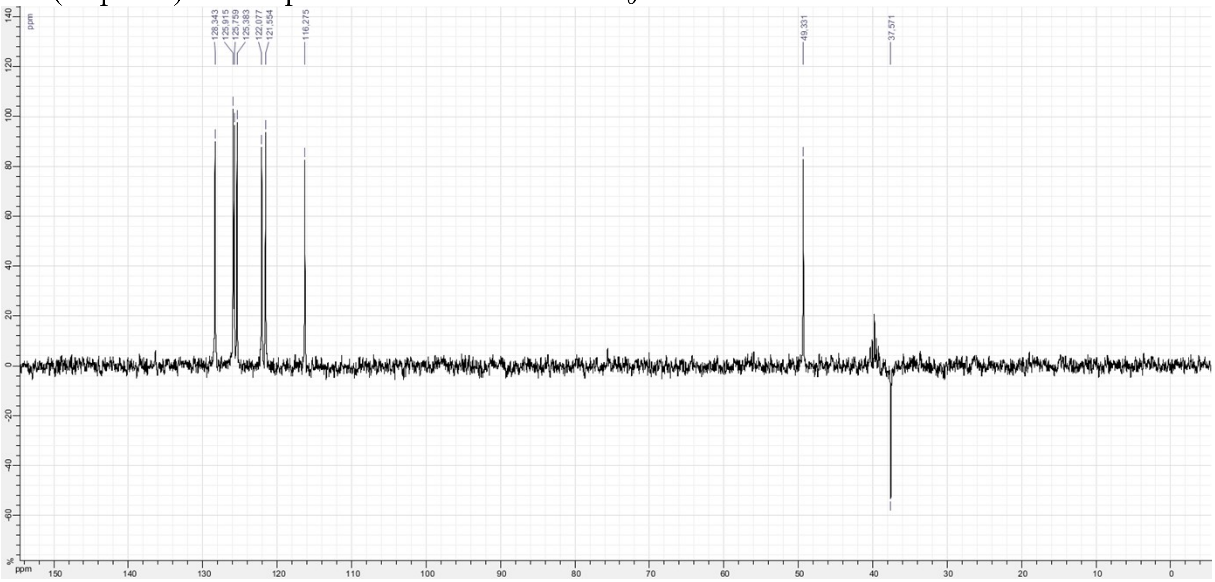

COSY NMR spectrum of **2b** in DMSO-d_6

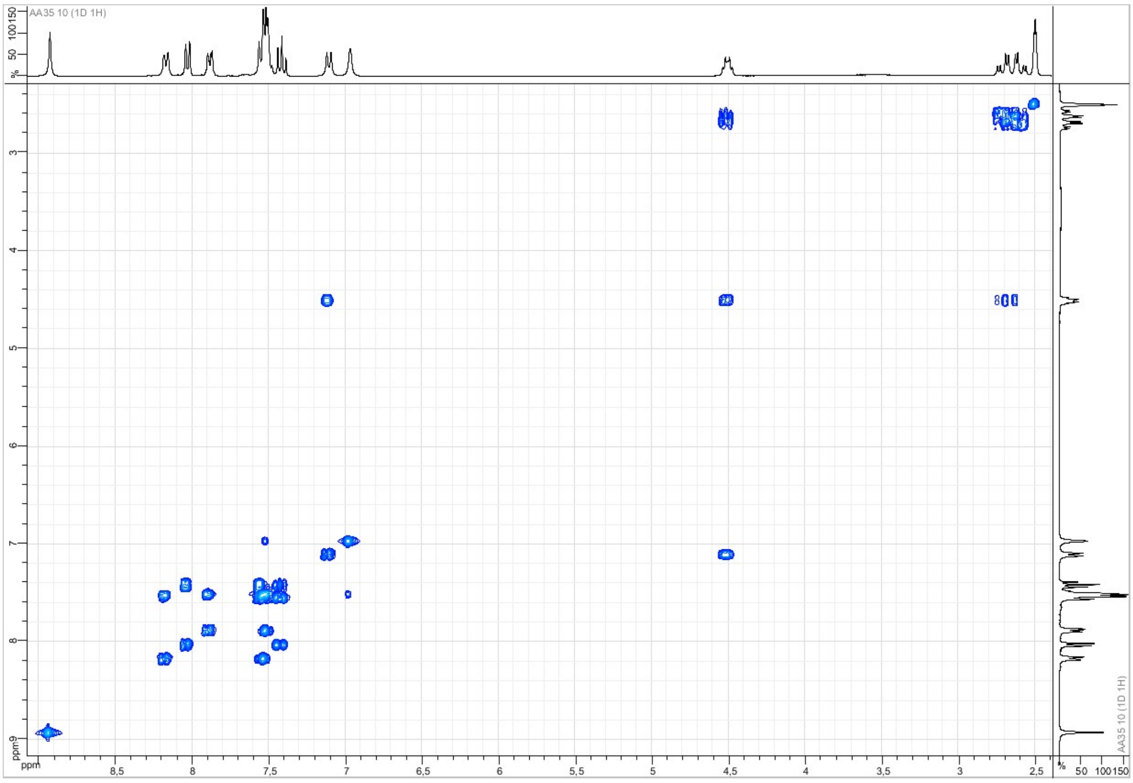

HSQC NMR spectrum of **2b** in DMSO-d_6

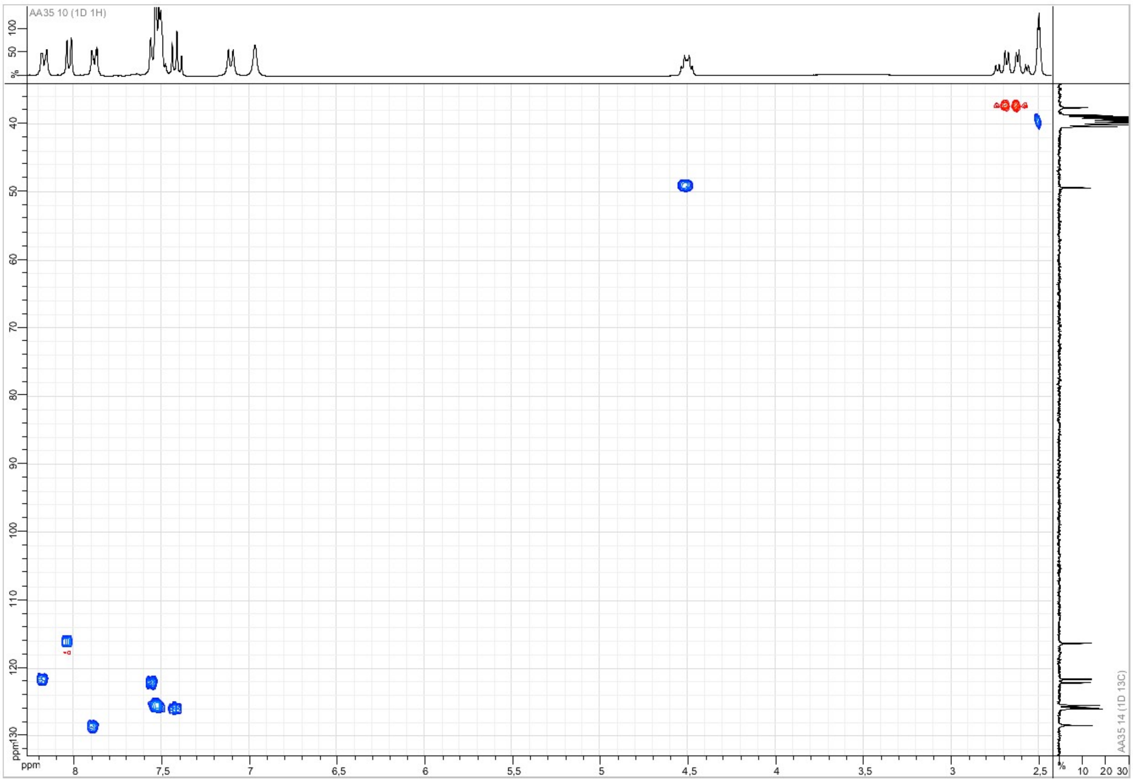

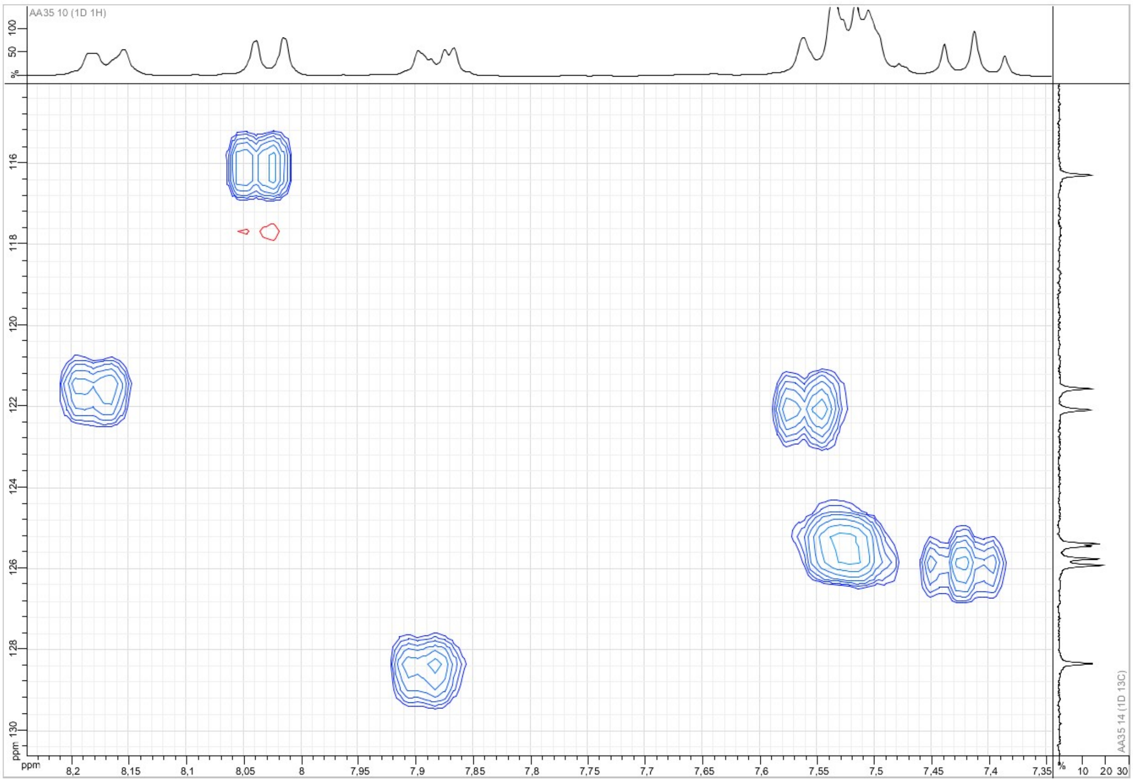

11. NMR spectra of 2c

^{1}H NMR (DMSO-d_6) of **2c** at 300 MHz

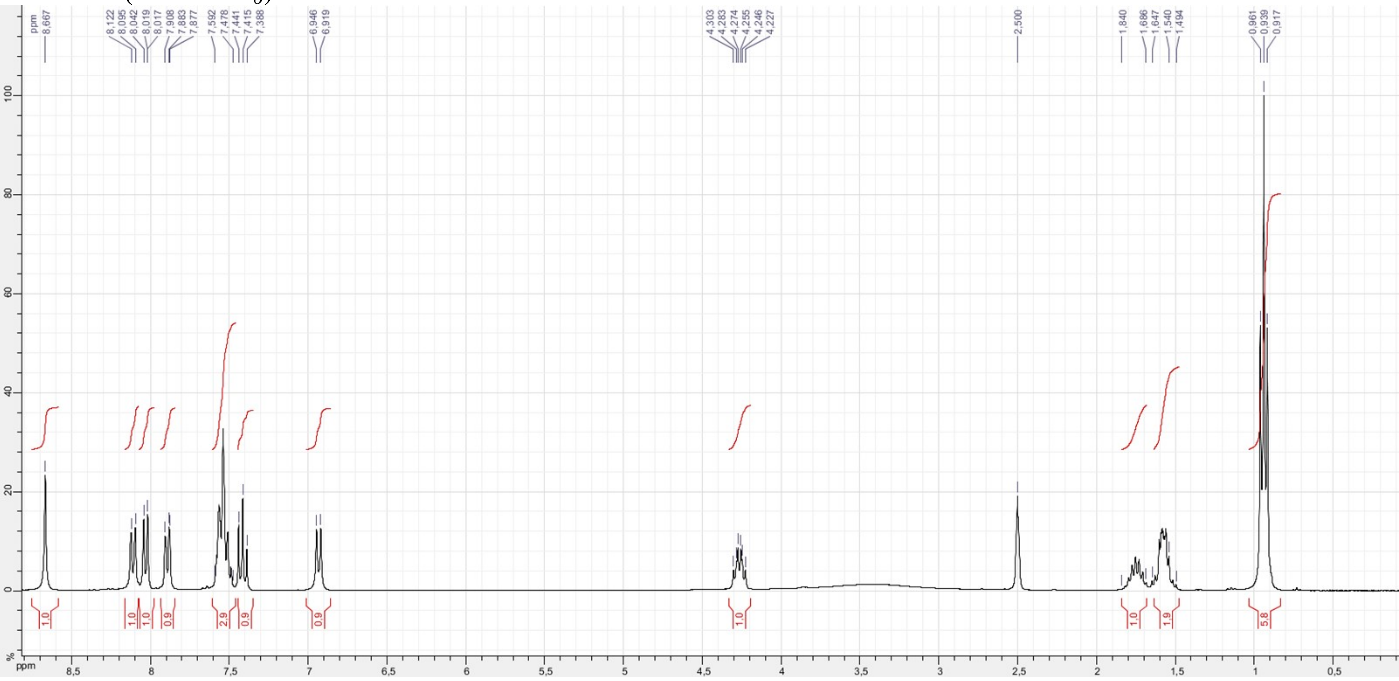

^{13}C NMR (DMSO-d_6) of **2c** at 75 MHz

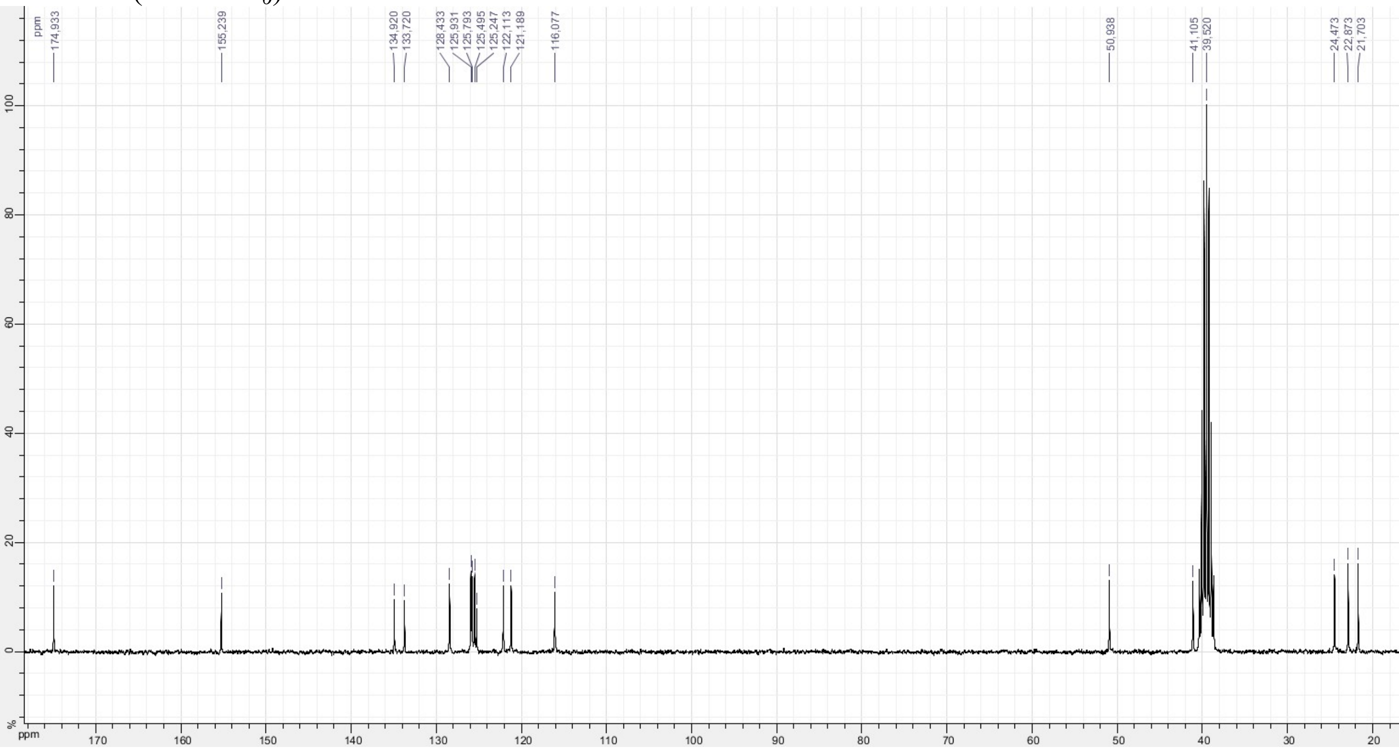

^{13}C (Dept135) NMR spectrum of **2c** in DMSO-d_6 at 75 MHz

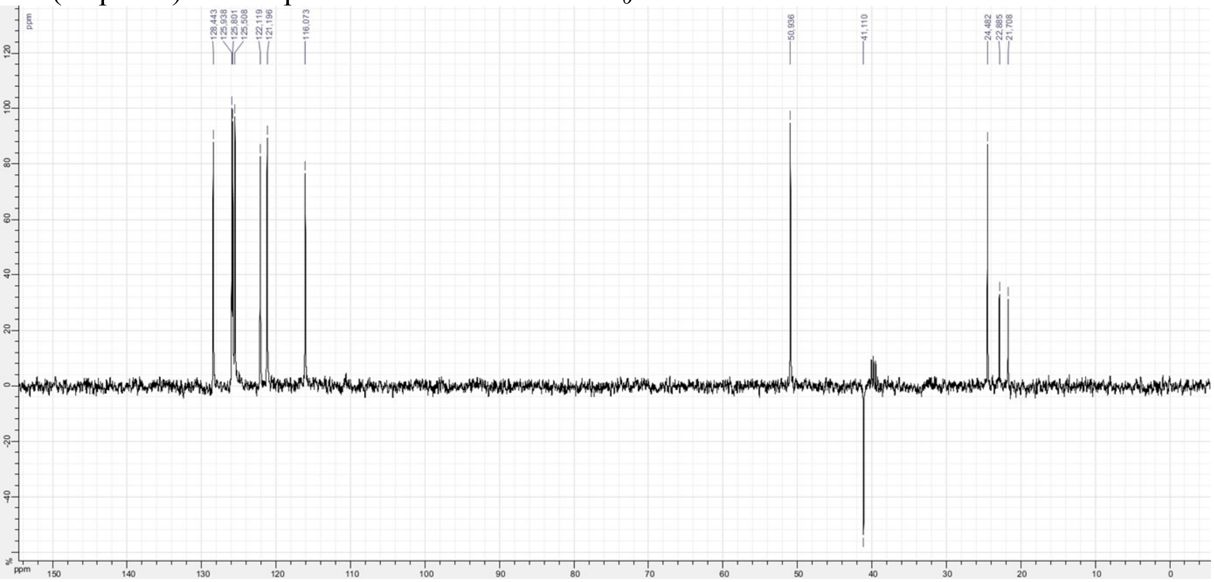

COSY NMR spectrum of **2c** in DMSO-d_6

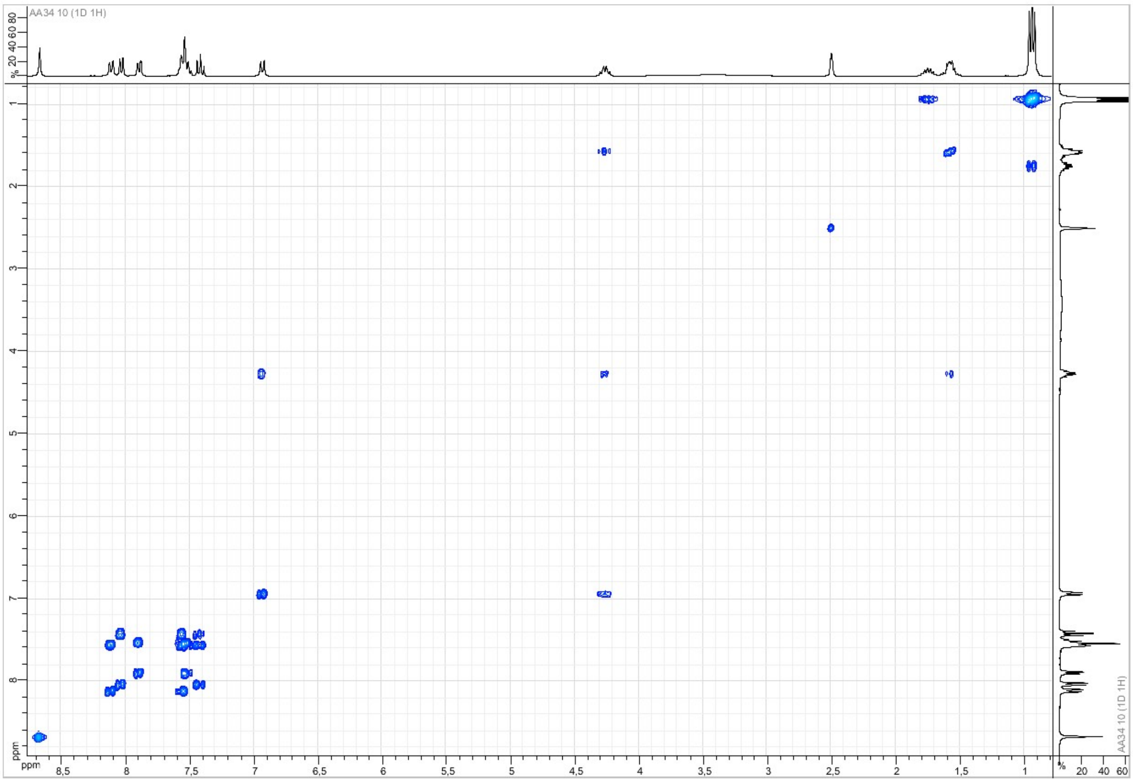

HSQC NMR spectrum of **2c** in DMSO-d_6

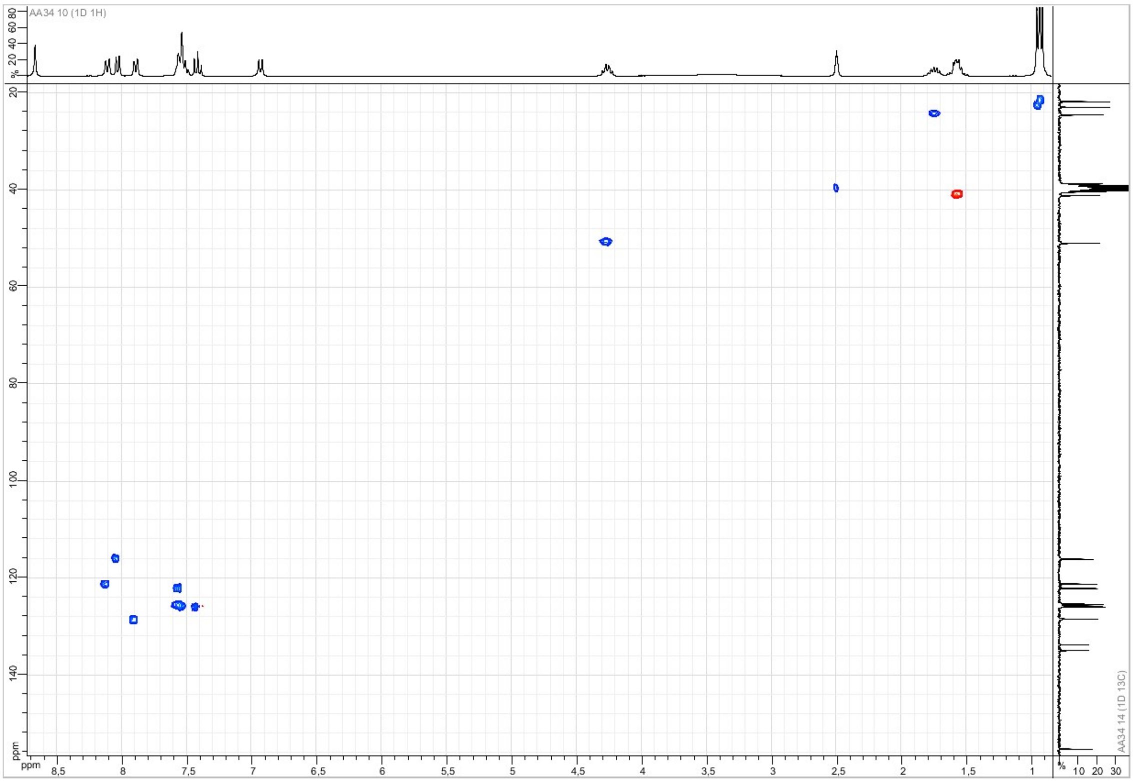

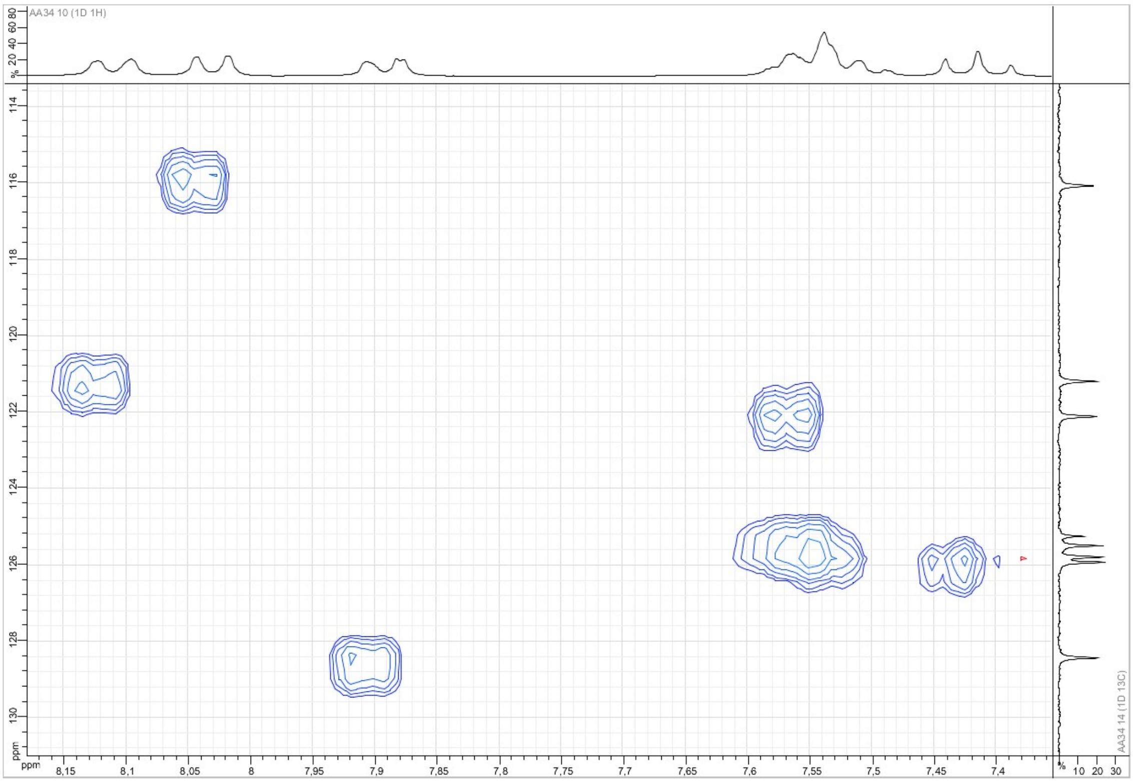

12. NMR spectra of 2d

^{1}H NMR (DMSO-d_6) of **2d** at 300 MHz

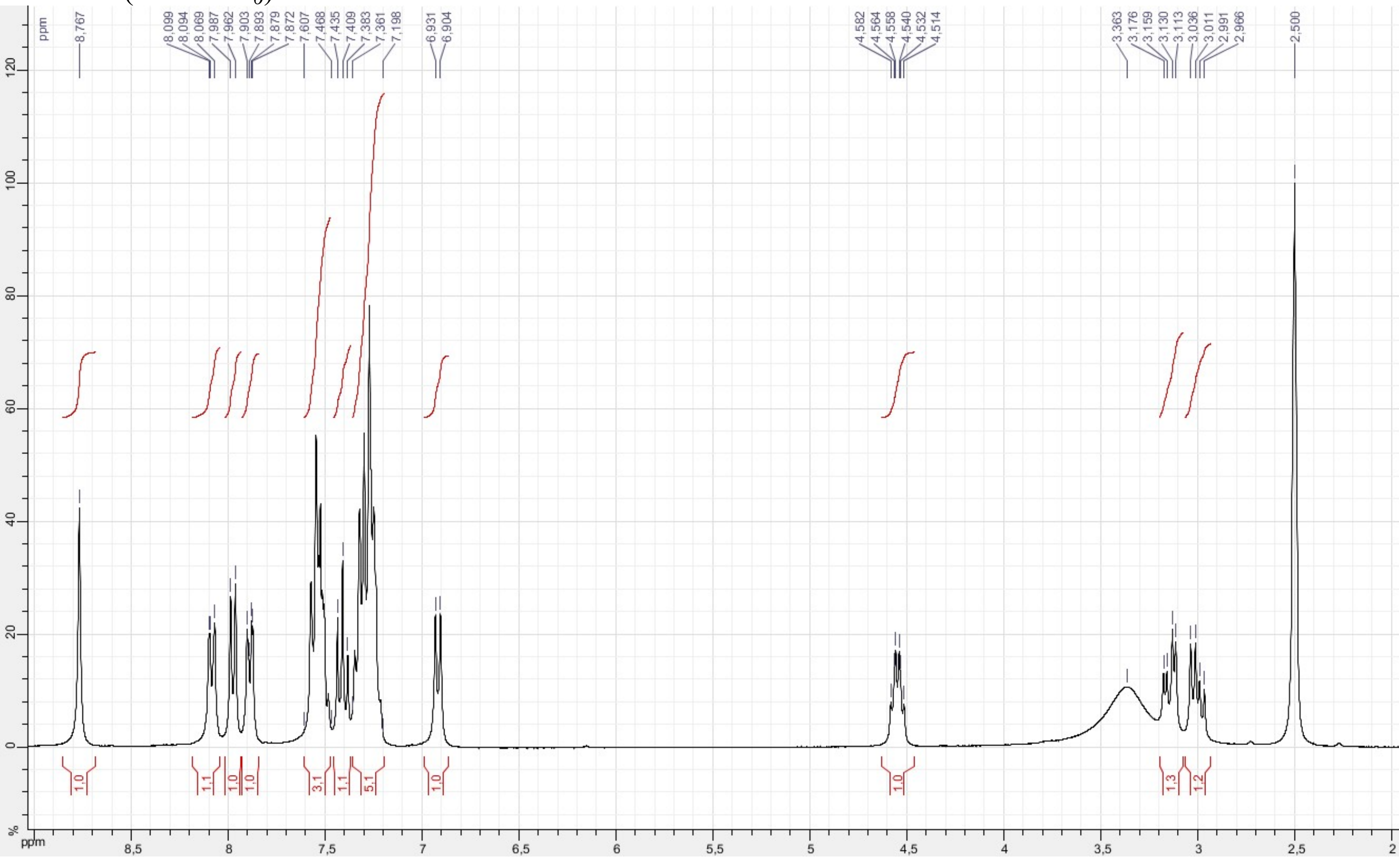

^{13}C NMR (DMSO-d_6) of **2d** at 75 MHz

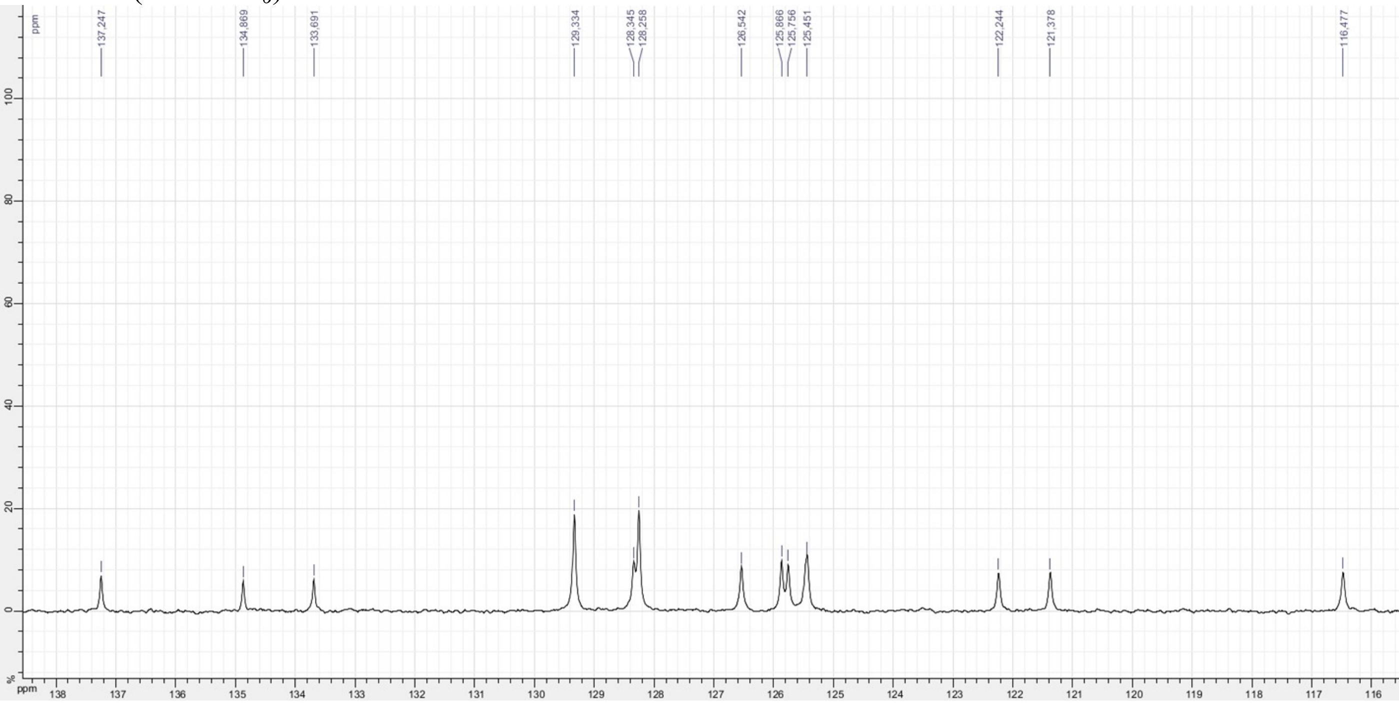

^{13}C (Dept135) NMR spectrum of **2d** in DMSO-d_6 at 75 MHz

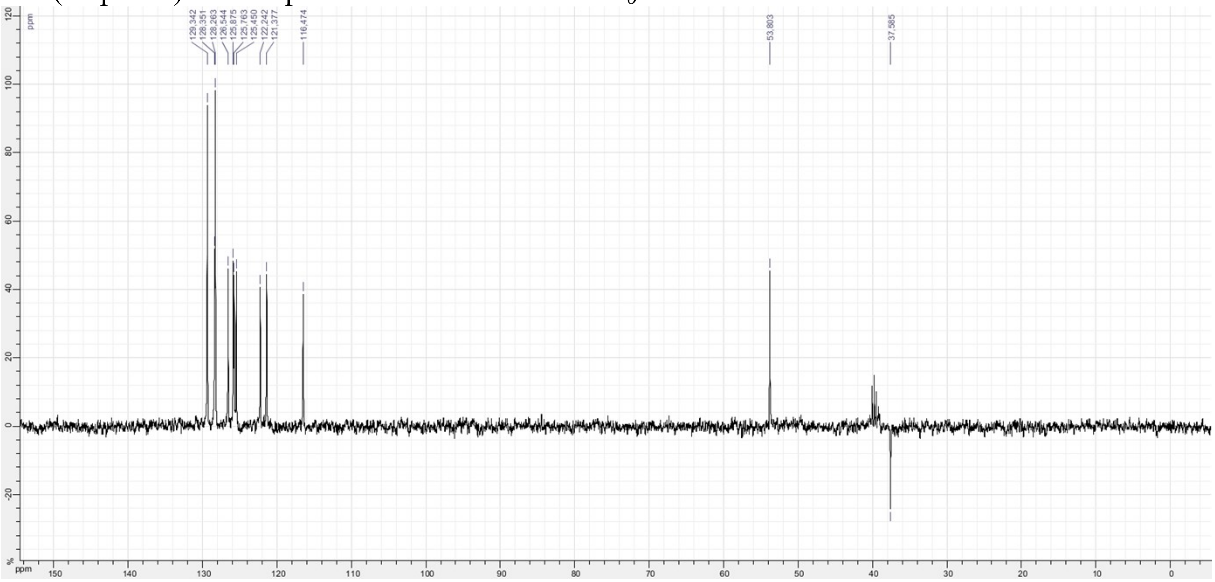

COSY NMR spectrum of **2d** in DMSO-d_6

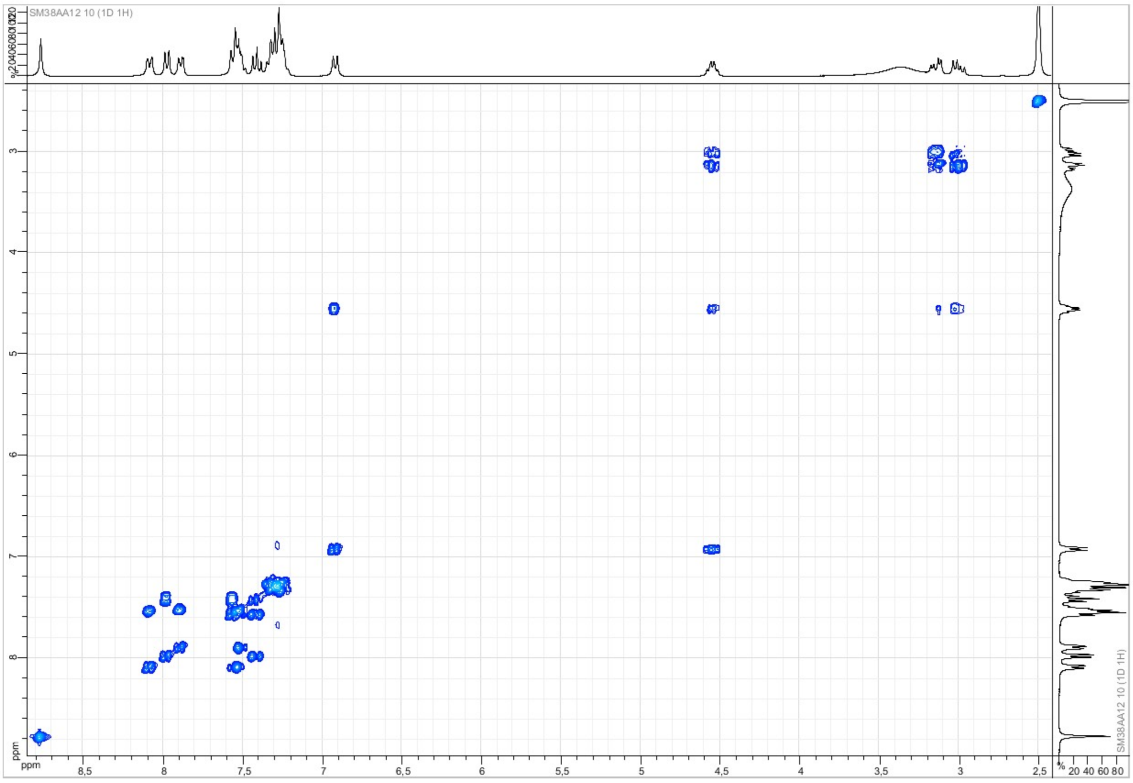

HSQC NMR spectrum of **2d** in DMSO-d_6

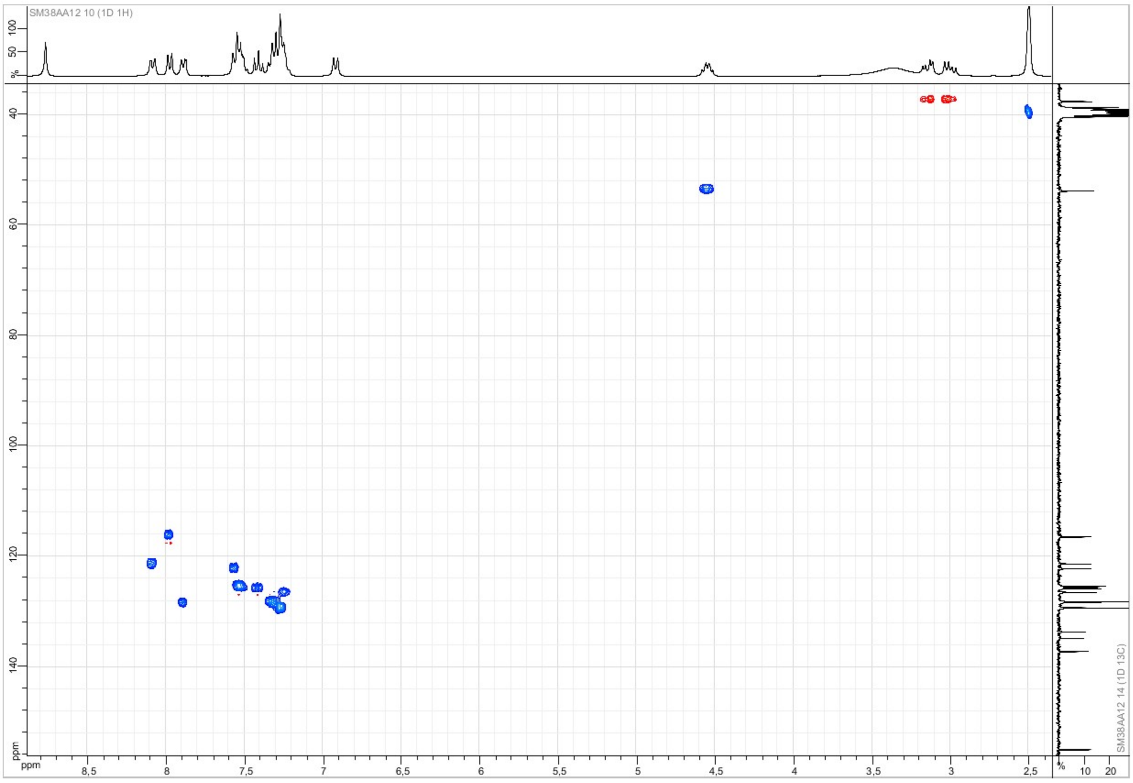

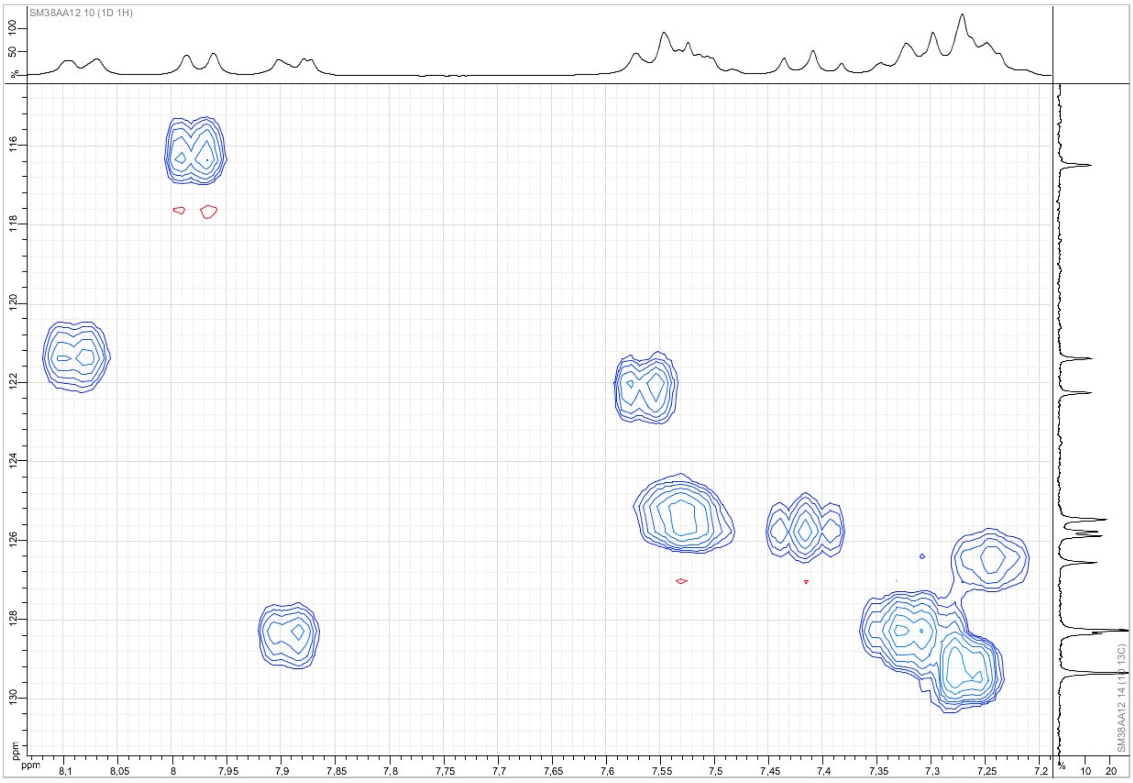

HMBC NMR spectrum of **2d** in DMSO-d_6

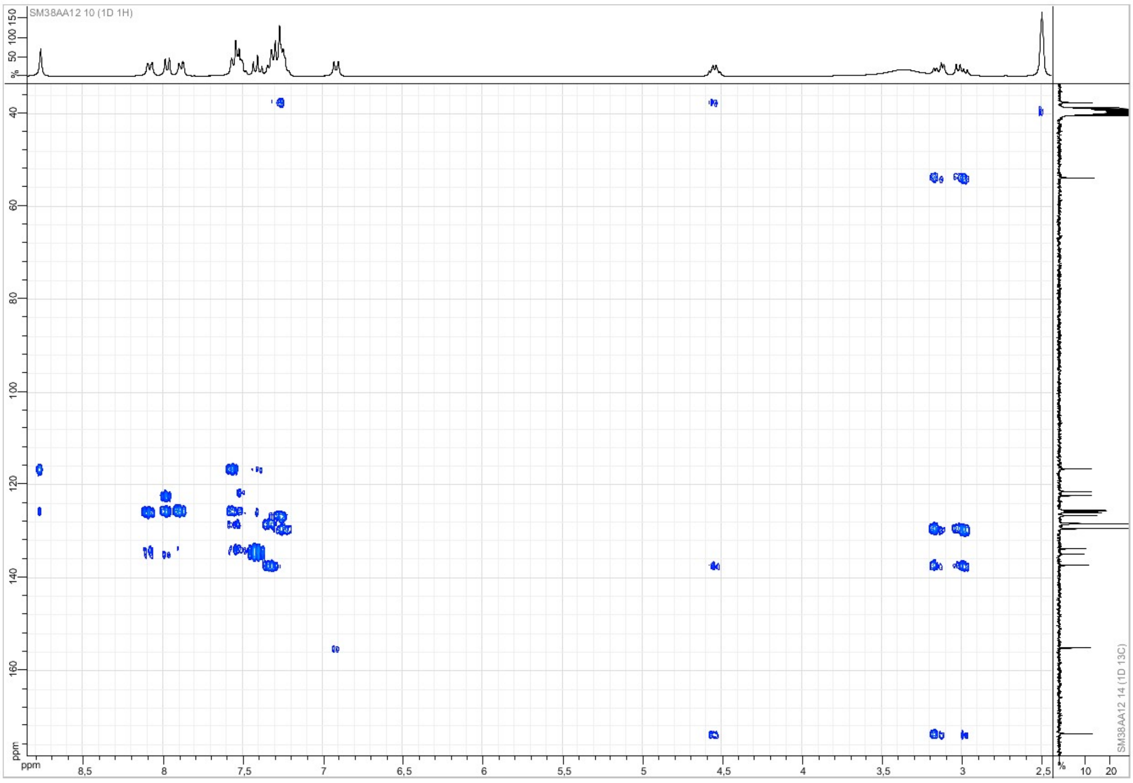

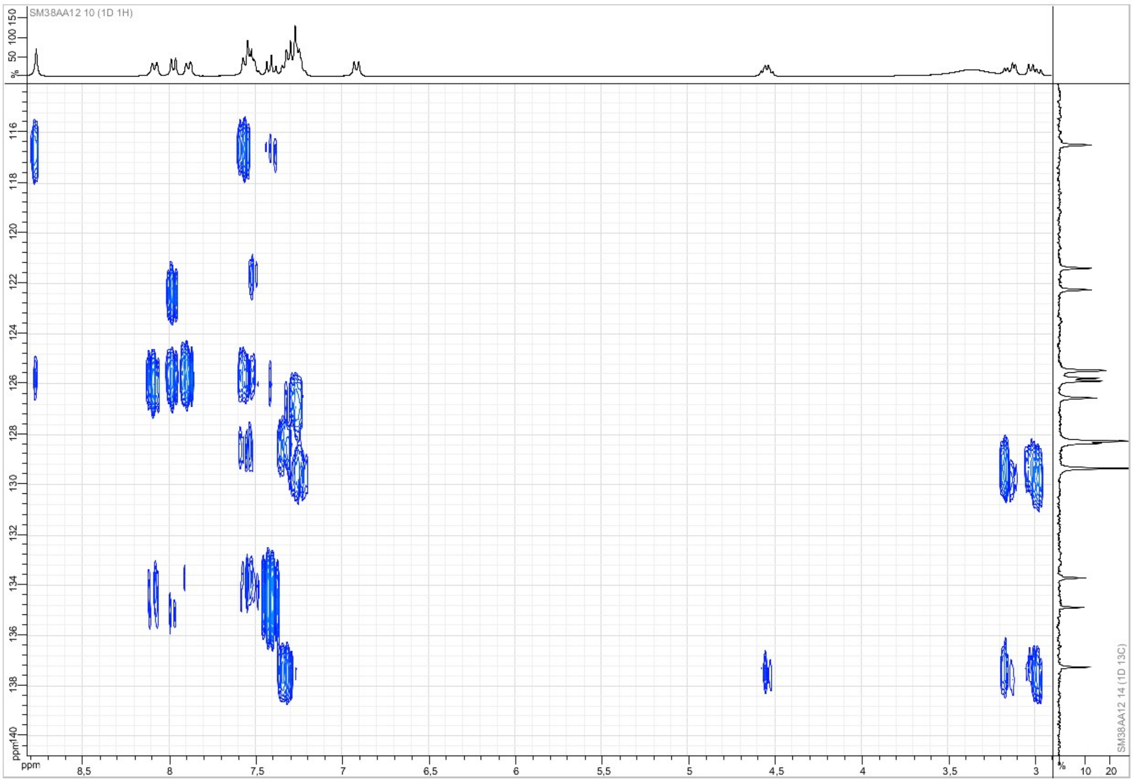

13. NMR spectra of 2e

^{1}H NMR (DMSO-d_6) of **2e** at 300 MHz

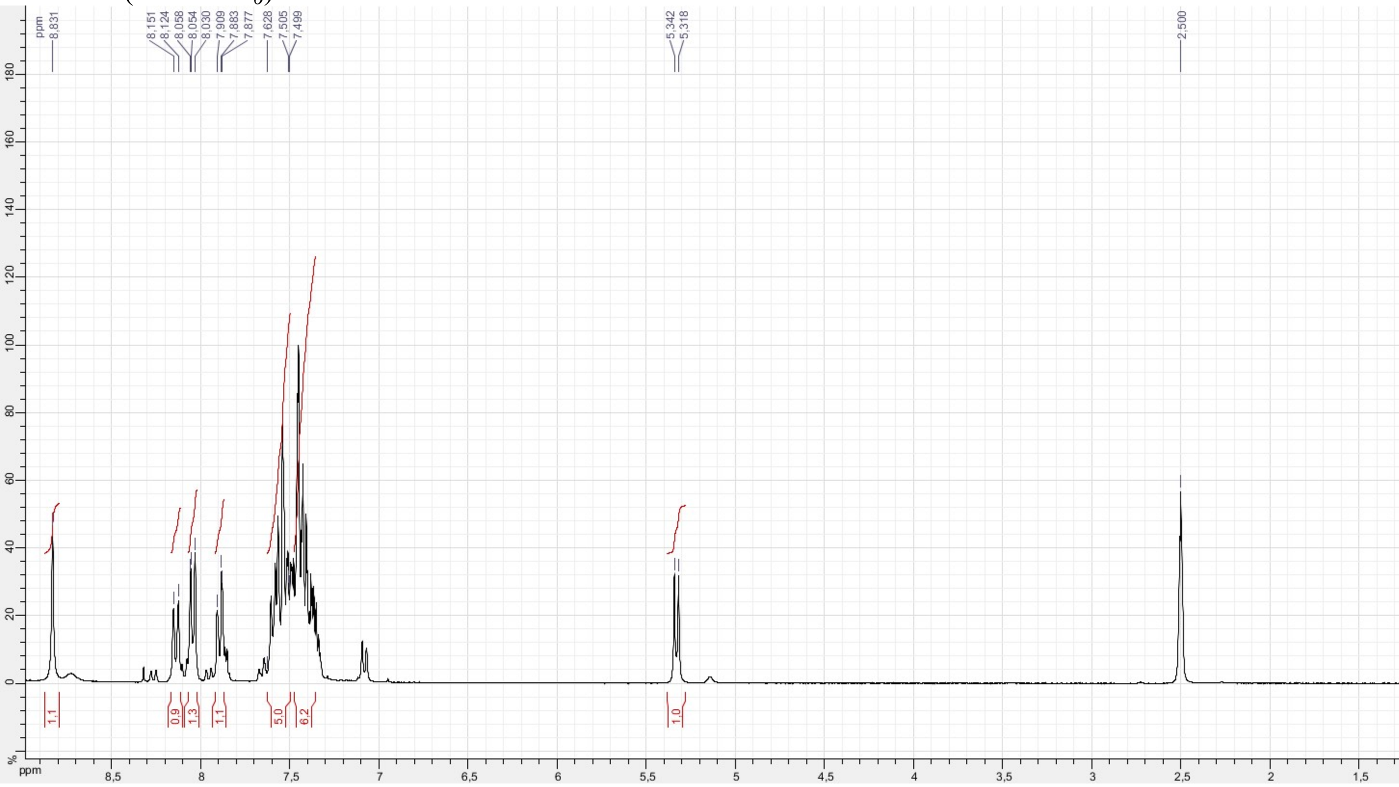

^{13}C NMR (DMSO-d_6) of **2e** at 75 MHz

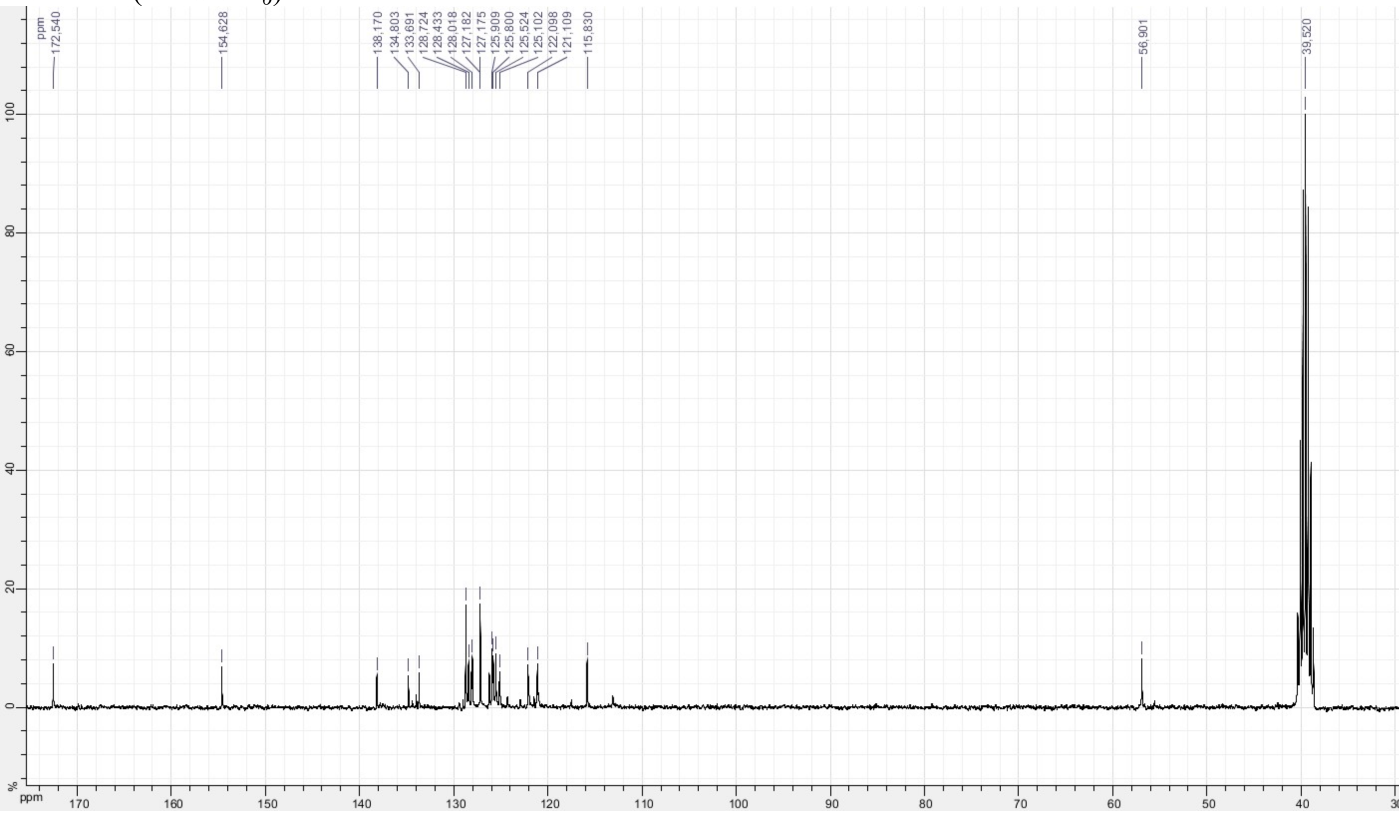

^{13}C (Dept135) NMR spectrum of **2e** in DMSO-d_6 at 75 MHz

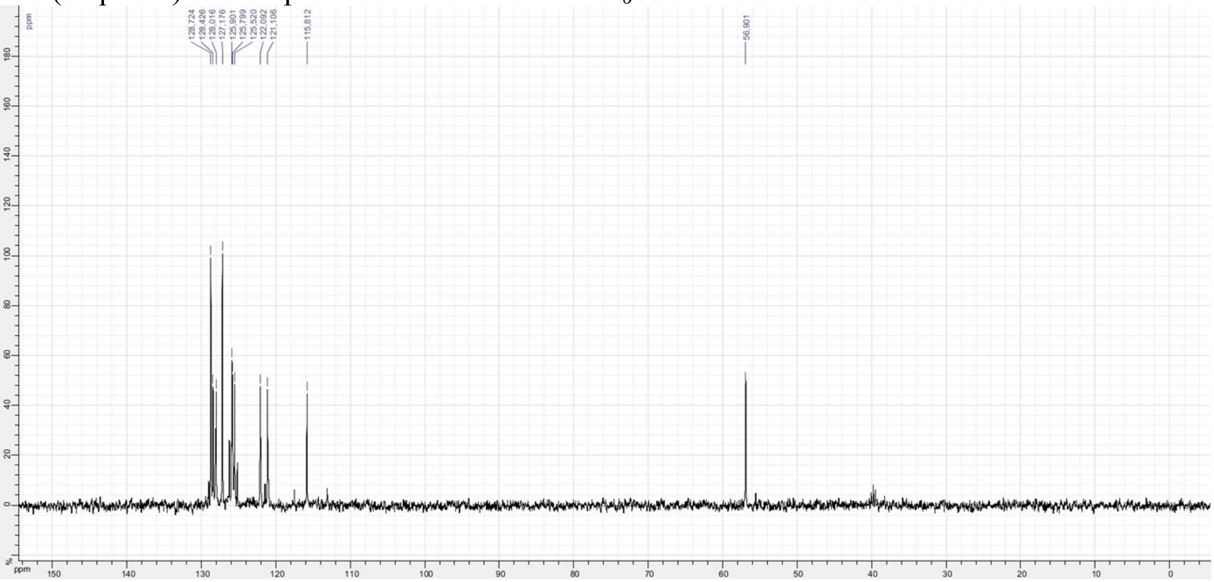

COSY NMR spectrum of **2e** in DMSO-d_6

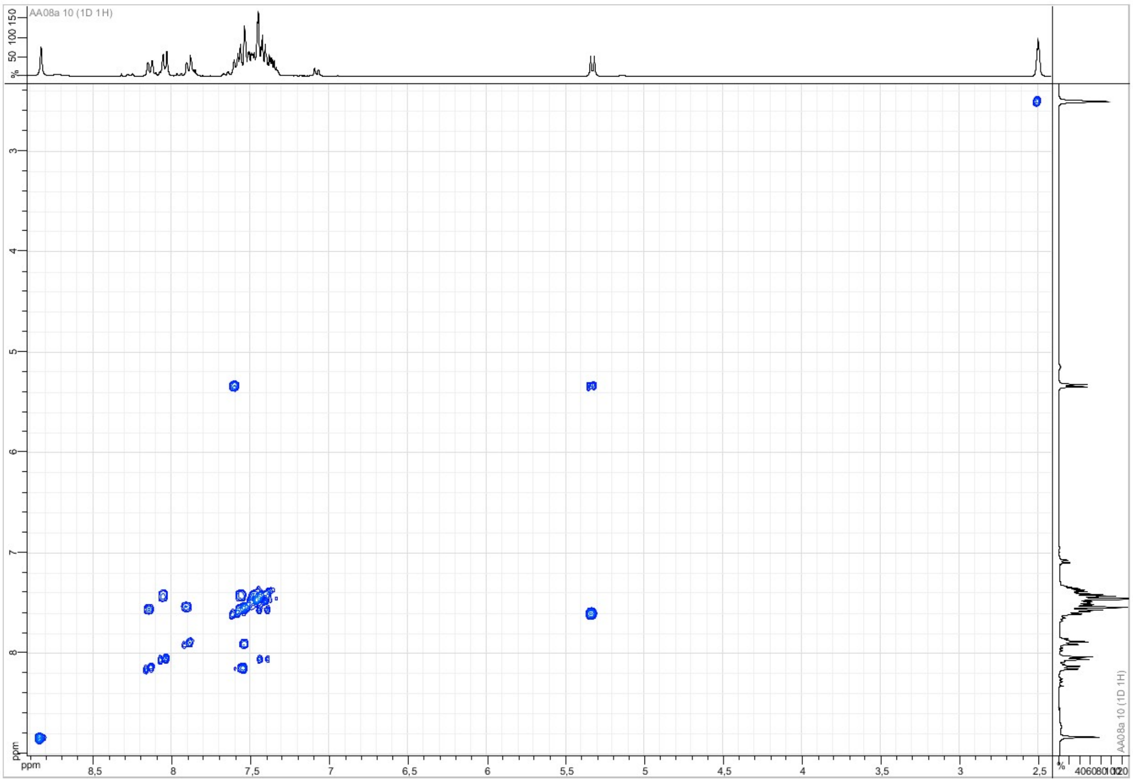

HSQC NMR spectrum of **2e** in DMSO-d_6

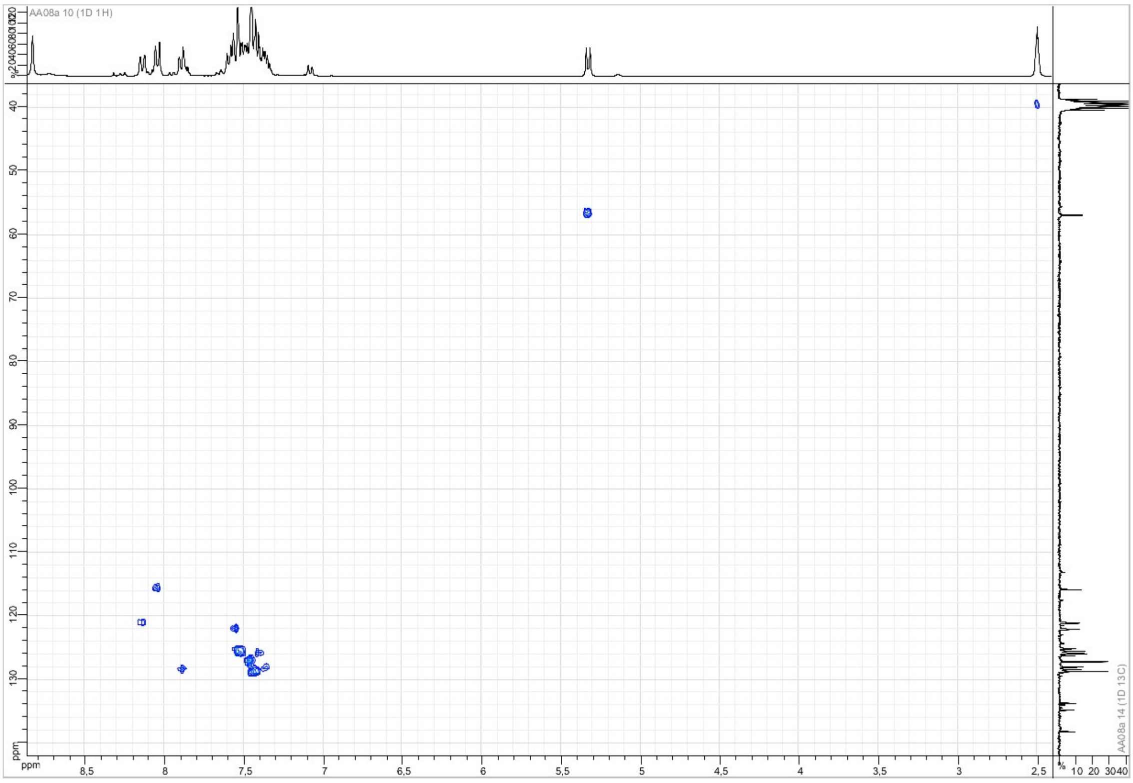

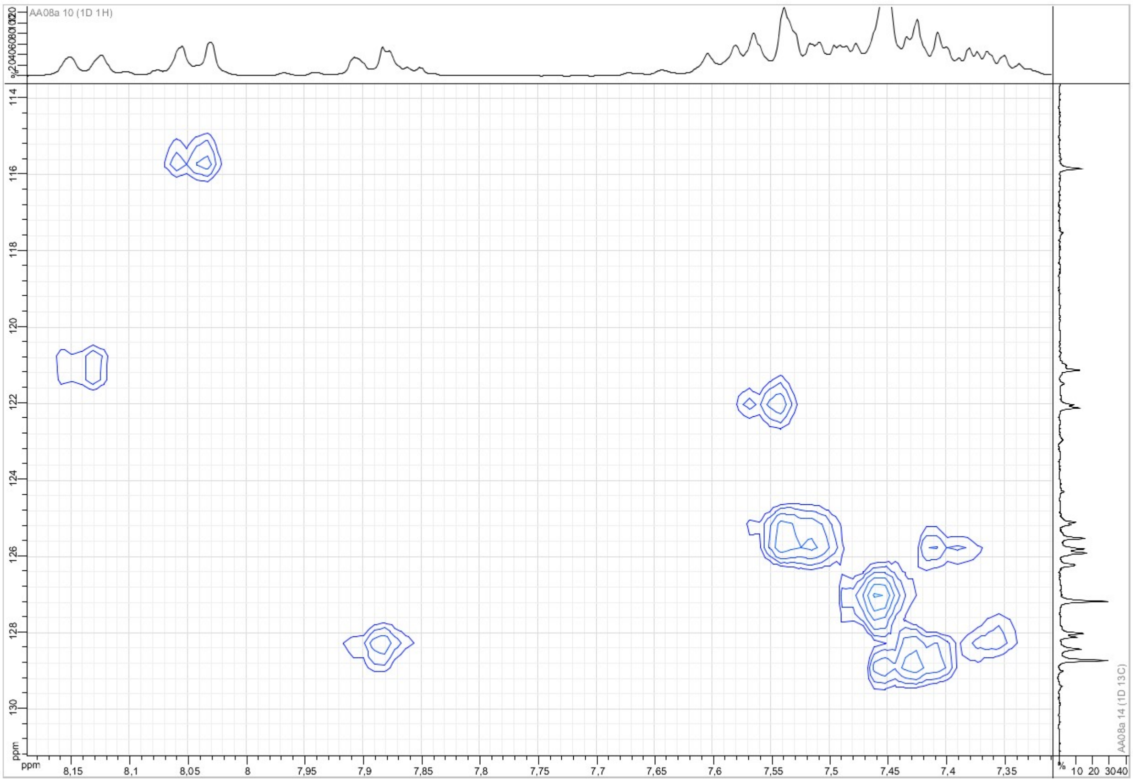

HMBC NMR spectrum of **2e** in DMSO-d_6

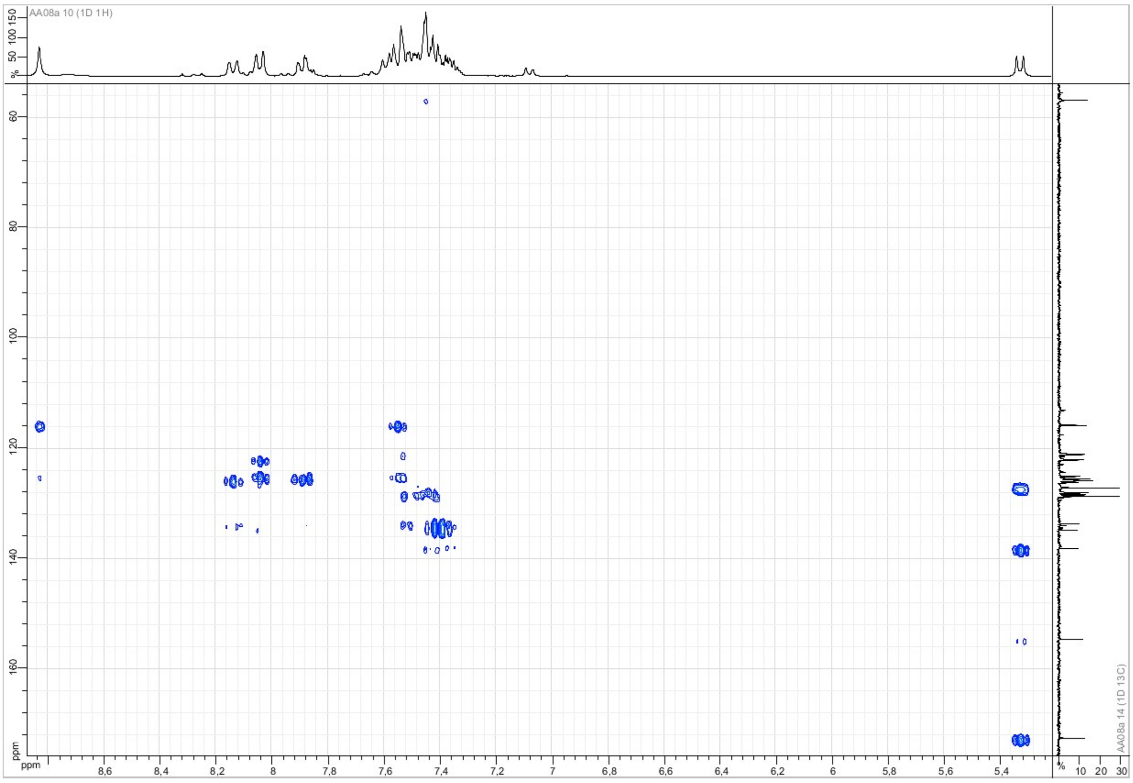

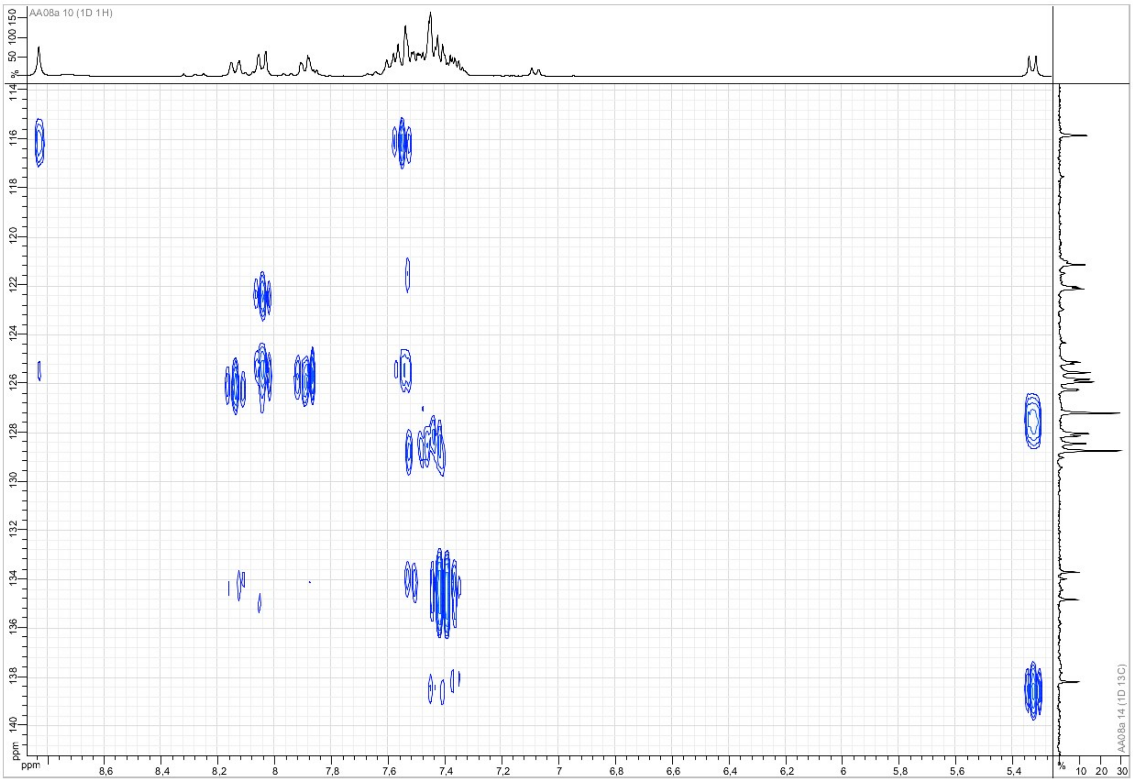

14. NMR spectra of 2f

^{1}H NMR (DMSO-d_6) of **2f** at 300 MHz

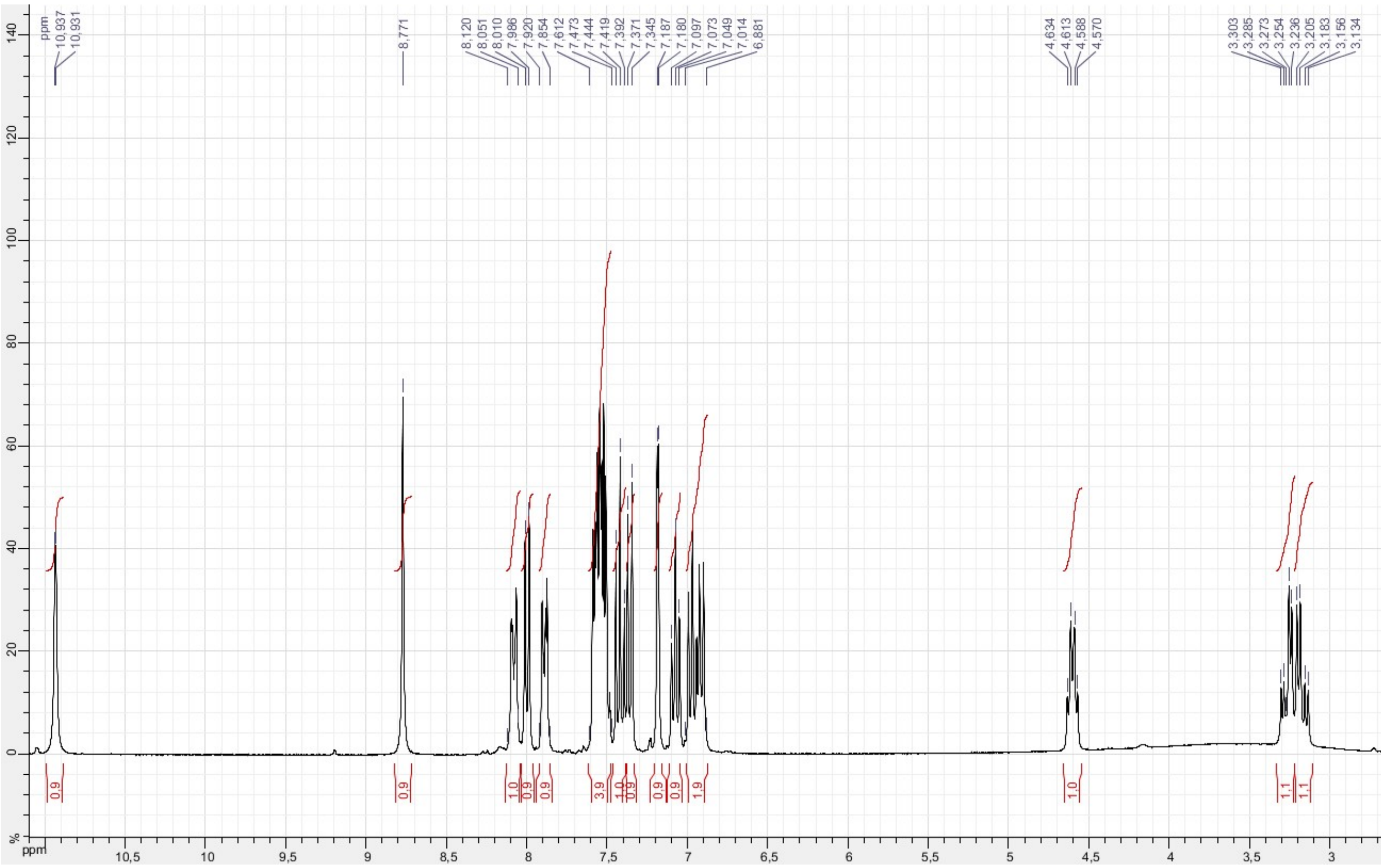

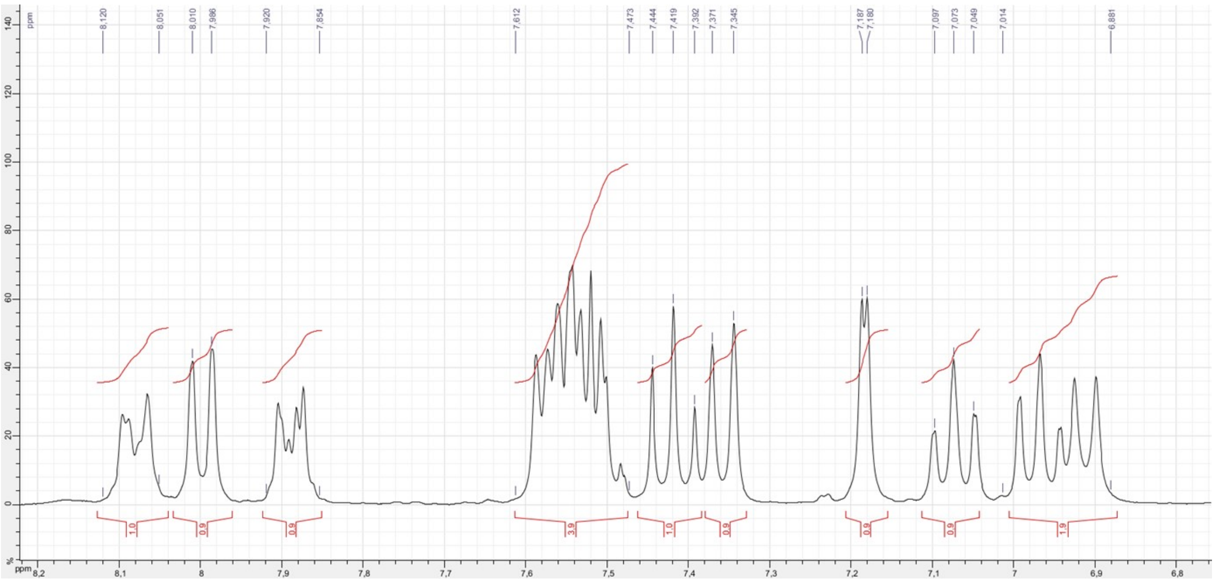

^{13}C NMR (DMSO-d_6) of **2f** at 75 MHz

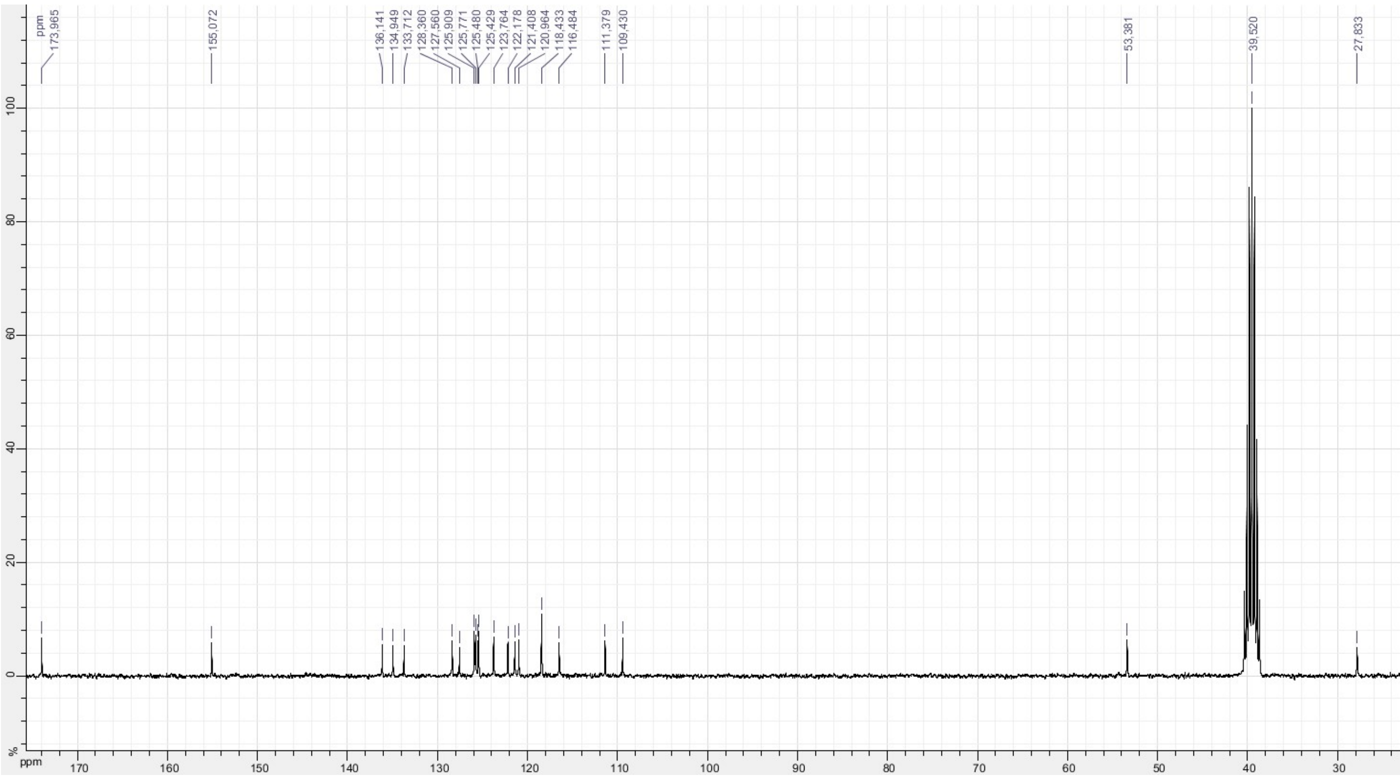

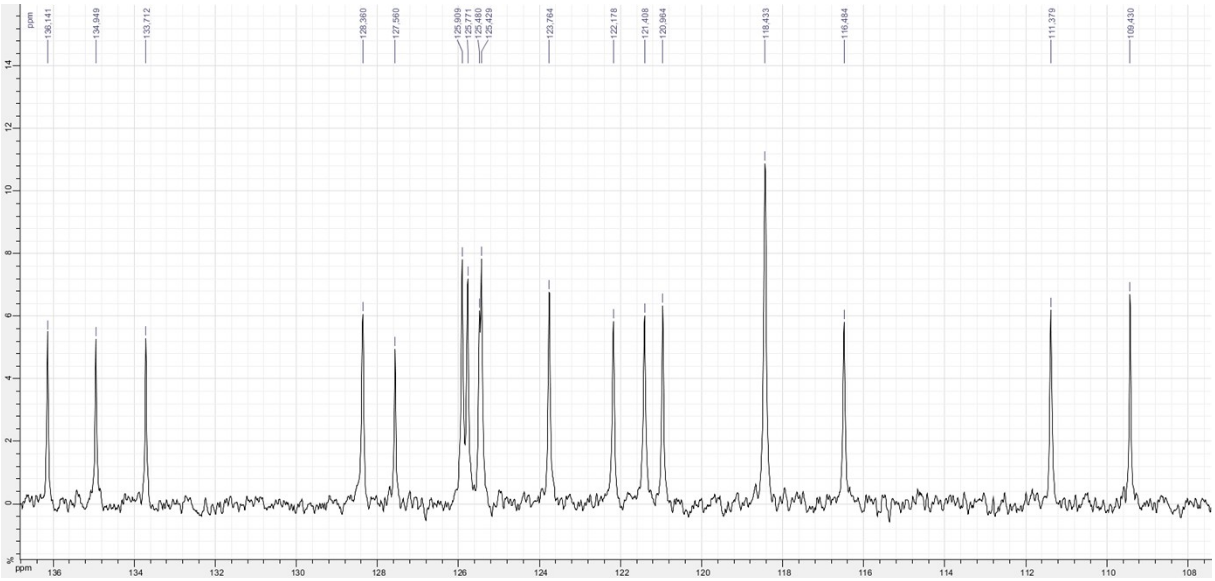

^{13}C (Dept135) NMR spectrum of **2f** in DMSO-d_6 at 75 MHz

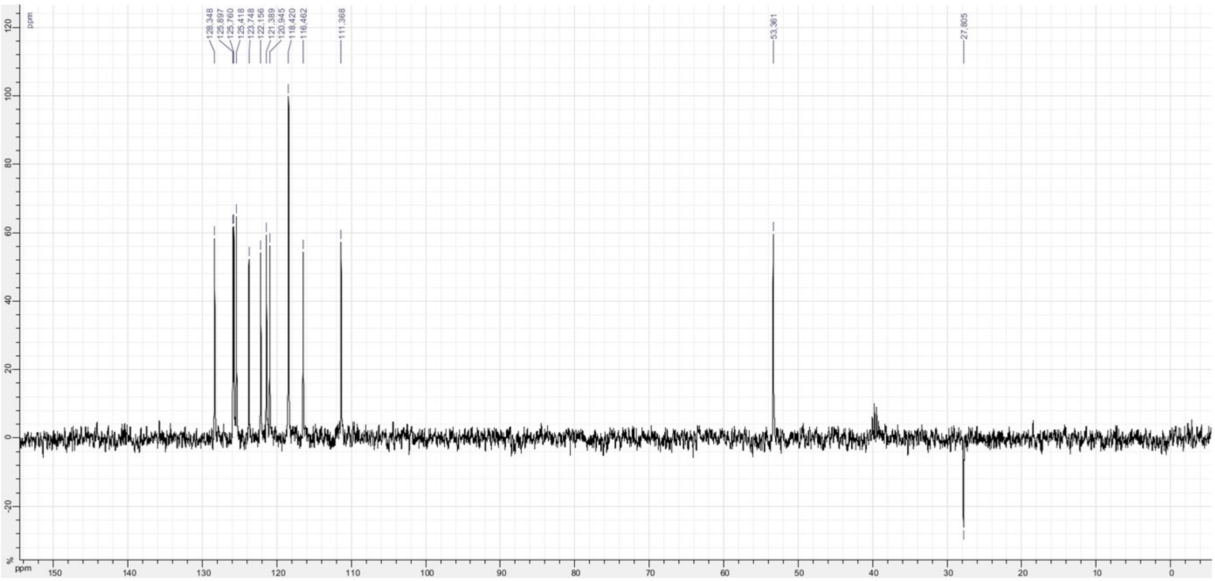

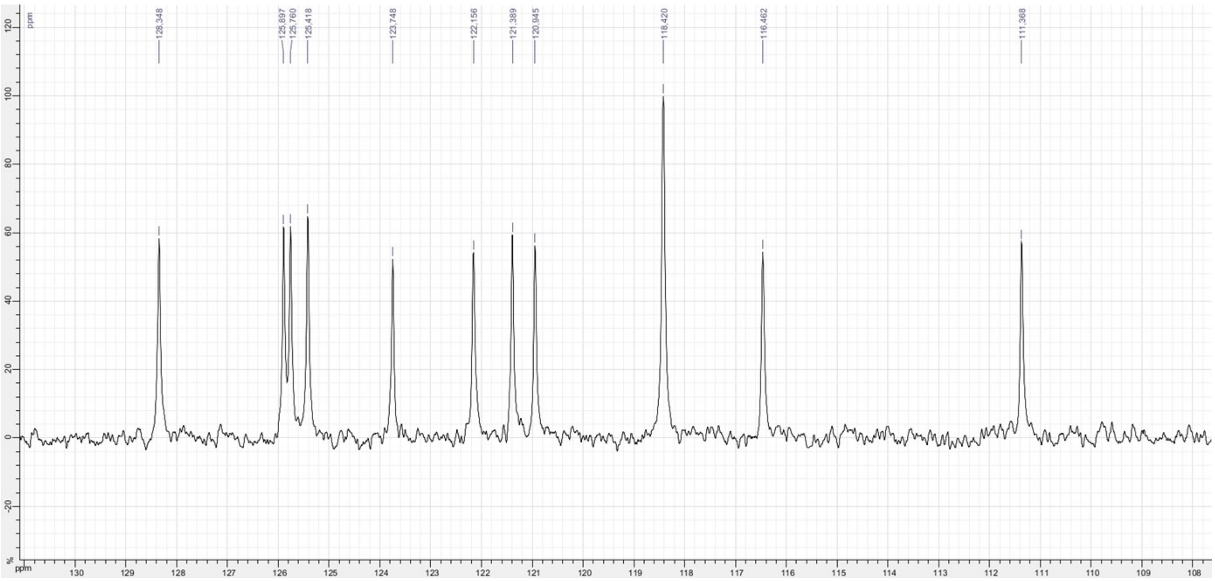

COSY NMR spectrum of **2f** in DMSO-d_6

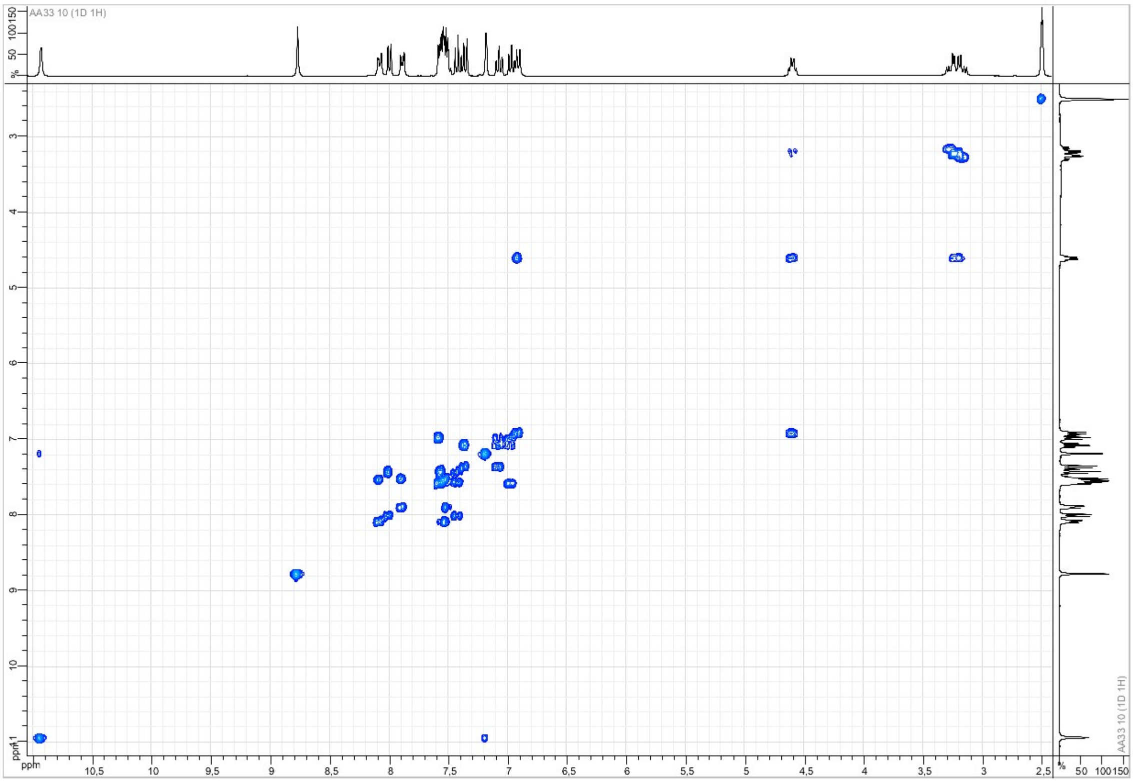

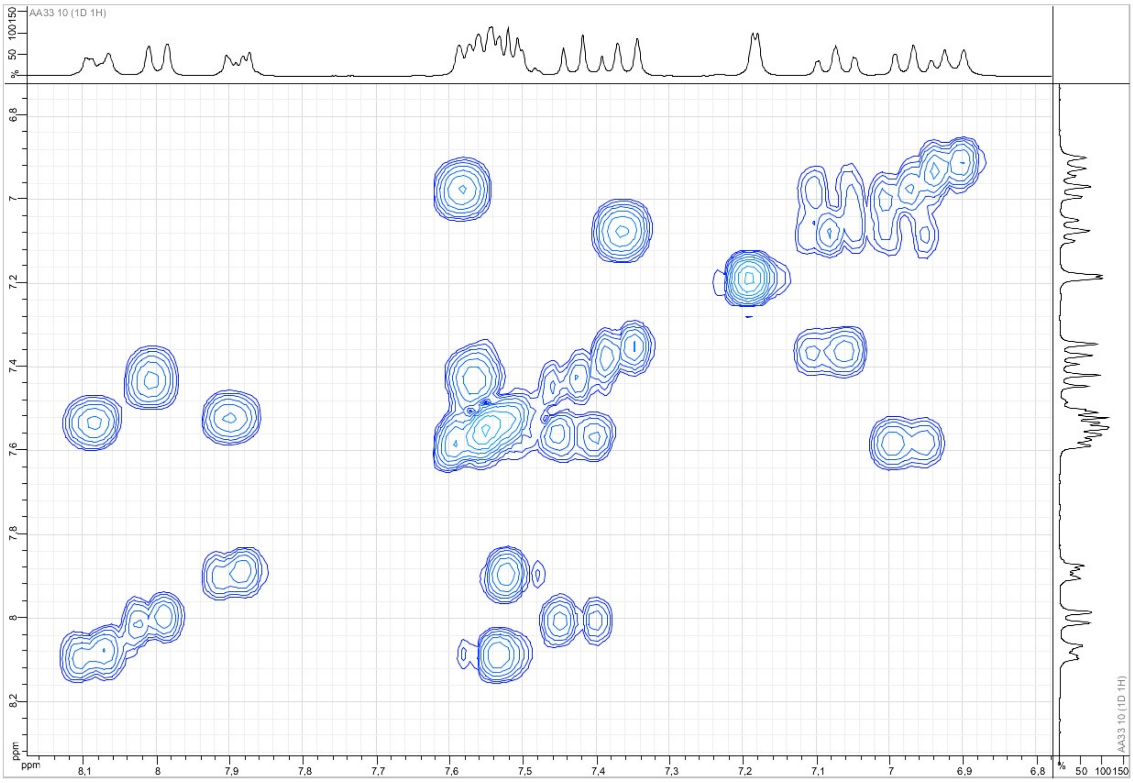

HSQC NMR spectrum of **2f** in DMSO-d_6

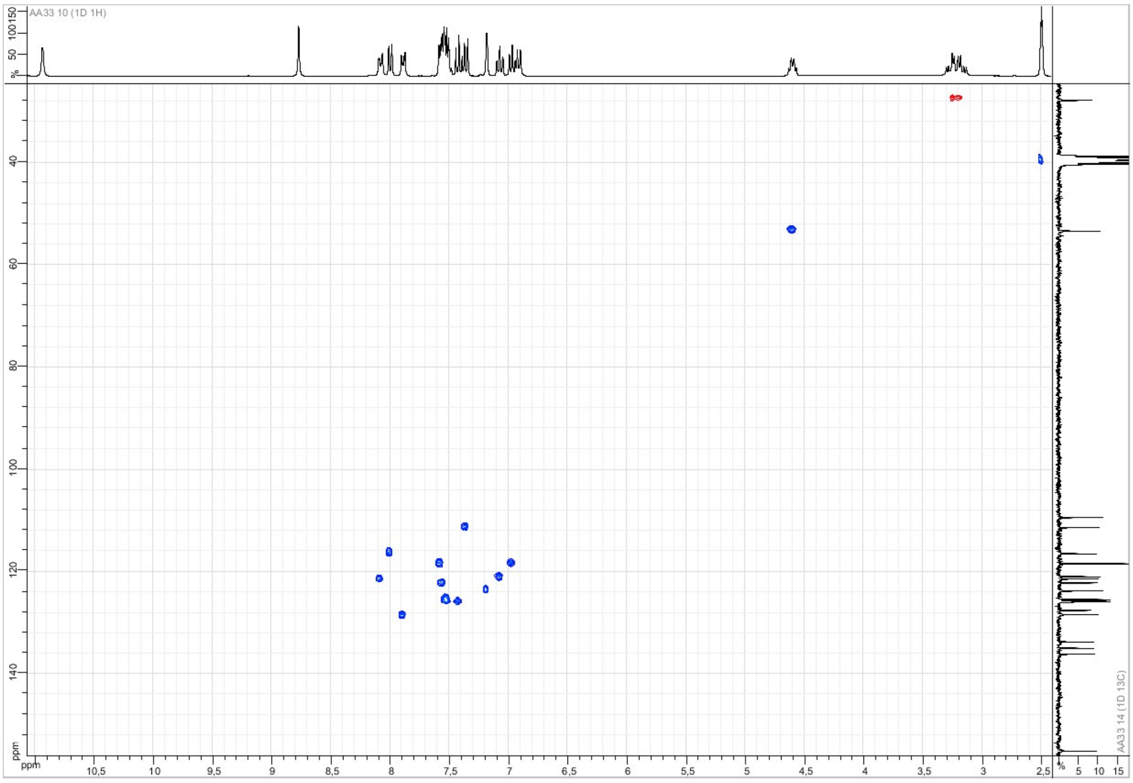

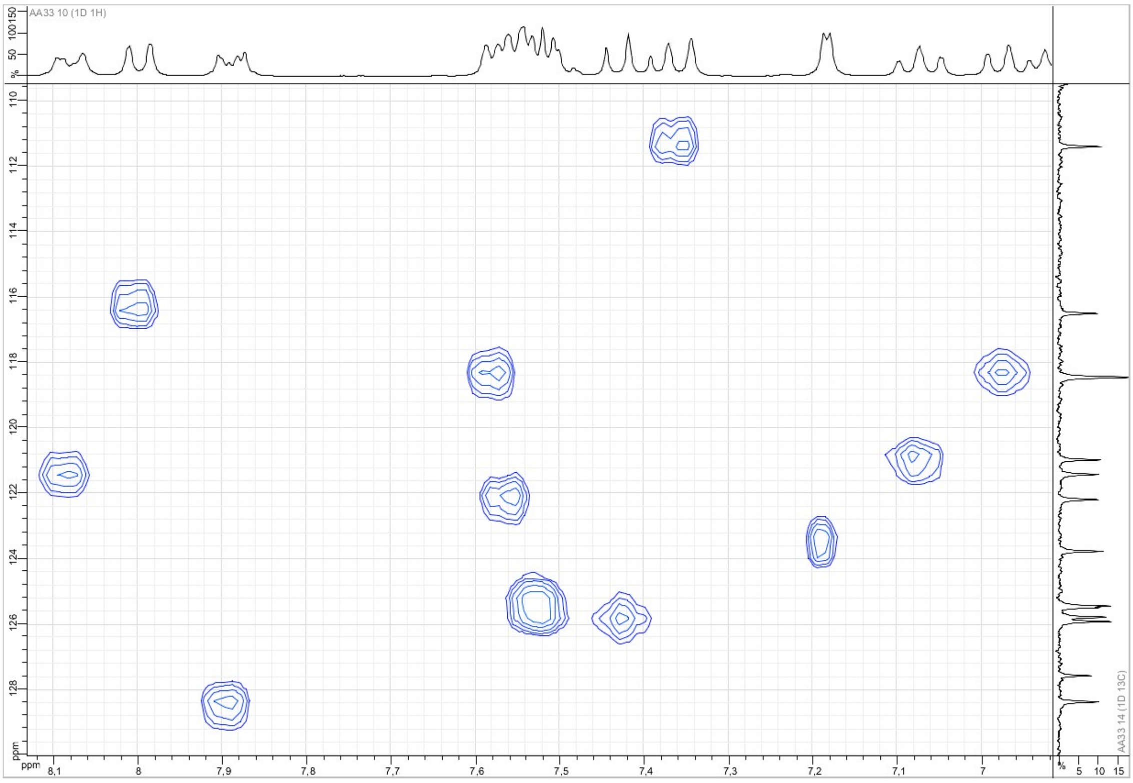

HMBC NMR spectrum of **2f** in DMSO-d_6

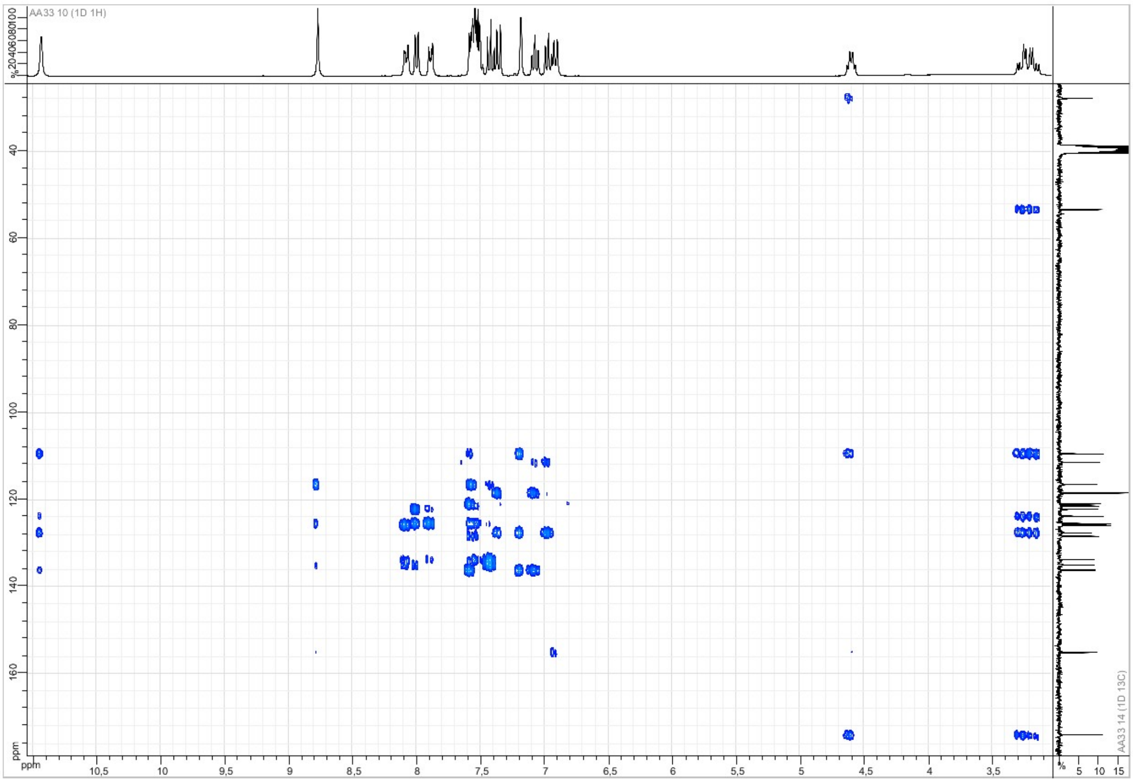

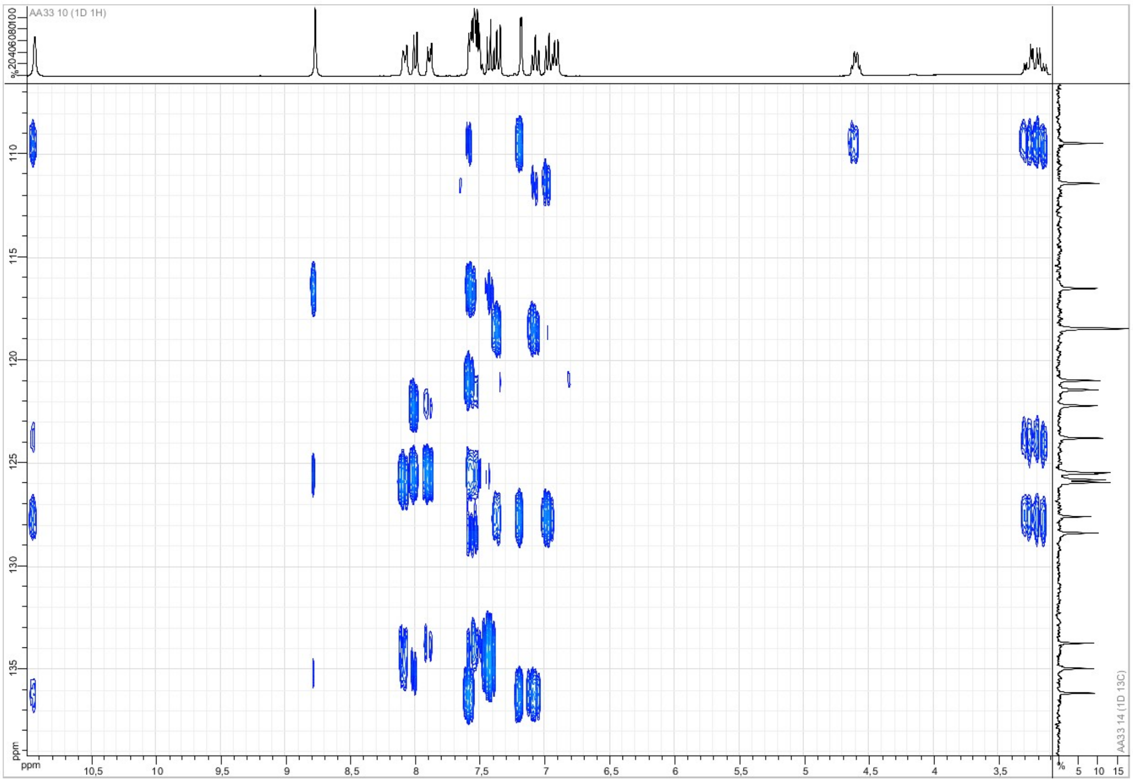

15. NMR spectra of 2g

^{1}H NMR (DMSO-d_6) of **2g** at 300 MHz

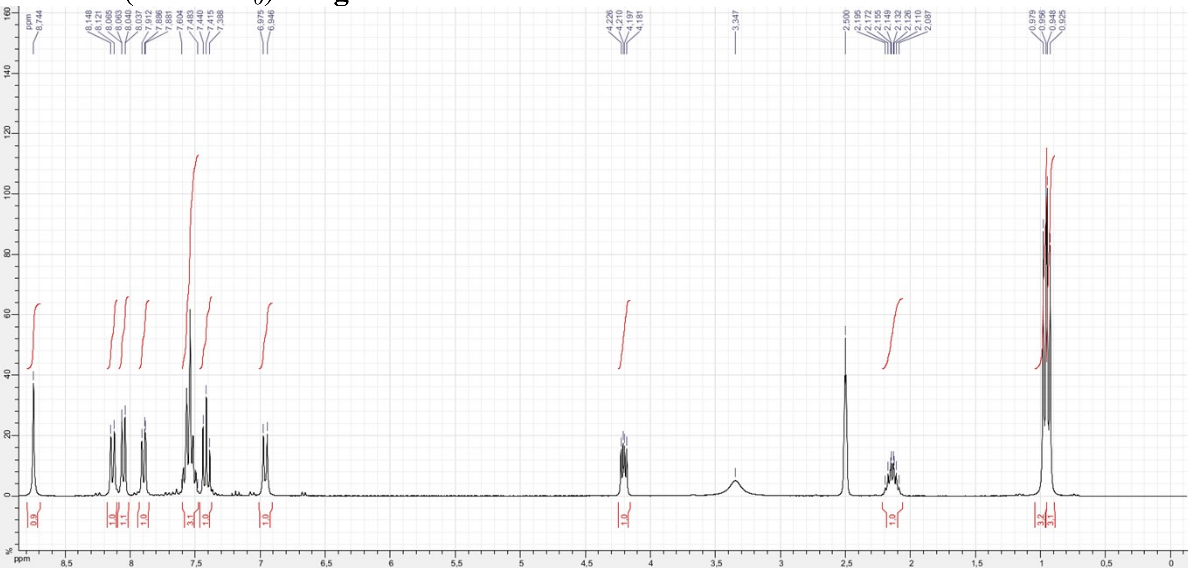

^{13}C NMR (DMSO-d_6) of **2g** at 75 MHz

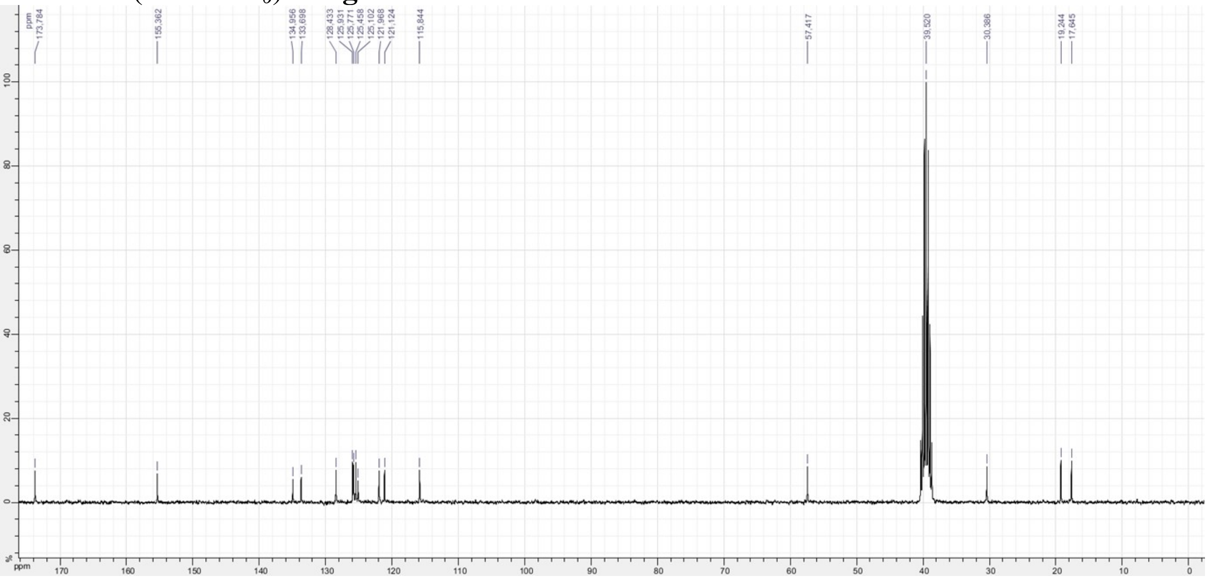

^{13}C (Dept135) NMR spectrum of **2g** in DMSO-d_6 at 75 MHz

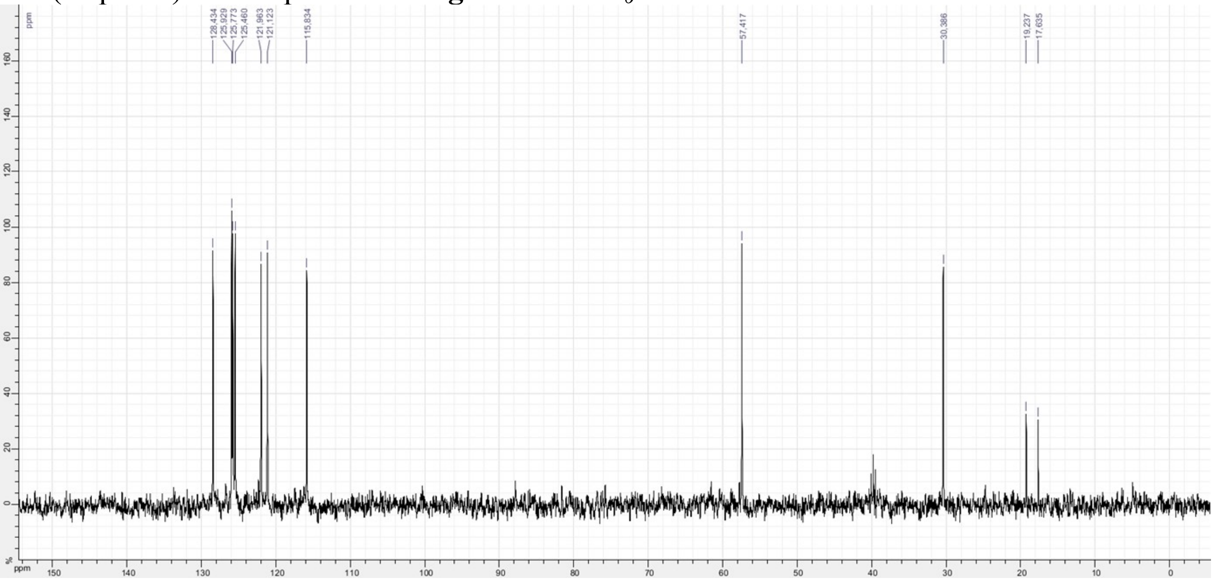

COSY NMR spectrum of **2g** in DMSO-d_6

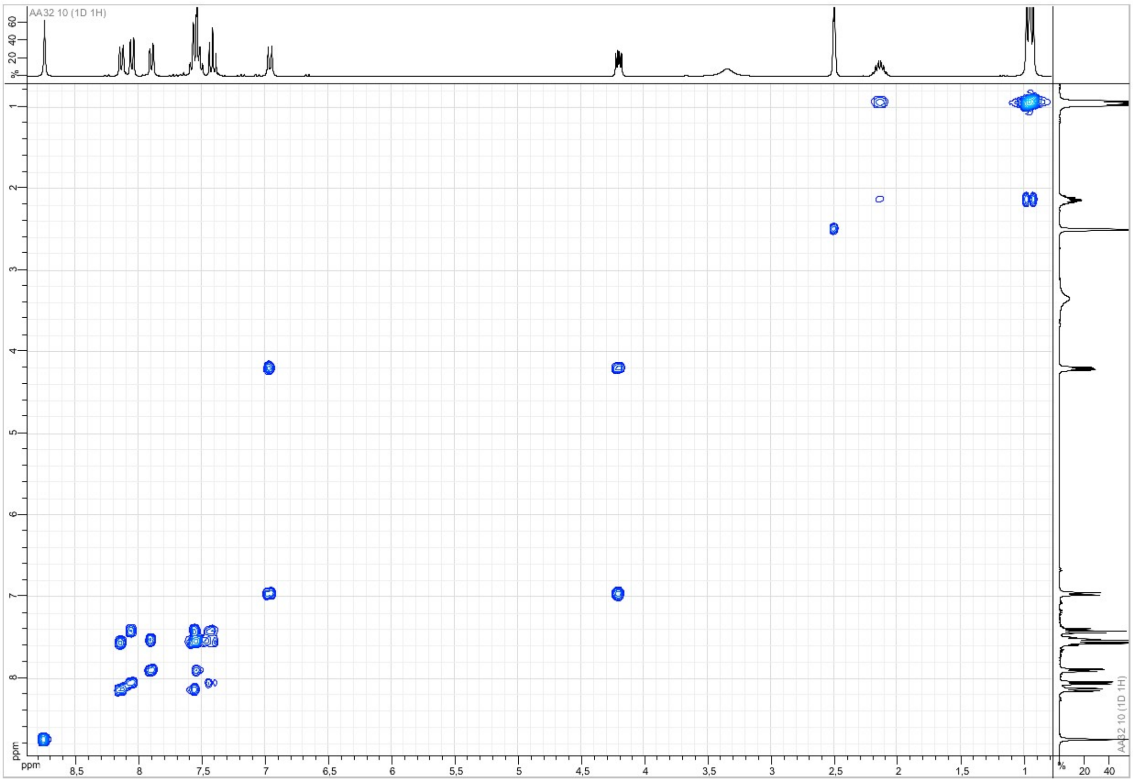

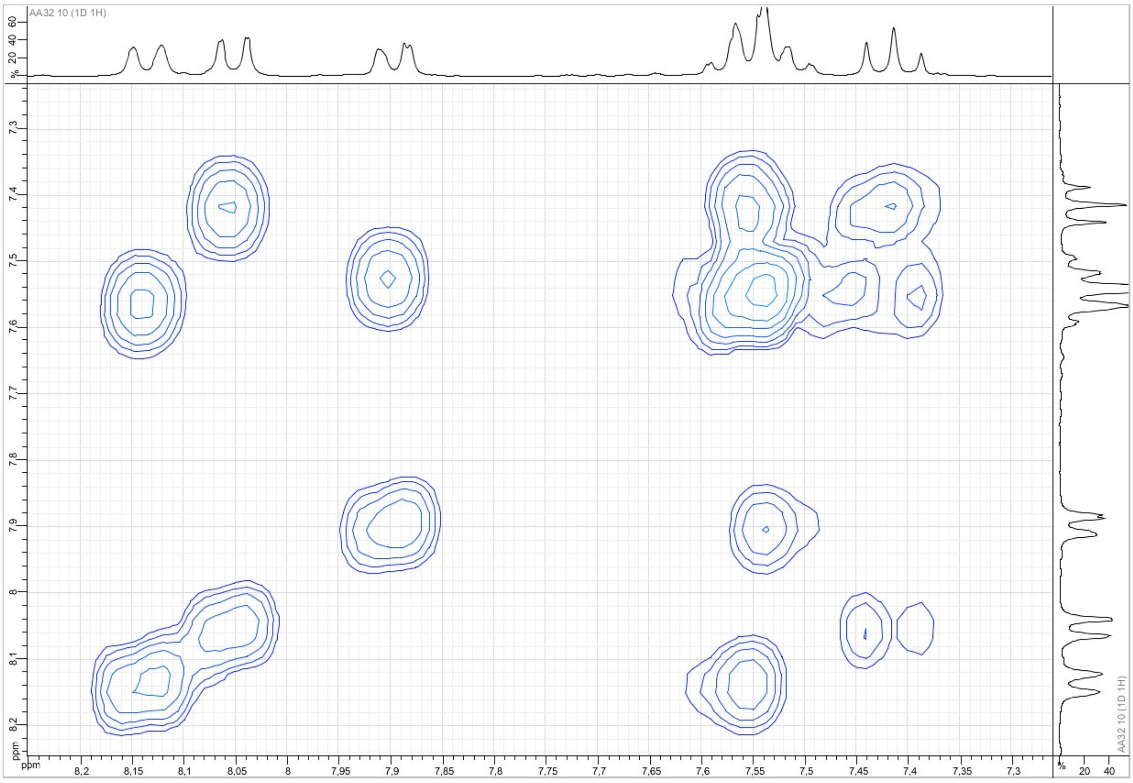

HSQC NMR spectrum of **2g** in DMSO-d_6

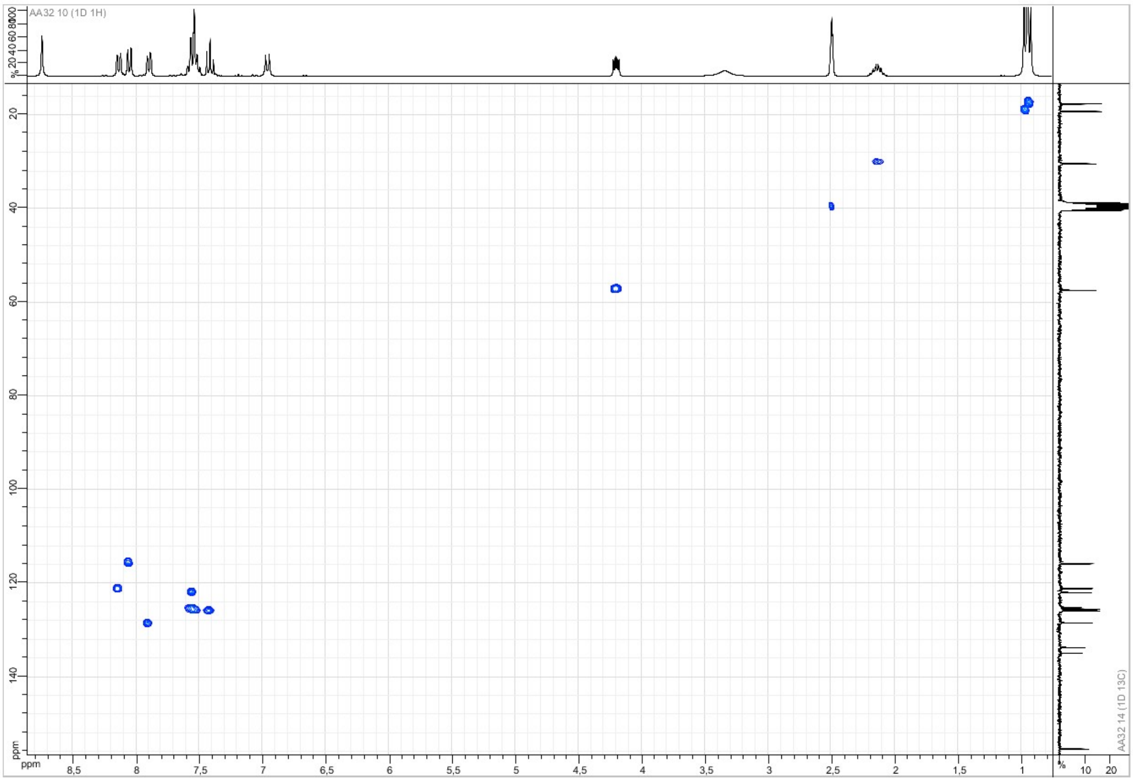

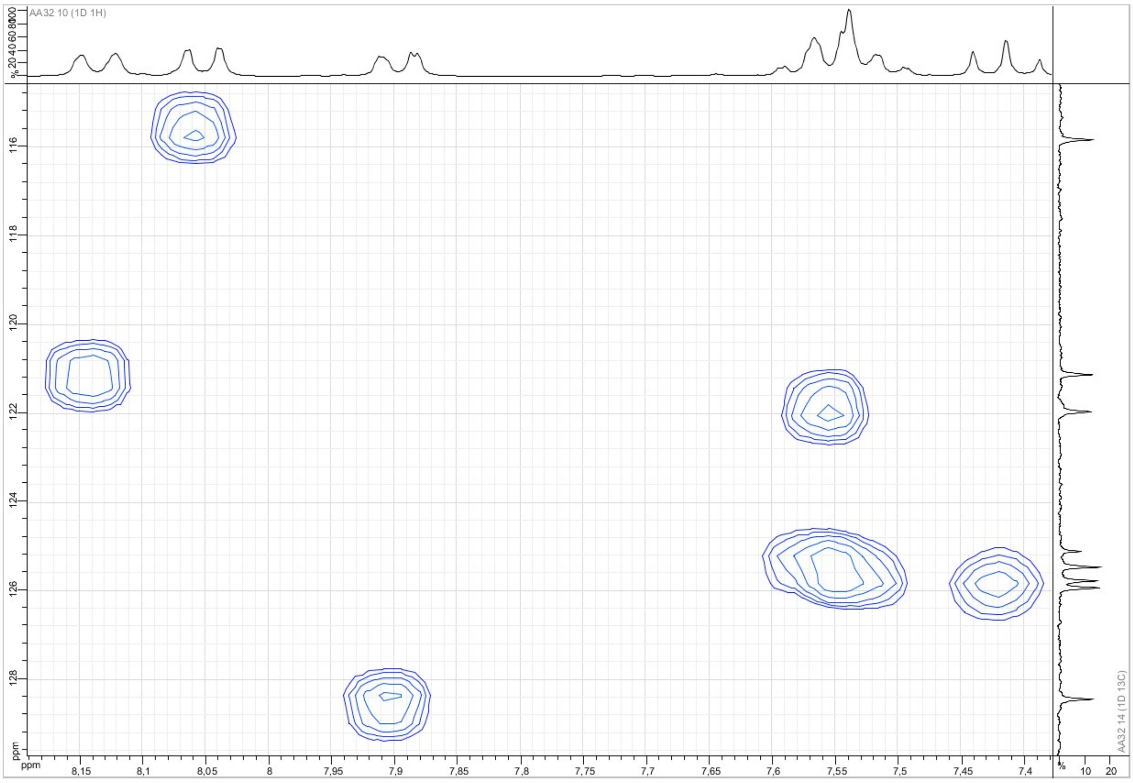

HMBC NMR spectrum of **2g** in DMSO-d_6

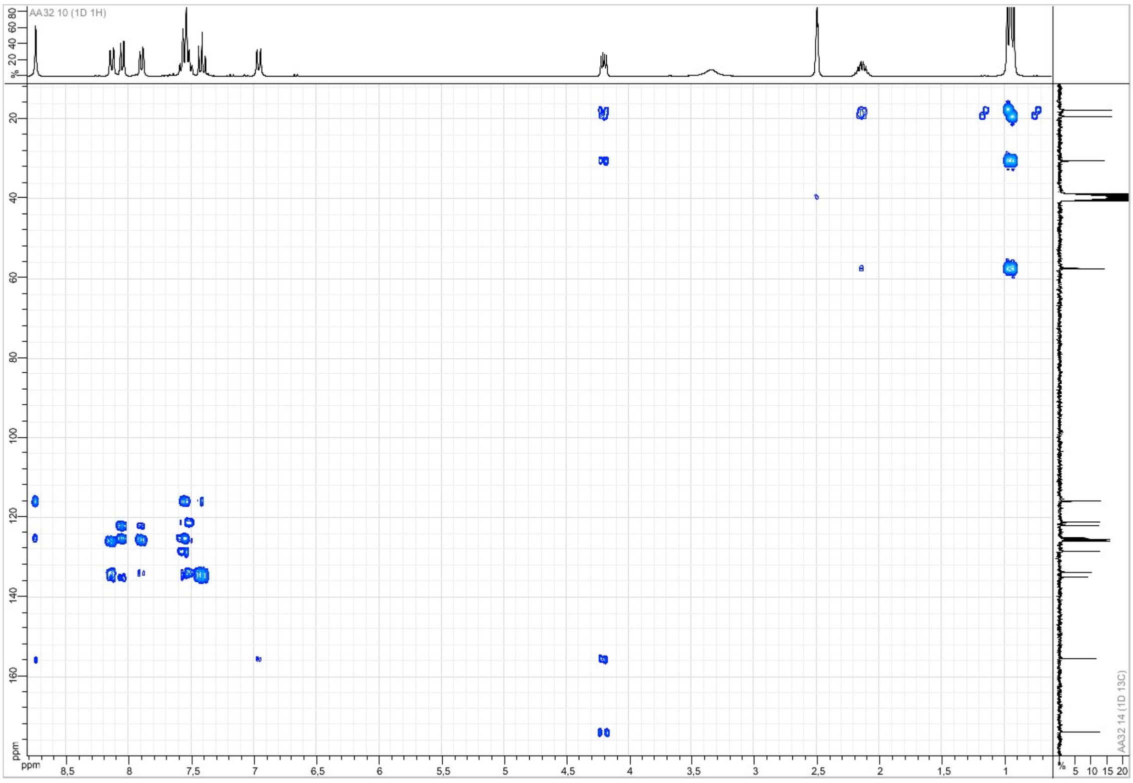

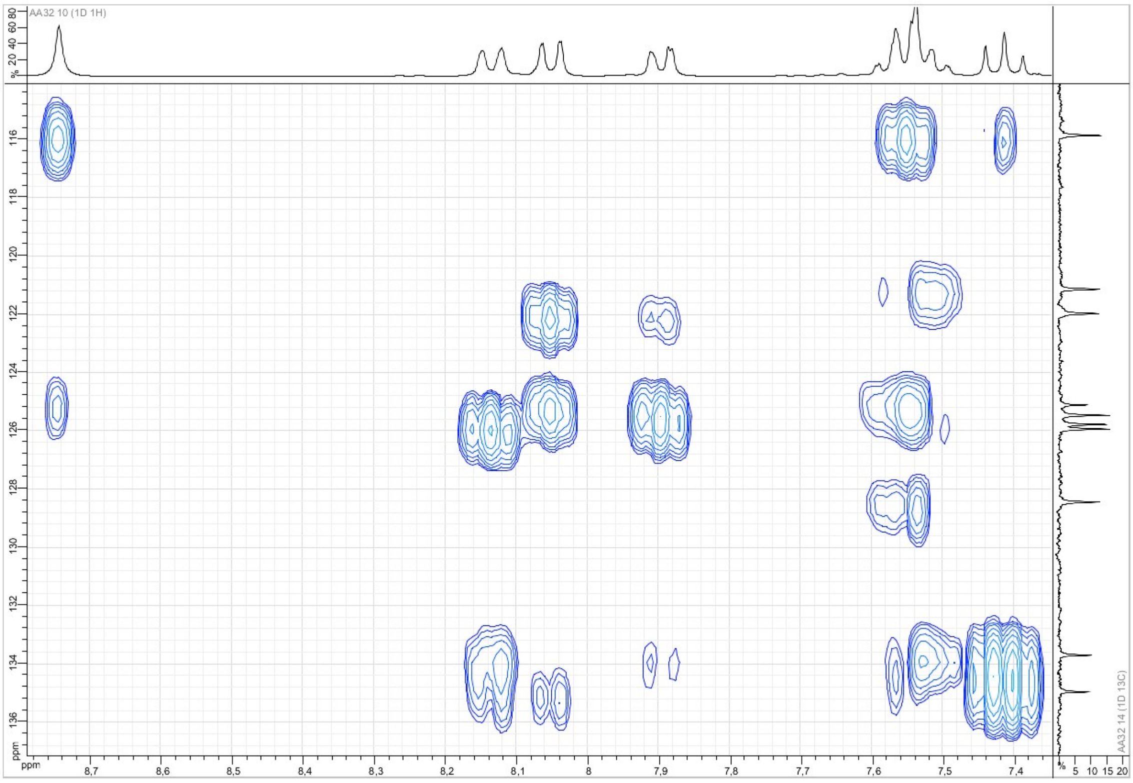

Part 2: Molecular docking

1. **The corresponding 2D diagram of the interactions of the compounds 1a-h, 2a-g and diclofenac at the active site of COX-1**

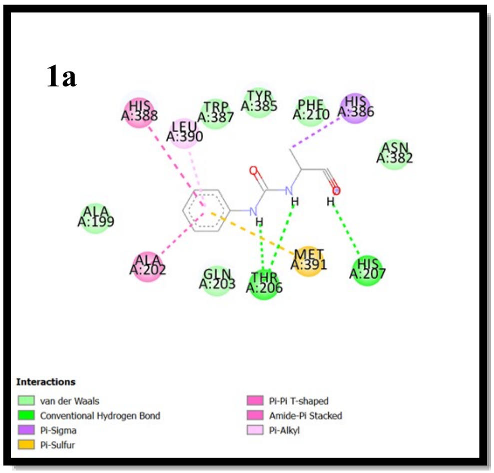

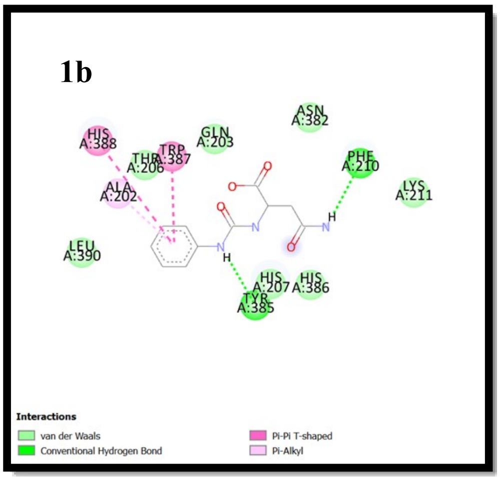

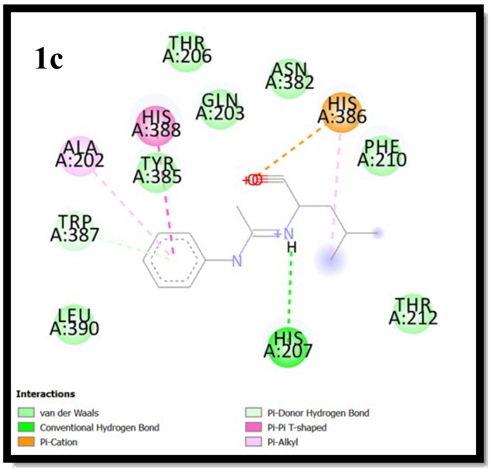

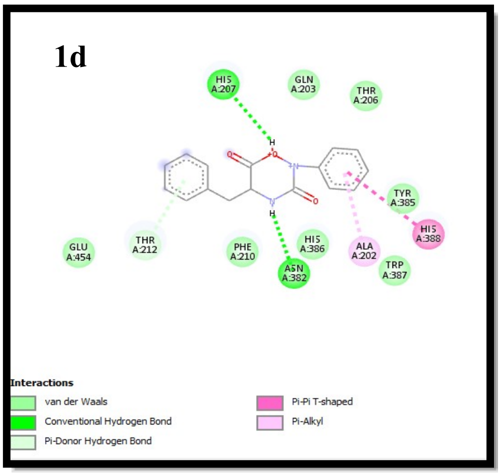

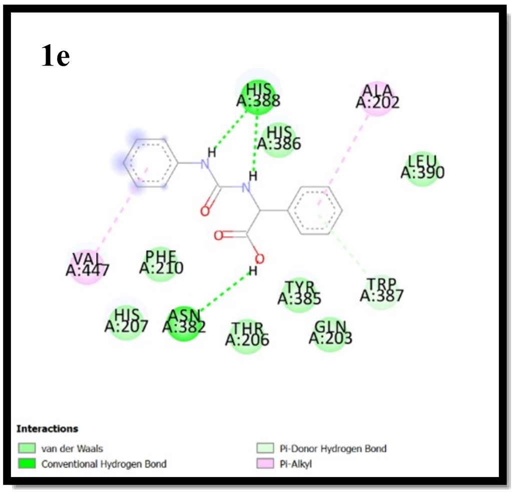
1e
HIS A:388
HIS A:386
ALA A:202
LEU A:390
VAL A:447
PHE A:210
TYR A:385
TRP A:387
HIS A:207
ASN A:382
THR A:206
GLN A:203
Interactions
van der Waals
Conventional Hydrogen Bond
Pi-Donor Hydrogen Bond
Pi-Alkyl

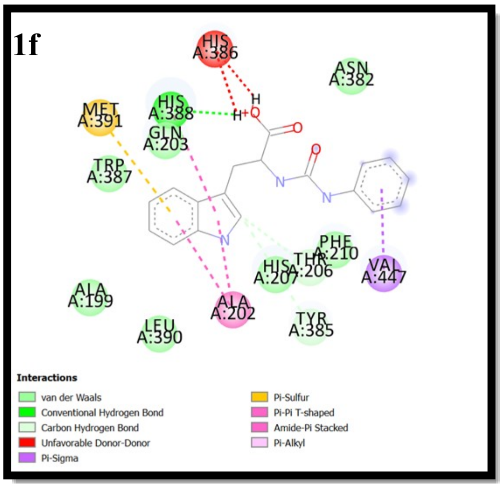
1f
HIS A:386
ASN A:382
MET A:391
HIS A:388
GLN A:203
TRP A:387
PHE A:210
HIS A:207
THR A:206
VAL A:447
ALA A:199
ALA A:202
TYR A:385
LEU A:390
Interactions
van der Waals
Conventional Hydrogen Bond
Carbon Hydrogen Bond
Unfavorable Donor-Donor
Pi-Sigma
Pi-Sulfur
Pi-Pi T-shaped
Amide-Pi Stacked
Pi-Alkyl

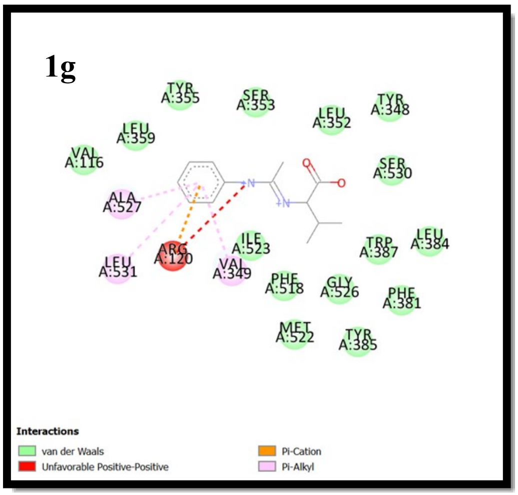
1g
TYR A:355
SER A:353
LEU A:352
TYR A:348
LEU A:359
VAL A:116
SER A:530
ALA A:527
ILE A:523
TRP A:387
LEU A:384
LEU A:531
ARG A:120
VAL A:349
PHE A:518
GLY A:526
PHE A:381
MET A:522
TYR A:385
Interactions
van der Waals
Unfavorable Positive-Positive
Pi-Cation
Pi-Alkyl

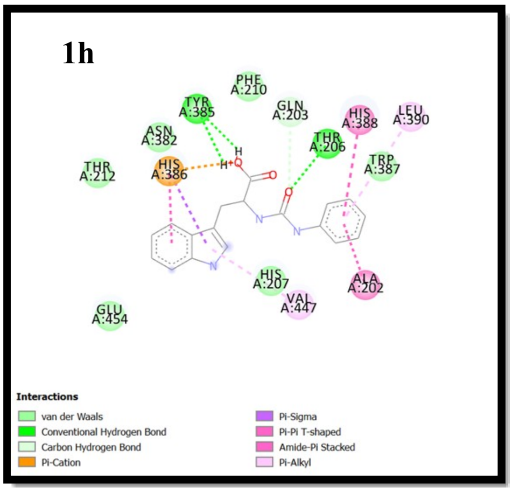
1h
PHE A:210
TYR A:385
GLN A:203
HIS A:388
LEU A:390
ASN A:382
THR A:206
THR A:212
HIS A:386
TRP A:387
HIS A:207
VAL A:447
ALA A:202
GLU A:454
Interactions
van der Waals
Conventional Hydrogen Bond
Carbon Hydrogen Bond
Pi-Cation
Pi-Sigma
Pi-Pi T-shaped
Amide-Pi Stacked
Pi-Alkyl

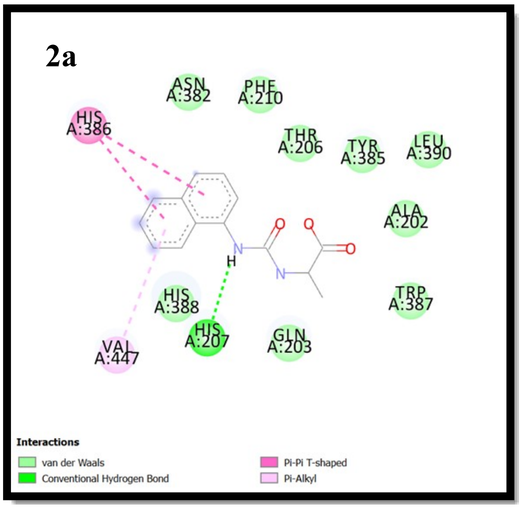
2a
ASN A:382
PHE A:210
HIS A:386
THR A:206
TYR A:385
LEU A:390
ALA A:202
HIS A:388
TRP A:387
HIS A:207
GLN A:203
VAL A:447
Interactions
van der Waals
Conventional Hydrogen Bond
Pi-Pi T-shaped
Pi-Alkyl

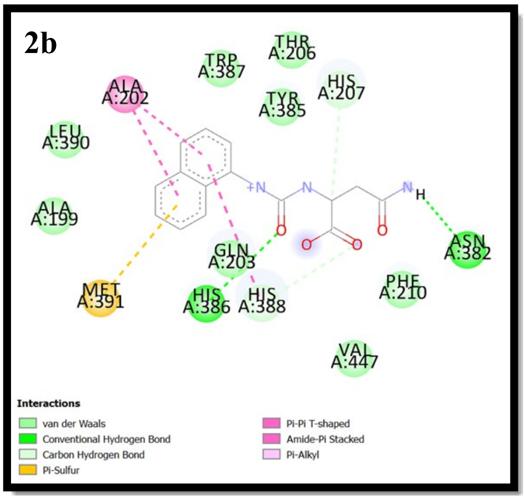
2b
THR A:206
TRP A:387
ALA A:202
HIS A:207
TYR A:385
LEU A:390
ALA A:199
ASN A:382
GLN A:203
MET A:391
HIS A:386
HIS A:388
PHE A:210
VAL A:447
Interactions
van der Waals
Conventional Hydrogen Bond
Carbon Hydrogen Bond
Pi-Sulfur
Pi-Pi T-shaped
Amide-Pi Stacked
Pi-Alkyl

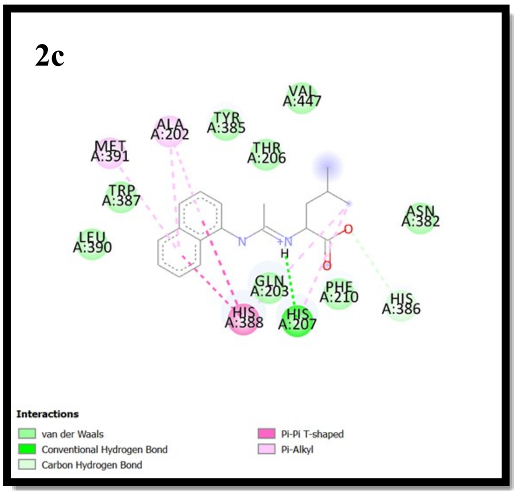
2c
Interactions
van der Waals
Conventional Hydrogen Bond
Carbon Hydrogen Bond
Pi-Pi T-shaped
Pi-Alkyl

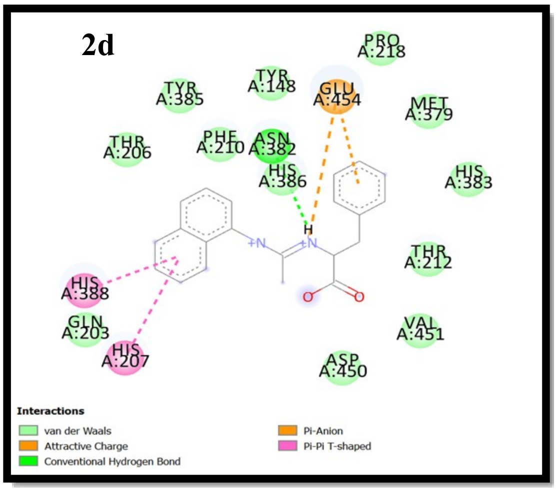
2d
Interactions
van der Waals
Attractive Charge
Conventional Hydrogen Bond
Pi-Anion
Pi-Pi T-shaped

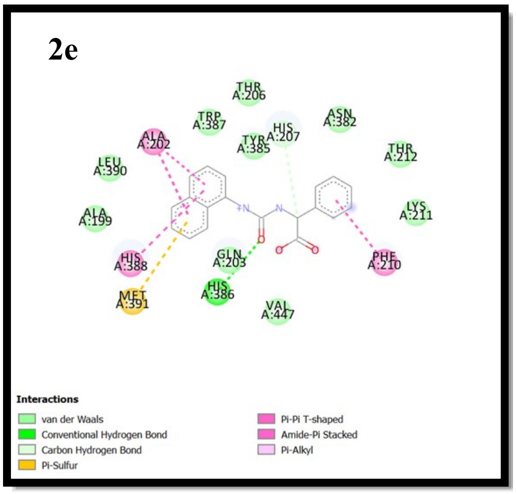
2e
Interactions
van der Waals
Conventional Hydrogen Bond
Carbon Hydrogen Bond
Pi-Sulfur
Pi-Pi T-shaped
Amide-Pi Stacked
Pi-Alkyl

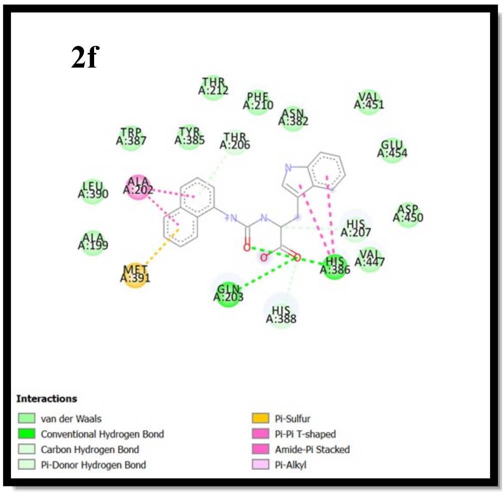
2f
Interactions
van der Waals
Conventional Hydrogen Bond
Carbon Hydrogen Bond
Pi-Donor Hydrogen Bond
Pi-Sulfur
Pi-Pi T-shaped
Amide-Pi Stacked
Pi-Alkyl

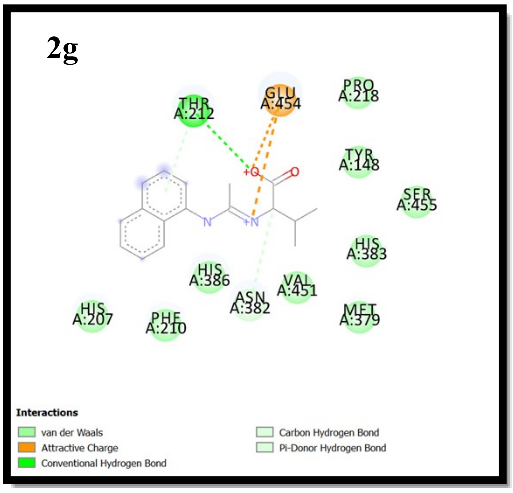
2g
Interactions
van der Waals
Attractive Charge
Conventional Hydrogen Bond
Carbon Hydrogen Bond
Pi-Donor Hydrogen Bond

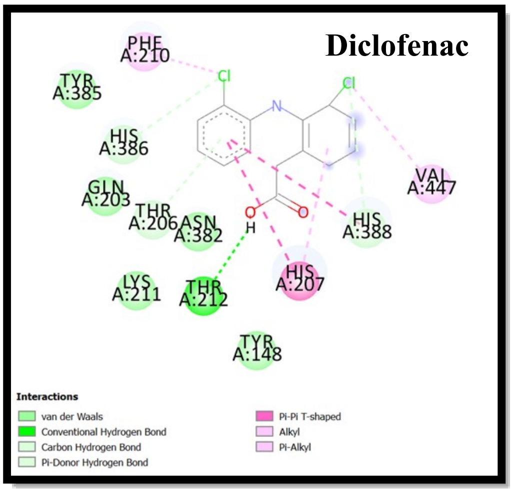
Diclofenac
Interactions
van der Waals
Conventional Hydrogen Bond
Carbon Hydrogen Bond
Pi-Donor Hydrogen Bond
Pi-Pi T-shaped
Alkyl
Pi-Alkyl

2. The corresponding 2D diagram of the interactions of the compounds 1a-h, 2a-g and diclofenac at the active site of COX-2

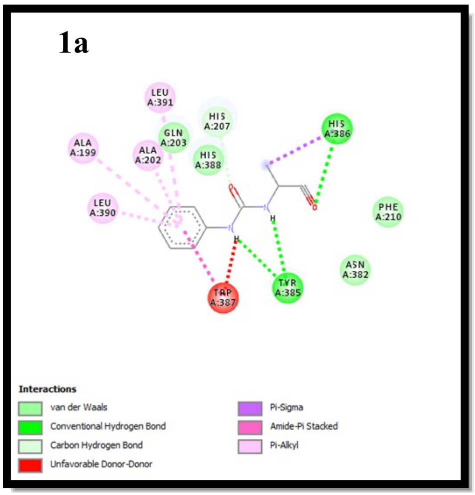

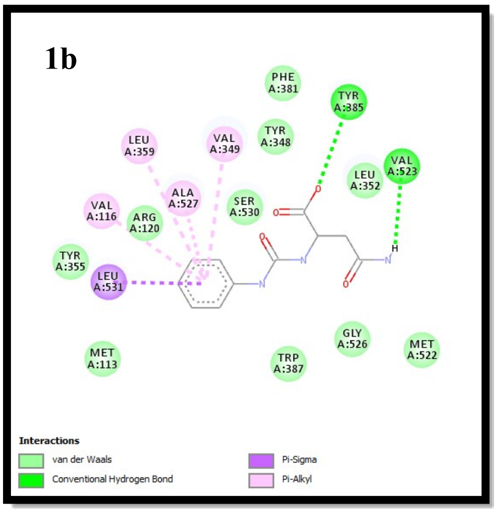

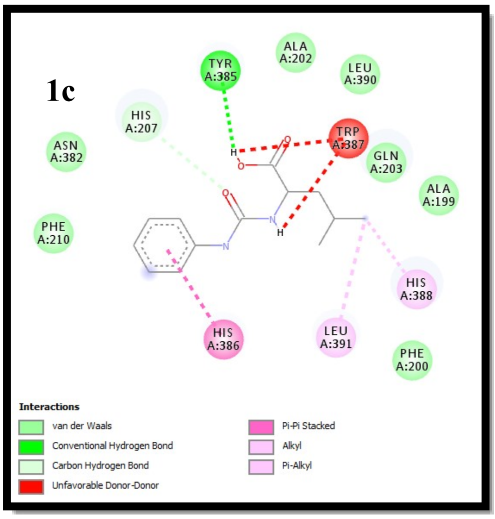

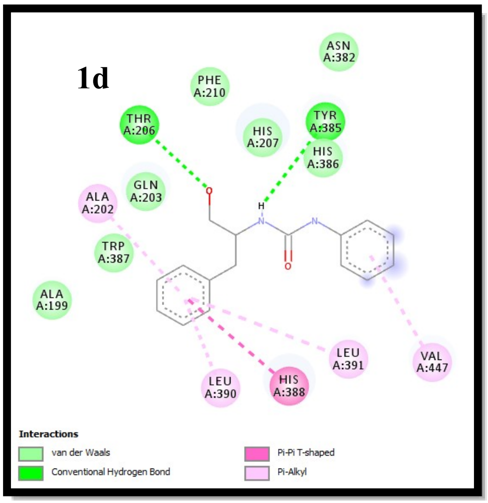

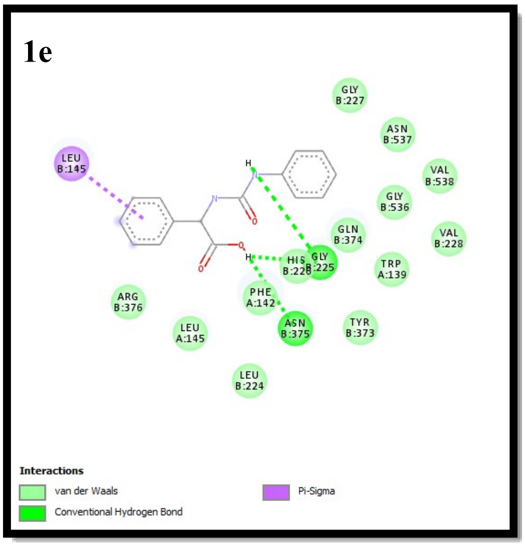
1e
GLY B:227
ASN B:537
LEU B:145
VAL B:538
GLY B:536
GLN B:374
VAL B:228
HIS B:226
GLY B:225
TRP B:139
PHE A:142
ARG B:376
ASN B:375
TYR B:373
LEU A:145
LEU B:224
Interactions
van der Waals
Conventional Hydrogen Bond
Pi-Sigma

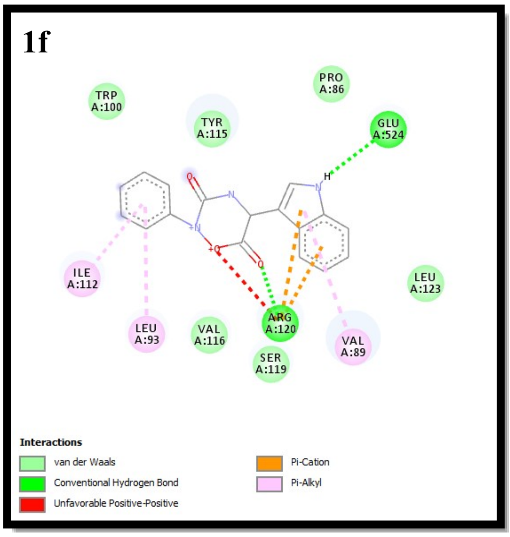
1f
PRO A:86
TRP A:100
TYR A:115
GLU A:524
ILE A:112
LEU A:123
LEU A:93
VAL A:116
ARG A:120
VAL A:89
SER A:119
Interactions
van der Waals
Conventional Hydrogen Bond
Unfavorable Positive-Positive
Pi-Cation
Pi-Alkyl

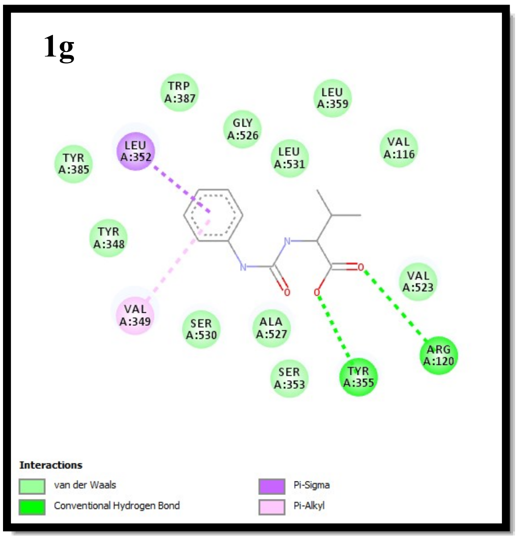
1g
TRP A:387
LEU A:359
GLY A:526
TYR A:385
LEU A:352
LEU A:531
VAL A:116
TYR A:348
VAL A:523
VAL A:349
SER A:530
ALA A:527
ARG A:120
SER A:353
TYR A:355
Interactions
van der Waals
Conventional Hydrogen Bond
Pi-Sigma
Pi-Alkyl

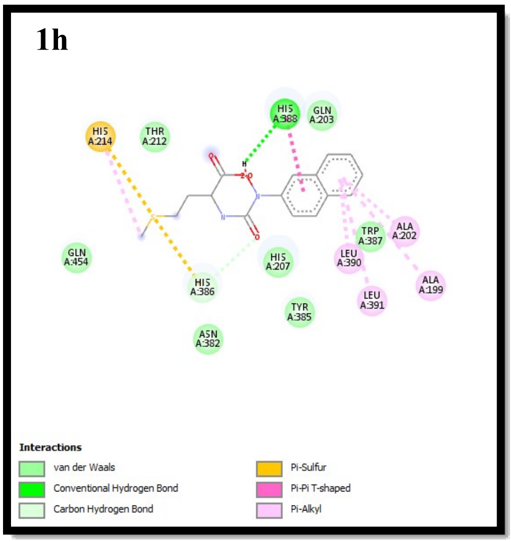
1h
HIS A:388
GLN A:203
HIS A:214
THR A:212
TRP A:387
ALA A:202
GLN A:454
HIS A:207
LEU A:390
HIS A:386
ALA A:199
LEU A:391
TYR A:385
ASN A:382
Interactions
van der Waals
Conventional Hydrogen Bond
Carbon Hydrogen Bond
Pi-Sulfur
Pi-Pi T-shaped
Pi-Alkyl

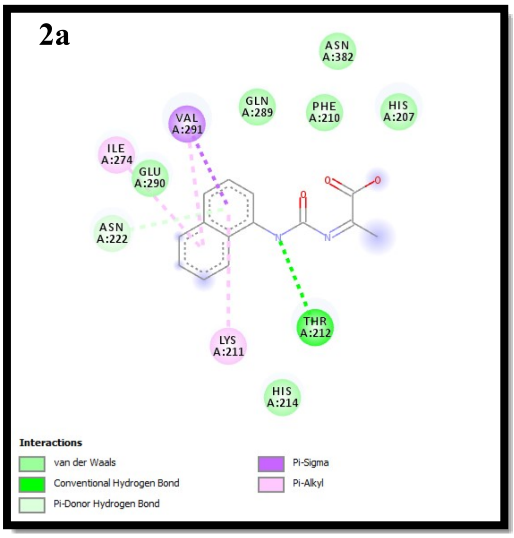
2a
ASN A:382
GLN A:289
PHE A:210
HIS A:207
VAL A:291
ILE A:274
GLU A:290
ASN A:222
THR A:212
LYS A:211
HIS A:214
Interactions
van der Waals
Conventional Hydrogen Bond
Pi-Donor Hydrogen Bond
Pi-Sigma
Pi-Alkyl

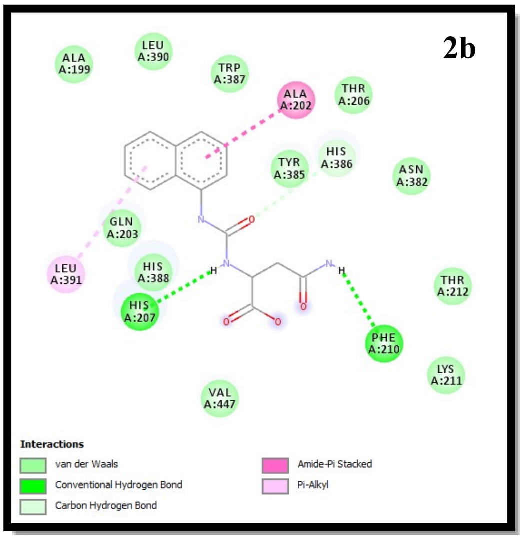
2b
LEU A:390
ALA A:199
TRP A:387
ALA A:202
THR A:206
TYR A:385
HIS A:386
ASN A:382
GLN A:203
HIS A:388
LEU A:391
HIS A:207
THR A:212
PHE A:210
LYS A:211
VAL A:447
Interactions
van der Waals
Conventional Hydrogen Bond
Carbon Hydrogen Bond
Amide-Pi Stacked
Pi-Alkyl

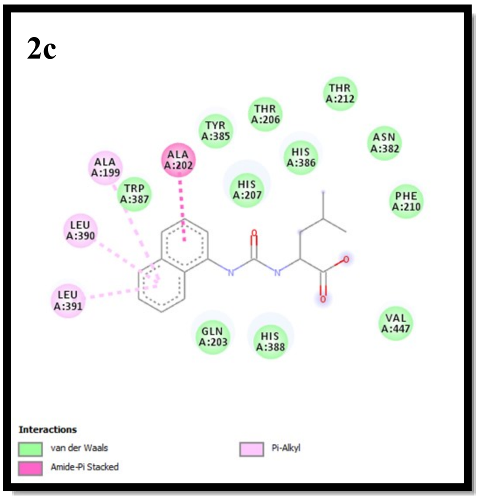
2c
THR A:212
THR A:206
TYR A:385
ASN A:382
HIS A:386
ALA A:199
ALA A:202
TRP A:387
HIS A:207
PHE A:210
LEU A:390
LEU A:391
VAL A:447
GLN A:203
HIS A:388
Interactions
van der Waals
Amide-Pi Stacked
Pi-Alkyl

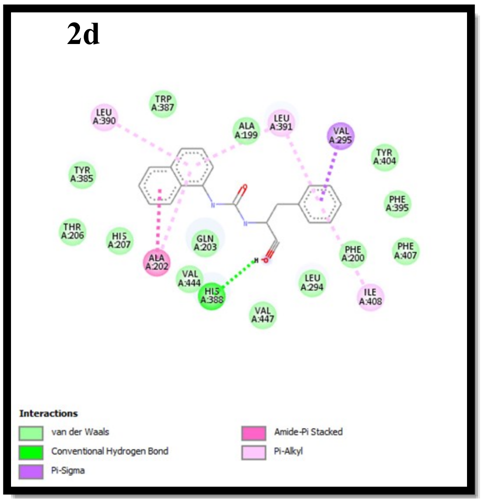
2d
TRP A:387
LEU A:390
ALA A:199
LEU A:391
VAL A:295
TYR A:404
TYR A:385
PHE A:395
THR A:206
HIS A:207
GLN A:203
PHE A:200
PHE A:407
ALA A:202
VAL A:444
HIS A:388
LEU A:294
ILE A:408
VAL A:447
Interactions
van der Waals
Conventional Hydrogen Bond
Pi-Sigma
Amide-Pi Stacked
Pi-Alkyl

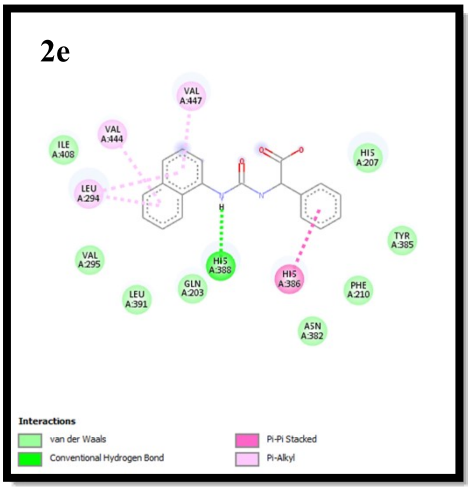
2e
VAL A:447
VAL A:444
ILE A:408
HIS A:207
LEU A:294
TYR A:385
VAL A:295
HIS A:388
HIS A:386
PHE A:210
LEU A:391
GLN A:203
ASN A:382
Interactions
van der Waals
Conventional Hydrogen Bond
Pi-Pi Stacked
Pi-Alkyl

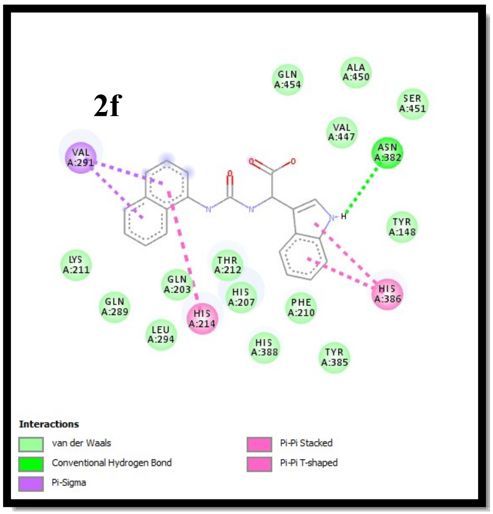
2f
GLN A:454
ALA A:450
SER A:451
VAL A:447
VAL A:291
ASN A:382
TYR A:148
LYS A:211
THR A:212
GLN A:203
HIS A:207
HIS A:386
GLN A:289
HIS A:214
PHE A:210
LEU A:294
HIS A:388
TYR A:385
Interactions
van der Waals
Conventional Hydrogen Bond
Pi-Sigma
Pi-Pi Stacked
Pi-Pi T-shaped

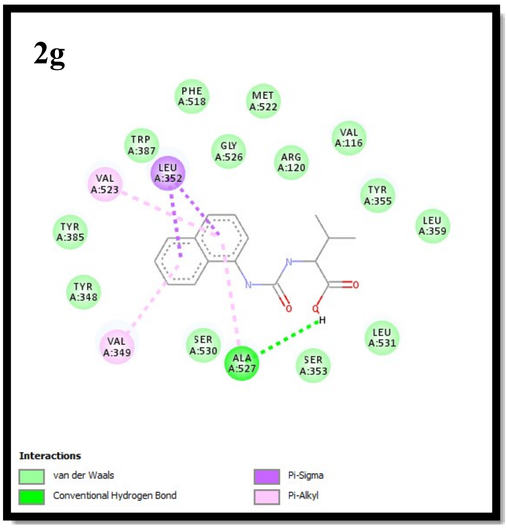
2g
PHE A:518
MET A:522
TRP A:387
VAL A:116
GLY A:526
ARG A:120
LEU A:352
VAL A:523
TYR A:355
LEU A:359
TYR A:385
TYR A:348
VAL A:349
SER A:530
ALA A:527
SER A:353
LEU A:531
Interactions
van der Waals
Conventional Hydrogen Bond
Pi-Sigma
Pi-Alkyl

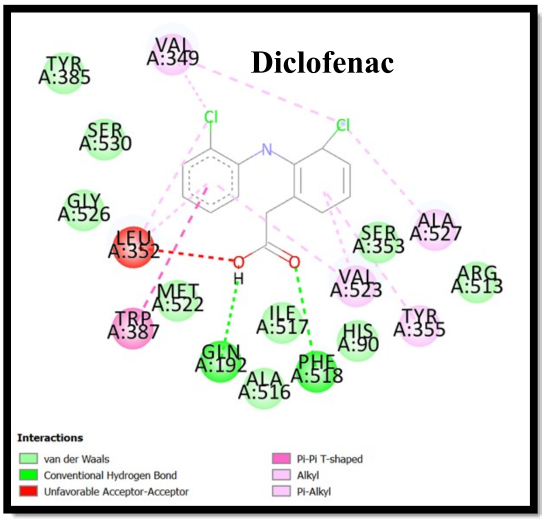
Diclofenac
TYR A:385
VAL A:349
SER A:530
GLY A:526
LEU A:352
SER A:353
ALA A:527
VAL A:523
ARG A:513
MET A:522
TRP A:387
ILE A:517
HIS A:90
TYR A:355
GLN A:192
ALA A:516
PHE A:518
Interactions
van der Waals
Conventional Hydrogen Bond
Unfavorable Acceptor-Acceptor
Pi-Pi T-shaped
Alkyl
Pi-Alkyl
